# Diversity of options to eliminate fossil fuels and reach carbon neutrality across the entire European energy system

**DOI:** 10.1016/j.joule.2022.05.009

**Published:** 2022-06-15

**Authors:** Bryn Pickering, Francesco Lombardi, Stefan Pfenninger

**Affiliations:** 1 Institute for Environmental Decisions, Department for Environmental Systems Science, ETH Zürich, Zürich, Switzerland; 2 Faculty of Technology, Policy and Management (TPM), Delft University of Technology, Delft, the Netherlands

**Keywords:** Carbon neutrality, near-optimal solutions, modeling to generate alternatives, SPORES, Calliope, energy self-sufficiency, sector coupling, energy system optimisation, flexibility, renewable energy

## Abstract

Disagreements persist on how to design a self-sufficient, carbon-neutral European energy system. To explore the diversity of design options, we develop a high-resolution model of the entire European energy system and produce 441 technically feasible system designs that are within 10% of the optimal economic cost. We show that a wide range of systems based on renewable energy are feasible, with no need to import energy from outside Europe. Model solutions reveal considerable flexibility in the choice and geographical distribution of new infrastructure across the continent. Balanced renewable energy supply can be achieved either with or without mechanisms such as biofuel use, curtailment, and expansion of the electricity network. Trade-offs emerge once specific preferences are imposed. Low biofuel use, for example, requires heat electrification and controlled vehicle charging. This exploration of the impact of preferences on system design options is vital to inform urgent, politically difficult decisions for eliminating fossil fuel imports and achieving European carbon neutrality.

## Highlights

• Many cost-effective options exist for an energy self-sufficient, carbon-neutral Europe
• A variety of spatial configurations are valid, and specific regions can be prioritized
• Although firm capacity is not a must have, limiting it reduces the maneuvering space
• For example, low bioenergy requires electrified heat and controlled vehicle charging

## Context & scale

Most studies to reach European carbon -neutrality focus on one or a few economically “optimal” scenarios, suggesting that only these system design options exist. We show a diversity of untold options to meet all energy demand based on renewable energy, with a complete phase-out of oil and gas imports. With a marginal increase above optimal cost, the reliance of an energy self-sufficient Europe on specific solutions, like biofuels, battery storage, transmission expansion, or heat electrification, can vary from not being used at all to being key to system stability.

With our work, policy makers can explore this option space. For instance, one can investigate where to locate hydrogen production hubs or look at the necessary trade-offs of imposing preferences like having to support consumers in electrifying heat and transport if biofuel use is minimized. Our code and data are open; hence, our approach can be applied to other continents or scaled to support decisions in specific regions.

## Graphical abstract

**Figure:**
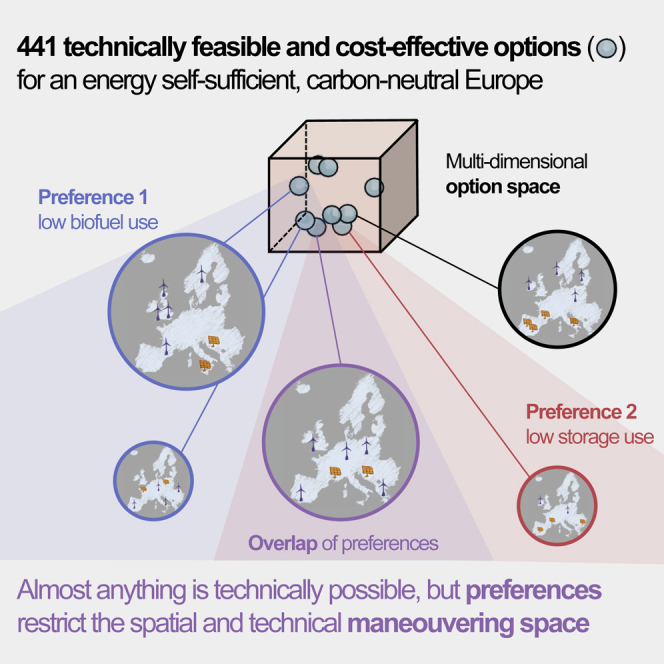

## Teaser

Many technical options exist to achieve carbon neutrality across all energy sectors and to completely eliminate fossil fuel imports in Europe. The energy system can be designed to rely to a varying extent on wind, solar, biofuels, and other technologies, and the infrastructure can be located in different regions. However, imposing specific preferences, such as biofuel dependence, reduces the maneuvering space for other decisions. In all cases, the scale of the transformation remains enormous and requires acceleration.

## Introduction

There is disagreement between models about what technical solutions are viable to achieve a carbon-neutral European energy system. Many studies have focused on a highly renewable electricity supply,^1^, ^2^, ^3^, ^4^, ^5^ which has emerged as a credible way to achieve carbon-neutral energy, given the dramatic cost reductions of wind and photovoltaic power generation over the past decade.^6^, ^7^, ^8^ However, techno-economic models designed primarily to understand the system integration of variable renewable generation use a high spatiotemporal resolution at the expense of considering the energy system beyond only electricity^1^^,^^9^^,^^10^ or by making simplifying assumptions on full electrification of some sectors, leaving aside the role of non-electric carbon-neutral solutions.^2^^,^^11^^,^^12^ Since there are in fact many non-electric energy end-uses,^2^^,^^13^, ^14^, ^15^ these models often underestimate the scale of the transition and the extent to which Europe may need to remain dependent on fuel imports to meet energy demands.

In contrast, integrated assessment and energy-environment-economy models designed to understand climate mitigation pathways consider many interactions between human and earth systems, which includes all energy demands globally. However, they do this at the expense of spatiotemporal detail. Therefore, they underestimate the potential for sector coupling to help balance renewable variability.^16^ The result is that pathway end-states from these models have generally contradicted the system designs from the former group of models, by suggesting a significant need for firm capacity, including fossil-fired generation with carbon capture and storage (CCS).^8^^,^^17^, ^18^, ^19^

There is an additional drawback to most existing modeling studies: they generally consider a single, cost-optimal solution, or a limited set of cost-optimal scenarios. The growing field of modeling to generate alternatives (MGA) has shown how the realistic decision space is much broader.^20^, ^21^, ^22^ However, no study has yet applied this approach to a Europe-wide high-resolution model including all energy demands.

Here, we address this gap to answer the question—what is the possible technological and spatial diversity in a self-sufficient and carbon-neutral European energy system based largely on renewable electricity generation? In doing so, we develop a model to represent all energy-consuming sectors in Europe with high resolution. We include demand in residential and commercial buildings; industry processes and feedstocks; passenger and freight transport by road, rail, air, and sea; and public services, agriculture, fisheries, and military facilities. We use a collection of novel methods to model demand and supply options across Europe at a high spatial and temporal resolution, tracking flows for electricity, heat, mobility, hydrogen, synthetic hydrocarbons, residual biofuels, and municipal waste. We summarize the main innovations and features of our model below and document them in detail in Note S1.

We use our model to explore the near-optimal decision space of an energy self-sufficient, carbon-neutral Europe and quantify trade-offs between competing interests according to nine high-level system metrics. We finish by discussing the implications of our exposed option space on the decision-making process. Given the extent of the option space, we cannot examine all trade-offs here. However, we release all of our results freely on Zenodo: https://doi.org/10.5281/zenodo.6546817, and our interactive web application at https://explore.callio.pe allows researchers and decision-makers to explore the impact of their preferences on the features of a self-sufficient, carbon-neutral European energy system.

## Sector-coupled energy service demand is up to 2.85× higher than electricity-only demand

**Figure 1:**
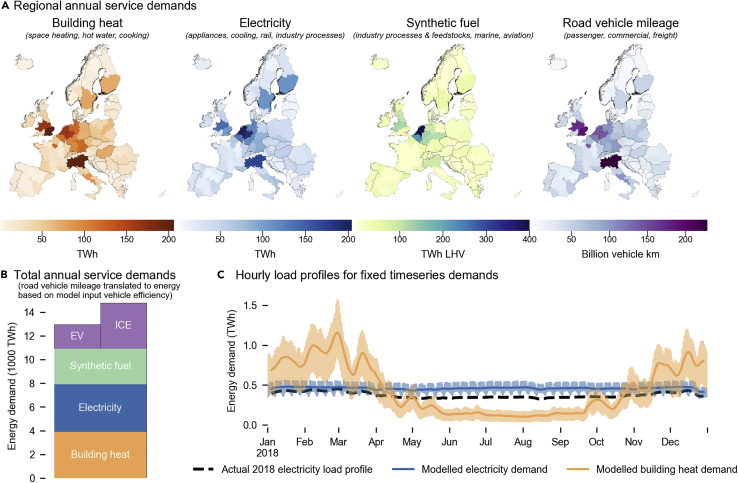
Modeled European energy demands and their spatiotemporal distribution Magnitudes and spatiotemporal distributions of end-use service demands for the year 2018, resulting from the data processing pipeline described in the experimental procedures and Note S1. Service demands for the year 2018 are used as an input to the baseline model runs to match the 2018 weather year used to define renewable technology capacity factor profiles. We also model demands for the years 2010–2017 with their respective weather data, which we use for sensitivity analyses (see Note S2). (A) Annual demand per sectoral group and modeled European region in 2018. “Building heat” refers to space heat, hot water, and cooking demand in residential, commercial, and industrial buildings. “Electricity” refers to all direct electrical end-use demand, based on historical electricity consumption minus electricity consumed to meet building heat and road transport demand plus additional demand from electrifying all rail and electrifying most industrial processes. “Synthetic fuel” refers to demand from industry for liquid and gaseous hydrocarbons as feedstock (e.g., methanol for chemicals) and for high temperature process heat (where it cannot be electrified), and for liquid fuel demands in domestic and international aviation and shipping. Fixed demand for hydrogen and CO_2_ in industry is assumed to be directly electrified and thus combined into “electricity” demand. Road vehicle mileage encompasses all road vehicles; rail has been assumed to be fully electrified and is combined into “electricity.” (B) Total annual energy demand for the whole system in 2018. Groupings are the same as given in (A), with transport demand converted from vehicle km to demand for energy based on the entire fleet being electrified (EV) or the entire fleet consuming liquid fuels in internal combustion engines (ICE). (C) Hourly (transparent area) and seven-day rolling average (solid line) demand of end-use electricity and building heat demand. Groupings are the same as given in (A). “Current electricity load” refers to 2018 electricity load across all modeled countries, according to ENTSO-E published statistics. For visual clarity, only the seven-day rolling average data are given for actual 2018 electricity load.

We start by analyzing energy demand when grouping all energy consumption into four services (Figure 1). For each, we model total demand and its spatiotemporal variability using a combination of statistical datasets and simulation results (see experimental procedures and Note S1). The four main energy services are (1) space, water, and cooking heat demands (“building heat”); (2) hydrocarbon demands in place of fossil fuels for non-electrifiable industry processes or feedstocks as well as aviation and shipping (“synthetic fuel”); (3) the distance traveled by passenger, commercial, and freight vehicles on roads (“transport vehicle mileage”); and (4) electricity consumption by building-level appliances and cooling, passenger and freight rail, and industry processes (“electricity”). Most of these demands are based on 2018 levels (building heat, appliances, and cooling; transport distance; aviation and shipping fuels), whereas some are based on today’s demands following electrification using today’s technology efficiencies (rail and, where possible, industry processes) or a complete overhaul of processes to avoid reliance on fossil feedstocks (steel and chemical industries). Together, service demand in our model is 2.61–2.85 times (depending on the road transport technology choice) higher than electricity demand in 2018, highlighting the importance of our sector-coupled approach.

We explore how these demands can be met by a predominantly renewable energy system relying on proven and commercially available technologies as far as possible, in agreement with previous work^23^ that has shown how waiting for unproven technologies to become available could result in substantially higher transition costs. This means we assume that building heat and transport vehicle demands can be met directly by electricity, with biofuel- or electricity-derived hydrocarbons, or with direct use of biofuels or municipal waste (heat only). This illustrates two potential distributions of burden across society: (1) industry and utility-scale actors drive a new synthetic fuel generation industry, enabling consumers to continue meeting demand with hydrocarbon-reliant end-use technologies, and (2) consumers electrify their end-use technologies alongside the transformation of local supporting infrastructure, such as electric vehicle charging and reinforced local electricity distribution networks. To enable the transmission of energy between regions, we assume that high-voltage electricity grids and fuel transport infrastructure are available. Hydrogen can be used as a feedstock in producing synthetic fuels or for utility-scale stationary storage. However, we do not consider its direct use for road transport or building heat due to the need for an overhaul of transmission networks as well as end-use technologies to enable distributed hydrogen use^24^ and the emerging market dominance of electrification, for instance, in passenger and freight vehicles.^25^^,^^26^ Because of the limited availability of non-electrically derived carriers to satisfy most energy demands, namely biofuels and municipal waste, the total primary supply of electricity increases.

Today, demand is distributed unevenly across Europe (Figure 1A). For example, because of its large petrochemical industry, the Netherlands stands out as a large fuel consumer. The high population density of south-eastern United Kingdom, northern Italy, and north-western Germany concentrate electricity, heat, and transport demand. Building heat demand is not only spatially diverse, but its temperature-dependence leads to pronounced seasonal variability (Figure 1C). Because both demand and renewable energy supply vary in space and time, modeling the design of a continent-spanning renewable energy system for all energy-consuming sectors requires representing this variability with sufficient detail. Therefore, we build a linear optimization model with 98 nodes and a 2 h temporal resolution for 4,380 time steps over a full calendar year, with the objective to supply energy at lowest total cost. We enforce constraints to ensure that all demands are met while restrictions on the deployment of generation and transmission technologies are respected. We then systematically explore options close to the least-cost optimum, generating 441 technically and economically feasible, spatially-explicit practically optimal results (SPORES^21^). These SPORES represent feasible system designs in which all European demands for energy-consuming services can be met, based on 2018 magnitudes and spatiotemporal distributions, without any energy imports (for analyses concerning different weather years and a projected annual demand scenario, see Note S2). There are an infinite number of alternative configurations; our method, which is an extension of conventional MGA,^27^ specifically looks for those which expose the greatest diversity in technology choice and spatial configuration within 10% of the least-cost system.

## Diverse range of feasible designs for a self-sufficient, carbon-neutral European energy system

**Figure 2:**
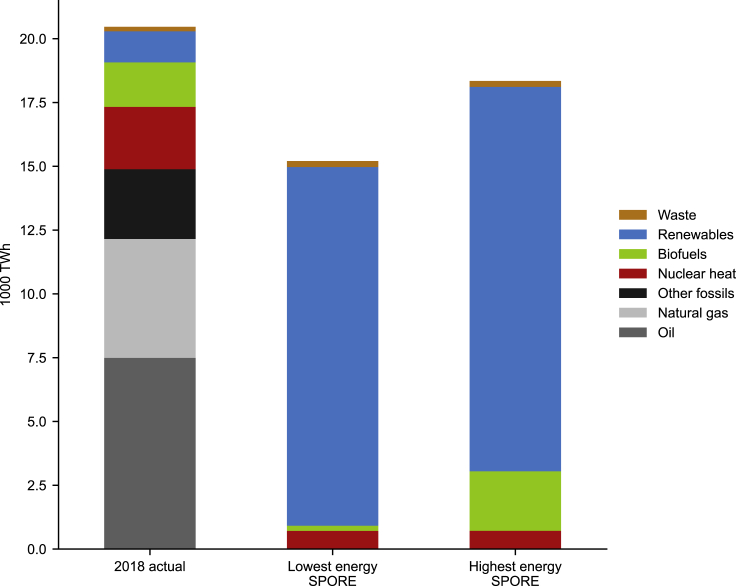
Current European gross available energy (GAE) compared with highest and lowest GAE in modeled energy self-sufficient, carbon-neutral Europe Gross available energy for 34 European countries. “2018 actual” refers to 2018 data from the Eurostat annual energy balances, category *GAE*, that includes primary production, recycled and recovered products, changes in stock, and net imports. For more detail on mapping of Eurostat energy carriers to technology groupings in this figure; see primary energy supply. “Lowest energy SPORE” refers to a feasible energy system within 10% of the cost-optimal solution with the lowest overall primary energy supply. “Highest energy SPORE” refers to a feasible energy system within 10% of the cost-optimal solution with the highest overall primary energy supply. Since the end-use service demands are fixed across SPORES, the differences in primary energy supply are caused by inefficiencies in intermediate processes, such as producing synthetic fuels from electricity-derived hydrogen. Primary energy supply in the model result includes electricity directly produced by renewables (wind, solar, and hydropower), municipal waste, residual biofuel, and nuclear heat. Modeled nuclear heat is calculated according to the Eurostat energy balances as nuclear power electricity production divided by average plant efficiency. All countries in the study area except Switzerland are included.

We find that many near-optimal energy system configurations based predominantly on solar and wind electricity can supply all European energy demand. Across all SPORES, electrification efficiency gains would decrease primary energy supply compared with today, as illustrated by the two options that meet demand with the highest and lowest primary energy supply in Figure 2: both are lower than primary energy supply today.

Since we do not allow energy imports into our model region, the supply in all SPORES is generated within Europe. This shows that it is feasible to eliminate net imports currently equivalent to almost half of primary energy supply (9,122 TWh in 2018, predominantly by import of fossil fuels). Fixed synthetic fuel demands for industry processes, and marine and aviation fuels, mean that primary energy supply does not reduce as much as might be expected between historical levels and those given by our SPORES. That is, efficiency gains from electrification are partly offset by inefficient processes to produce carbon-neutral fuels in all SPORES. Exactly how much these inefficient intermediate processes affect the total primary energy depends on the approach to carbon neutrality taken in each sector, since the options to meet energy service demands have different efficiencies. For example, less energy is required for the same total distance traveled by vehicles if these vehicles are fully electric than if they still use liquid fuels (Figure 1B). The more Europe relies on its current means of heating (especially methane) and road transport (oil), the more primary electricity supply is required to manufacture carbon-neutral fuels.

The extent to which sectors should be electrified is just one of many decisions that can be taken when designing a carbon-neutral, renewable European energy system. Our 441 different SPORES allow us to explore how extensive this decision space is and to quantify the trade-offs between preferences in detail.

## Maneuvering space exists between competing interests without compromising cost-effectiveness

**Figure 3:**
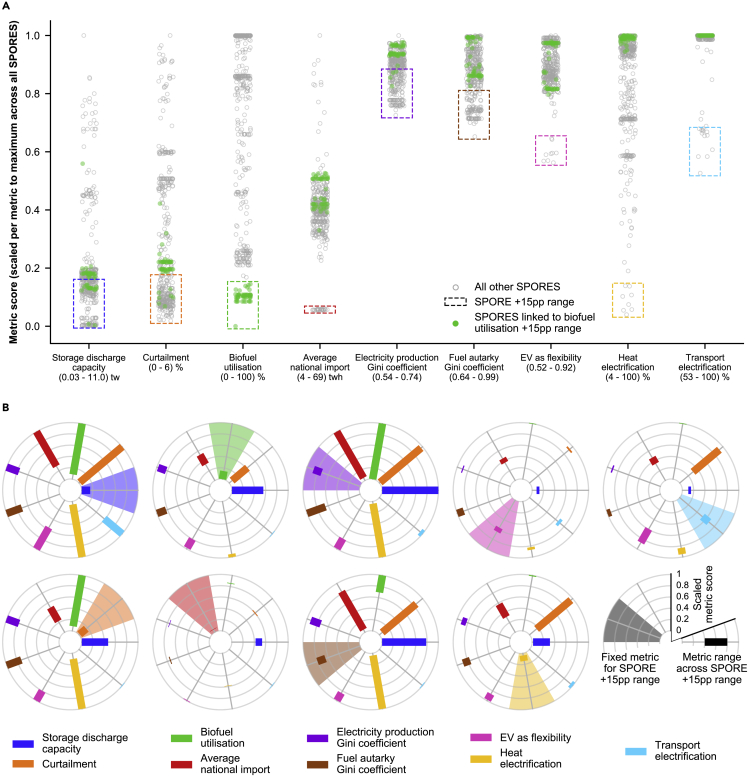
Near-optimal decision space described by 441 energy system configurations Decision space of 441 feasible energy system configurations (SPORES) within 10% of the cost-optimal total system cost, as shown through a selection of nine metrics that may be particularly relevant for decision-makers making energy system planning decisions. (A) Performance of SPORES, according to scores for the nine different metrics scaled to their maximum value in any SPORE. SPORE ranges do not describe a statistical distribution since our method is focused on exploring options around the periphery of the decision space (for more detail, see SPORES). The SPORES within 15 percentage points of the lowest score per metric after scaling (the “+15pp range”) are highlighted by colored boxes. All SPORES connected with the +15pp range of biofuel utilization are colored green, which we use to define the metric ranges depicted in (B). See Note S4 for SPORES highlighted according to the +15pp range of other metrics. Horizontal placement of SPORE markers in each metric is random to better view the spread of data points that would otherwise overlap, using the “jitter” functionality provided by the Python package *Seaborn*. (B) Scaled range that the other eight metrics can take (shown as bars) when one metric is fixed to within the +15pp range highlighted in (A). Each fixed metric is shown as a bar with a highlighted background. Each radial chart holds one metric to within the +15pp range and therefore highlights how strongly the decision space is constrained if a low value for that metric is of particular importance. Colors of metric bars and background highlights in (B) match the colors of the boxes surrounding the +15pp range of SPORES in (A).

We first choose a set of metrics that quantify aspects that have received particular attention in energy policy debates, such as the uncertain role of energy storage in highly renewable energy systems^28^, ^29^, ^30^, ^31^ or the extent to which countries are electricity autarkic^32^ (Table 1). We formulate the metrics such that lower values are more preferable in a broad sense: more equally distributed infrastructure, less electrification (i.e., less consumer-level change), or less use of possibly problematic or controversial technologies like energy storage or biofuels. Figure 3A illustrates the range of each metric across all SPORES after scaling the metric relative to its highest value in any SPORE. Sub-selections of SPORES within 15 percentage points of the lowest value for each scaled metric are highlighted in colored boxes (the “+15pp range”). We see that for some metrics, such as biofuel utilization, there are solutions across the entire range. This means that there are near-optimal solutions that use all of the available biofuel potential and others that use next to none of it. For other metrics, most of the possible energy system configurations are within a narrow band; e.g., road transport does not go below 53% electrified in any SPORE.

**Table 1:** Definition of high-level energy system metrics and their range across the decision space

Metric name	Metric description	Metric range
*Storage discharge capacity*	total capacity of all storage technologies to discharge energy in any given hour, including low-temperature heat, hydrogen, and electricity	0.03–11 TW
*Curtailment*	percentage of maximum available renewable electricity production from wind and solar photovoltaic technologies that is curtailed	0%–6%
*Biofuel utilization*	percentage of available residual biofuels that are consumed	0%–100%
*Average national import*	average annual import of electricity across all countries within the study area	4–69 TWh
*Electricity production Gini coefficient*	degree of inequality of spatial distribution of electricity across all model regions, measured by the Gini coefficient of regional electricity production	0.54–0.74
*Fuel autarky Gini coefficient*	degree of inequality of spatial distribution of industry synthetic fuel production relative to industry fuel demand across all model regions, measured by the Gini coefficient of regional overproduction	0.64–0.99
*EV as flexibility*	Pearson correlation between timeseries of electric vehicle charging and that of primary electricity supply	0.52–0.92
*Heat electrification*	percentage of heat demand met by electricity-consuming, heat-producing technologies	4%–100%
*Transport electrification*	percentage of road passenger and freight transport demand met by electric vehicles	53%–100%

**Figure 4:**
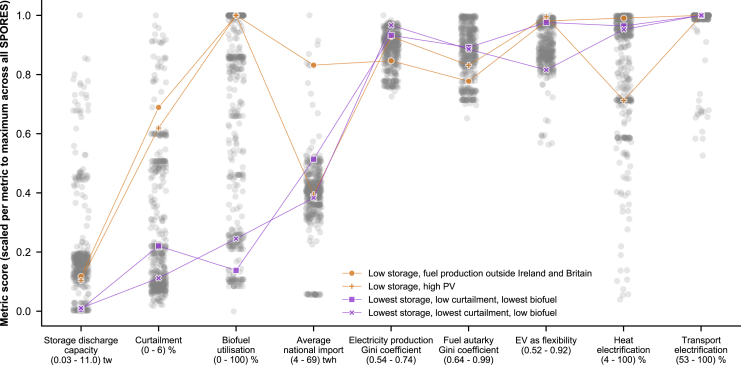
Position of four example energy system configurations in the near-optimal decision space Four example SPORES selected from the total number of 441 to cover both low and medium deployment of storage discharge capacity and different allowed degrees of curtailment, biofuel consumption, and spatial distribution of generation and synthetic fuel production. Connecting lines between markers do not imply data interpolation between metrics but are to visually aid the trade-offs between metrics for a specific SPORE. These examples illustrate the synergies and trade-offs that open up between competing goals across all predefined metrics (see Table 1) but should not be considered the only trade-offs that can be analyzed from our results. Readers are encouraged to use these examples as a guide to explore further trade-offs themselves, with our interactive data explorer: https://explore.callio.pe. Highlighted in purple are the two SPORES selected based on keeping storage discharge capacity below 0.1 of its scaled score (“lowest storage”) and one of curtailment or biofuel utilization below 0.3 (“low”), while the other is minimized (“lowest”). Highlighted in orange are the two SPORES selected based on keeping storage discharge capacity below 0.2 (“medium storage”) while defining preferences outside the scope of the metrics. One is chosen to maximize total PV deployment in Europe (“high PV”); the other is chosen by filtering SPORES to those in which hydrogen production in Britain and Ireland is below 10% of total European hydrogen production (“fuel production outside Ireland and Britain”). Overlaps between SPORES on any metric are purely coincidental, showing how trade-offs are not immediately obvious from the primary selection criteria of a SPORE. Metric values across all SPORES (gray circles) do not describe a statistical distribution, since our method is focused on exploring options around the periphery of the decision space (for more detail, see SPORES). Horizontal placement of SPORE markers in each metric is random to better view the spread of data points that would otherwise overlap, using the “jitter” functionality provided by the Python package *Seaborn*.

Definition of high-level metrics that describe energy systems that may be particularly relevant to specific stakeholders or interest groups, and the range of values of each metric across all SPORES results. The metric values across all SPORES are shown scaled relative to their maximum values in Figures 3 and 4.

We can investigate how severely the decision space is constrained if we are concerned about one particular metric, for example, the use of energy storage (utility-scale batteries, hydrogen tank storage, and low-temperature heat storage). In Figure 3B, we plot the range that each of the remaining metrics can take when one metric is held to within its +15pp range. We see that even when constraining storage discharge capacity, there is still maneuvering space to choose the degree to which we wish our energy system to depend on biofuels, expansion of the electricity transmission network, or the electrification of building heat. Similarly, electricity and synthetic fuel production can be distributed more evenly across Europe (lower gini coefficients) while retaining maneuvering space in most other metrics. However, this distribution does require vehicle electrification rates to be close to 100%.

Constraining biofuel utilization to its +15pp range dramatically reduces the decision space remaining on most other metrics. Given the technological options we consider, it implies a high degree of heat electrification and of using electric vehicles as a flexibility source in the power system. The maneuvering space is even more restricted when electricity transmission network utilization is kept low, implying effectively fixed metric values in all other SPORES.

Even with more constrained sets of solutions, maneuvering space still exists. However, the more we wish to maintain specific preferences, the more the remaining maneuvering space is reduced. It is not possible to keep all metrics within their lower bound: at most, four metrics can be within +15pp of their lowest scaled values before trade-offs have to be made.

Furthermore, there are many relevant dimensions beyond the nine metrics we start our analysis with. For example, we find that many resulting configurations place a large amount of synthetic fuel production in Britain and Ireland, exploiting the particularly high wind power potential in that area. We also find that many system configurations rely on wind more than on PV.

To explore these dimensions and the effects of trade-offs across multiple high-level metrics, we select four example SPORES for more in-depth analysis. We choose two SPORES on trade-offs from the nine metrics: both keep storage use, curtailment, and biofuel use low, with one prioritizing the minimization of curtailment and the other of biofuel use. The other two SPORES we choose are aimed at exploring the aforementioned dimensions of the system beyond our chosen metrics: both keep storage use low, with one having PV capacity in the 90th percentile of capacities across SPORES and the other having fuel production within Britain and Ireland below 10%. We highlight the four resulting SPORES in Figure 4. Since there are an almost infinite number of reasons to select SPORES for more detailed analysis and comparison, we use our four selected SPORES as an example of how to hone in on features of interest in the option space. Other researchers and decision-makers will want to focus on other aspects from the many dimensions which our set of results spans, which they can do using our interactive data explorer: https://explore.callio.pe.

We see that in some instances, two different rationales for imposing preferences can lead to similar impacts on metrics that have not been considered. High PV capacity and moving fuel production outside Britain and Ireland lead to the selection of SPORES with a similar degree of technology curtailment and the same biofuel utilization. However, that is where the similarities end; there is markedly different dependence on transmission and heat electrification of these two configurations. Infrastructure planning is a key aspect of the energy transition and one key aspect of our method is to explore the spatial diversity of infrastructure deployment. Indeed, a criterion like “low use of storage” can have a wide variety of regional effects. To explore this, we turn to examining the spatial dimension of our four selected example SPORES.

## Different spatial configurations can satisfy high-level constraints equally well

**Figure 5:**
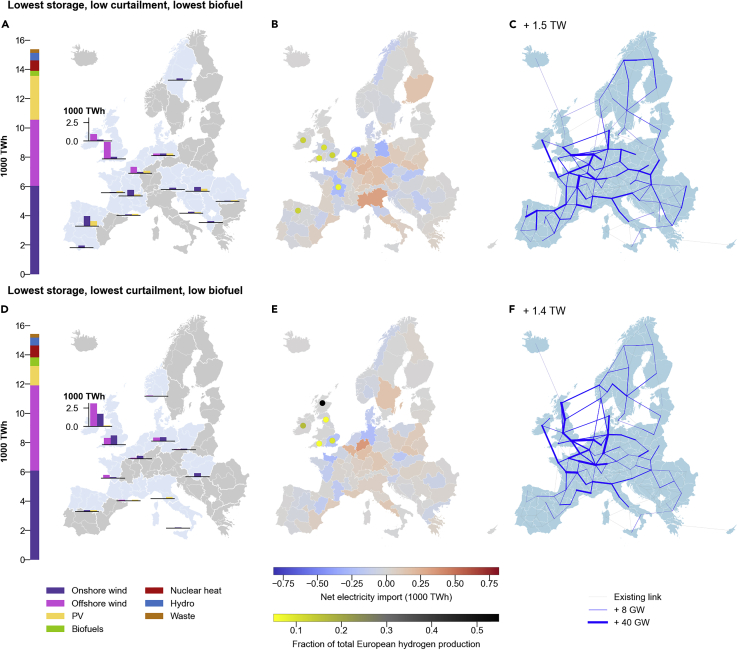
Spatial distribution of energy generation and total primary energy supply of two example energy system configurations in the near-optimal decision space Spatial distributions and total primary energy supply for two of the four selected SPORES defined in Figure 4; “lowest storage, low curtailment lowest biofuel” (top) and “lowest storage, lowest curtailment, low biofuel” (bottom). Readers are encouraged to use these examples as a guide to explore further trade-offs themselves, with our interactive data explorer: https://explore.callio.pe. (A and D) Spatial distribution of onshore wind, offshore wind, and PV supply (right) and total primary energy supply across all regions (left). Capacities are shown for 29 zones that are aggregated from the 98 model regions to give comparable land area. Zonal supply is only shown when the sum of supply in that zone is greater than 6% of maximum supply from one technology in any region, making it visually easier to see the major supply hubs. All zones for which supply is not shown constitute 8%–11% of total European supply. Cyprus is not shown, but these data are included in the same zone as Greece in the maps. Biofuels, waste, hydro, and nuclear electricity supply are not shown on the maps. (B and E) Annual regional net electricity import and high synthetic fuel-producing regions. Data are shown at the resolution of the 98 model regions. For each region, annual net electricity import is the sum of all electricity imported from connected regions over the year minus electricity exported to connected regions over the year. A positive net import indicates a region imports more electricity than it exports, while a negative net imports indicates more exports than imports. High synthetic fuel-producing regions are those producing above 5% of European total hydrogen. Since hydrogen cannot be transported between regions in our models or directly consumed to meet service demands, high hydrogen production is equivalent to high synthetic fuel production. (C and F) Electricity grid transmission expansion beyond existing or planned capacities between regions. In the top-left of each panel is the total line capacity added across Europe above the baseline capacities for that feasible configuration. Light gray lines depict regions connected by transmission lines that are not expanded. Where transmission expansion occurs, lines are shown in blue, with increasing thickness indicating increasing transmission expansion. Two values in the legend mapping expansion to line thickness refer to the mean and maximum expansion of any one line of all those that are expanded.

We now compare our four selected example SPORES with respect to wind farm and PV deployment, net electricity imports and contributions to carbon-neutral fuel production, and expansion of the electricity transmission grid. Maintaining low reliance on storage, biofuels, and curtailment tends to require capitalizing on the high wind power productivity around Britain and Ireland to create hydrogen production hubs. However, capacity deployment can still vary greatly (Figures 5A and 5D). For instance, wind capacity can be split differently between Ireland and Great Britain and between onshore and offshore wind farms. In addition, large hydrogen production facilities could either be entirely concentrated in Britain and Ireland (Figure 5E) or include hotspots in Spain, France, and the Netherlands (Figure 5B). Strong reinforcement of transmission lines between northern European regions would occur in both cases (Figures 5C and 5F), but further reinforcements toward the Iberian peninsula would be required in the case in which generation capacity is more distributed (Figure 5C).

**Figure 6:**
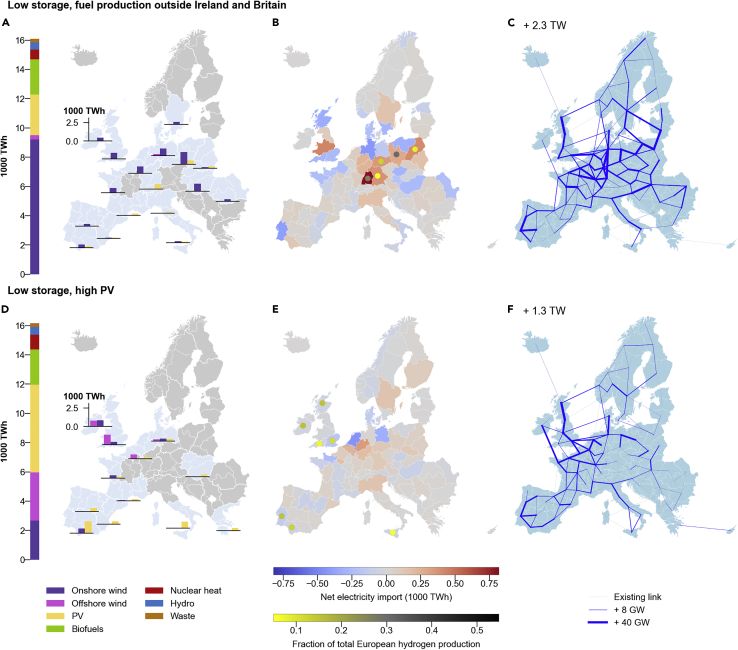
Spatial distribution of energy generation and total primary energy supply of two example energy system configurations in the near-optimal decision space Spatial distributions and total primary energy supply for two of the four selected SPORES defined in Figure 4; “low storage, fuel production outside Ireland and Britain” (top) and “low storage, high PV” (bottom). For a full description of each panel, refer to the caption of Figure 5, which shows the same information for two other SPORES

When we slightly relax our restrictions on high-level metrics, we can see that radically different system configurations are possible. For instance, it is possible to move fuel production outside Britain and Ireland to eastern European countries (Figure 6A). In this deployment strategy, such countries would become net electricity importers and key hubs for the production of hydrogen (Figure 6B). This would be made possible by marked expansion of transmission lines throughout the continent (Figure 6C) and by full utilization of residual biofuels. Alternatively, similar relaxations on storage deployment and biofuel use could allow a much larger deployment of solar capacity in southern Europe, combined with a substantially lower deployment of wind overall (Figure 6D). This would enable splitting hydrogen production facilities into southern and northern hubs (Figure 6E) and would come with lower requirements in terms of infrastructural change, such as a moderate electrification of the heat sector and a limited expansion of transmission lines (Figure 6F).

These are just two examples of how a high-level goal can be met by very different system configurations. In both cases, many more options can be drawn from our full set of results. We can thus conclude that there is considerable maneuvering space for infrastructure siting, even when we want certain high-level preferences to be fulfilled and that this maneuvering space expands even more when we are willing to compromise on some metrics, such as allowing more biofuel use.

## Discussion

Our analysis leads to three important implications for the implementation of a self-sufficient, carbon-neutral European energy system. These results are relevant both to guide the development of detailed pathways in future academic work and for policy makers to decide on priorities when translating decarbonization targets into more concrete plans. First, we find that there is a diversity of options to design a self-sufficient, carbon-neutral energy system relying as much as possible on technologies that are already available. This requires a large expansion of wind and solar power together with electrification and synthetic fuels. This finding is strengthened by our high spatiotemporal detail on both energy demand and renewable supply, which enables our model to depict both the drawbacks of renewable variability and the benefits of sector coupling to deal with this variability.

Second, we find that there are few must haves: a system can be designed to rely to a varying extent on wind energy, PV, biofuels, intra-European transmission, storage, electrification of heat and transport, or use of controlled vehicle charging. We also find that “firm generation” is not strictly necessary. This result differs from many recent studies that expect a reasonable proportion of demand to be met by some form of firm capacity, be it through biofuels,^11^ nuclear,^33^ or fossil generation with measures to offset or capture emissions.^19^^,^^34^^,^^35^ Our portfolio of configurations also encompasses recent studies that do not use firm capacity. Bogdanov et al.^5^ present wind (32%) and PV (62%) as the two primary electricity sources in a carbon-neutral Europe. Pleßmann and Blechinger^36^ agree on the extent of renewables, but with an opposite ratio of 63% from wind and 20% from PV; in other studies, wind and PV ratios sit somewhere in between.^12^^,^^37^^,^^38^ We show that all of these studies are correct, in that all of these solutions are possible. When considering the impact of PV-to-wind ratio on seasonal balancing of variability, we find that there is substantial flexibility on what solutions to deploy to address this problem, although generally in high solar production years, summer overproduction is dealt with by more electrolysis and winter underproduction dealt with by combined-cycle gas turbines (CCGTs) and hydropower (see Note S3). By modeling an entire decision space rather than a single solution, we can quantify the trade-offs between choices, supporting decision-makers in their deliberations.

Third, there is a great deal of flexibility in where to locate infrastructure in Europe. We show that specific regions, like Britain and Ireland or eastern Europe, can be prioritized for electricity or fuel production. As regional equity becomes increasingly important in the transition discourse, an understanding of this regional maneuvering space can support discussions on benefits and drawbacks of new infrastructure. For example, regions could capitalize on becoming synthetic fuel production hubs. Our modeling approach allows decision-makers to examine different spatial configurations, which can inform incentive schemes to foster such regional economic development. In addition, we show it is possible to eliminate the need for fossil fuel imports from outside of Europe. The resulting economic benefits captured within Europe itself could be considerable, while simultaneously ensuring security of supply.

Our sensitivity analyses confirm the robustness of these conclusions, with respect to the allowable cost relaxation and the choice of weather year (and thus variability of renewable generation and demand). In addition, although we do not project future energy service demands for our main results, given the uncertainty associated with them, our sensitivity analysis shows that our decision space remains qualitatively the same if we use simulated changes in annual demand to 2050^39^^,^^40^ (see Note S2). Nevertheless, there are several limitations in our work. The true robustness of system designs to a range of weather conditions expected to occur over the decades of investment lifetime needs more work bringing together climate science, meteorology, and energy engineering.^41^ Furthermore, in our model, the technological and spatial maneuvering space is enabled by a willingness to have a system up to 10% more expensive than the cost-minimal continent-wide system configuration. The option space diminishes if we restrict this willingness to pay and increases with a greater willingness (see experimental procedures and Figure S17). However, for a model of all energy-using sectors, “total system cost” is difficult to interpret, given that there is no single actor to which these costs accrue; therefore, we refrain from making statements about the cost of the transformation. Still, it is possible to examine the designs from the perspective of the burden carried by different groups of actors in different regions—for example, consumers (rooftop PV, car purchases, and heating technology choice), utilities (power plants or grid expansion), and industries (process changes and synthetic fuel generation). This offers a basis for future work to focus on.

It is also important not to interpret our results as a forecast or prediction of the future. Instead, they are a systematic exploration of a fully linearized representation of the European energy system design space under the broad assumptions we outline above and in Note S1, like the large-scale reliance on variable renewable generation. Further work is required to broaden the scope of available technological solutions and to analyze the impact of nonlinear processes, such as transmission grid power flow. However, a model like ours can only be run on specialized high-performance computers due to its complexity. Therefore, a practical approach to conducting further work is to first constrain the option space based on features of interest and then generate near-optimal solutions with updated assumptions in this constrained space. That is, we recommend expending effort only on the parts of the design space that are of interest to decision-makers. This approach would also be necessary to examine carbon-neutral system designs from perspectives that we do not consider in this study, such as macro-economic impacts, local infrastructure effects, and feasible transition pathways. Indeed, investigating possible lock-in effects, including how the option space becomes increasingly constrained the longer we wait to make decisions, is an urgent problem that requires more attention in future work.

We show a broad variety of ways to build a carbon-neutral energy system that meets all European energy demands. However, to avoid catastrophic climate change, the scale of the transformation and the speed at which it must proceed remain enormous. For instance, if we assume that the deployment of renewables in European follows an “S-curve” to 2050,^42^ then the maximum growth requirements of combined wind and PV in Europe would be 681–932 TWh/year across our option space. This requires a ten-fold increase in annual growth by the early 2030s, when compared with Europe’s maximum historical annual growth of 66 TWh/year. For this reason, we focus on technologies that are ready to scale up or are already in the process of scaling up rapidly, with two exceptions: some industry processes in the steel and chemicals subsectors and the formation of a synthetic fuel industry that can manufacture liquid and gaseous fuels from electricity and biofuels. Irrespective of the exact system configuration, large-scale infrastructure deployment is necessary. The solutions we show will require deployment of renewable energy at an unprecedented scale and will affect all industry subsectors and all individuals in their homes, vehicles, and workplaces.

The maneuvering space we identify decreases substantially as soon as we wish certain preferences to be fulfilled: for example, if we wish to completely forego the use of biofuels or energy storage. Understanding such trade-offs—and the implications they have for how quickly the continent can reach carbon neutrality—is important for decision-makers and for society as a whole. This requires bringing stakeholder views into the techno-economic modeling process and reflecting the real decision space back to stakeholders.^43^ Ultimately, the extent to which our model is useful depends on whether it captures real-world trade-offs; as academics, we are not in the best position to make this judgment. For this reason, we make all results available to actual decision-makers through our web interface (https://explore.callio.pe) and encourage further work to explicitly bring real-world decision-makers into the loop of modeling exercises. By using an approach like ours to guide and structure the process on narrowing down the economically, socially, and politically acceptable design space for the target system, follow-up analyses can investigate specific technical aspects—such as grid operation—in more detail and can examine possible pathways and supporting policy mechanisms to reach the target design. Our analysis of the trade-offs within the designs we select above can be seen as a guide for others to explore the myriad additional trade-offs further, using our models, data, and interactive interface.

## Experimental procedures

### Resource availability

#### Lead contact

Further information and requests for resources and materials should be directed to and will be fulfilled by the lead contact, Bryn Pickering (bryn.pickering@usys.ethz.ch).

#### Materials availability

The model data processing workflow generated in this study has been deposited to GitHub: https://github.com/calliope-project/sector-coupled-euro-calliope. The final model using the baseline 2018 data, ready for use in Calliope, has been deposited to Zenodo: https://doi.org/10.5281/zenodo.5774988. All model results, for all baseline SPORES and sensitivity runs, have been deposited to Zenodo: https://doi.org/10.5281/zenodo.6546817.

### European energy system model setup

The European energy system model is an expansion of the stylized power system model Euro-Calliope v1.0.^1^ Our sector-coupled Euro-Calliope model takes the current configuration of *all* European energy consumption as a departure point to model credible future configurations in a realistic manner. Compared to the single energy carrier considered in power system models, we represent 13 carriers in our sector-coupled Euro-Calliope: electricity, hydrogen, CO_2_, liquid and gaseous hydrocarbons (kerosene, methanol, diesel, and methane), solids (residual biofuel and municipal waste), low-temperature heat (combined space heat and hot water, and cooking heat), and vehicle distance (heavy- and light-duty road vehicles). These carriers can be consumed, produced, and converted by a variety of technologies to meet demand. In addition, low-temperature heat, hydrogen, electricity, and methane can be stored. Since future international energy commodity prices are highly uncertain, energy imports from outside our model region are not allowed. Accordingly, all our model results represent system designs in an energy self-sufficient Europe. We describe the key components of input data processing in the following subsections, with an overview of primary data sources given in Table 2. Further details on the model setup and data processing are provided in Note S1, and the full representation of carrier and technology connections is given in Figure S13.

**Table 2:** Summary of primary sources used in data processing pipeline

Model component	Temporal processing		Spatial processing	
	Annual	Hourly	National	Sub-national
Electrified rail	Eurostat & JRC-IDEES	DESSTINEE	Eurostat & JRC-IDEES	population & industry density
Road transport	Eurostat & JRC-IDEES	RAMP-mobility	Eurostat & JRC-IDEES	population & industry density
Aviation	Eurostat	–	Eurostat	industry density
Shipping	Eurostat	–	Eurostat	industry density
Existing industry processes	Eurostat & JRC-IDEES	–	Eurostat & JRC-IDEES	industry density
New industry processes	Eurostat, JRC-IDEES, & literature	–	Eurostat & JRC-IDEES	industry density
Buildings: cooking	Eurostat & JRC-IDEES	RAMP-cooking	Eurostat & JRC-IDEES	population
Buildings: heat and hot water	Eurostat & JRC-IDEES	MERRA-2 & When2Heat	Eurostat & JRC-IDEES	population
Buildings: appliances and cooling	–	OPSD	OPSD	population
Viable nuclear regions	–	–	–	JRC powerplant database
Biofuel supply capacity	JRC ENSPRESO	–	JRC ENSPRESO	land use categorization
Municipal waste supply capacity	Eurostat	–	Eurostat	JRC powerplant database
Heat pump performance	–	MERRA-2 & WAKAM technology database	–	population & MERRA-2
Gas cavern storage capacity	–	–	GIE	equal distribution
PV and wind capacity factors	–	renewables.ninja	–	renewables.ninja & land use
PV and wind capacity limits	–	–	–	land use categorization
Hydro capacity factors	IRENA	MERRA-2 & atlite	IRENA	JRC Hydro database v7
Hydro capacity	–	–	JRC Hydro database v7	JRC Hydro database v7

Summary of primary sources used to process spatiotemporal demand and supply data to use as inputs to the sector-coupled Euro-Calliope model

The model is optimized as a linear programming problem at two-hour resolution over a whole year using the Calliope energy system modeling framework.^44^ The base year of the study, for weather and demand data, is 2018. We choose this year as we have the most complete statistical datasets available, thus requiring the least amount of gap-filling. For instance, prior to 2016/7, Albania and Bosnia and Herzegovina have limited data availability from Eurostat and ENTSO-E. See sensitivity analyses for information on the additional years used as sensitivity analyses. The full model workflow, including references to all data sources and the processing steps to generate the model, is freely and openly available online (see resource availability).

### Demand data

We source annual demand data from the Eurostat,^45^ JRC-IDEES,^46^ and Open Power System Data^47^ databases. We do not make any assumptions on changes in demand for services, such that our model is looking at a feasible, carbon-neutral configurations that would work with demand as we know it today. We do this for two reasons. First, the demand for services in the future is highly uncertain, with assumptions varying depending on modeling group and scenario.^19^ To take a specific example of this problem, the EU reference scenario 2020 assumes a 26% increase in distance traveled,^48^ while Bogdanov et al.^5^ assume an 80% increase. Second, our focus is on the features of system design exhibited when modeling energy service demands resolved in space and time. Because of this high resolution, we are able to model the potential for flexibility and sectoral coupling in the design of the energy system. This could make things easier, for example by balancing variable renewable generation with flexible charging of electrified transport. It could also make things harder, for example by adding additional pressure on the transmission system because of electrification of processes. It is therefore important to have synchronized energy demand and weather profiles, both in time and space. Without synchronicity, we risk missing the effect of sub-daily to seasonal meteorological phenomena that influence variable renewable supply as well as demand, both for heat in buildings and for electricity.^49^^,^^50^ Data from recent years are inherently synchronized; applying demand assumptions might lead us to unknowingly break this synchronicity. Although we do not attempt to project demand, the increase in final energy consumption given by our SPORE results (2018 to 2050: +(73%–93%) is in line with that given by the EU reference scenario 2020 (2015 to 2050: +84%), and remains in line when analyzing individual subsectors (industry, buildings, and land transport). Therefore, although we do not assume increases in service demands or decreases in demand intensity due to efficiency improvements, our resulting system representation is not inconsistent with those studies which do make such assumptions. Nevertheless, to assess whether changes in demand assumptions could make an impact on our modeling results, we have generated a subset of SPORES with scaled demands (see Note S2). These resulting SPORES suggest that the conclusions we draw from our study on the extent of the near-optimal option space is not affected by our use of 2018 service demands.

We group building heat demand into three end-uses: space heat, hot water, and cooking. These groups match the Eurostat database household end-use categorization, national data for which became available in 2020 (dataset: *nrg_d_hhq*). Commercial and Industrial sector building heat demands are not available on Eurostat, so we use JRC-IDEES, which has data for the period 2010–2015. We transform fuel consumption to a demand for heat by assuming technology efficiencies of heating technologies including boilers and direct electric heaters (see Table S2). These efficiencies are consistent with those used in our model for the available heat supply technologies. We use annual water and space heat demands to scale normalized hourly demand profiles produced using the methods implemented for the When2Heat database,^51^ updated to account for (1) all countries in our model scope and (2) the sub-national distribution of single- to multi-family homes across Europe, according to the Eurostat database of dwellings (dataset: *cens_11dwob_r3*). To generate cooking heat demand profiles, we extend the open-source RAMP engine^52^^,^^53^ to stochastically model demand in all European countries from the bottom up.

The transport sector encompasses road, rail, air, and shipping. We assume electrification is only possible in some of these forms of transport, namely road and rail. In rail, we assume complete electrification, taking current consumption of fuel for rail from the Eurostat annual energy balances (dataset: *nrg_bal_c*) and converting it to electricity demand using the efficiency of different rail drivetrains from JRC-IDEES. For airplanes and shipping, we take domestic and international fuel demands directly from the Eurostat annual energy balances and require them to be met by synthesis from hydrogen or from biofuels. Unlike for the other modes, we do not assume a “winning” drive train for road transport. Instead, we calculate the distance traveled by all vehicles in each country and use this distance as the road transport demand in the model. Annual vehicle mileage is based on JRC-IDEES and is split into motorcycles, passenger cars, buses, light-duty commercial vehicles, and heavy-duty freight vehicles. Vehicle mileage is then transformed back to energy demand based on the efficiency of different drivetrains. We use the 25th percentile of all countries’ vehicle energy consumption, as given by JRC-IDEES for the year 2015 to define vehicle efficiency. This represents a convergence on higher efficiency of vehicles in all countries in Europe, but not an improvement in countries with already efficient vehicle fleets.

Only light-duty (including passenger and commercial) electric vehicle and passenger rail demands are assumed to have hourly profiles impacting energy delivery; all other demands, which are for liquid fuels, must be met on an annual basis. Rail electricity profiles are taken from th Demand for Energy Services, Supply and Transmission in EuropE (DESSTINEE) demand model.^39^ Electric vehicles are limited in the allowed energy delivery per hour based on the number of vehicles connected to the grid at any given time. We generate this plug-in profile using RAMP-Mobility,^54^ an extension of the open-source RAMP engine mentioned above.^52^ The available charge capacity of plugged-in vehicles is based on the number of vehicles and an average battery size.^55^ This method allows the model to decide when to charge cars (smart charging), but ensures that it is not unrealistic in the frequency of charging throughout the year. That is, it cannot choose to charge all vehicles in one week of the year. In addition, we enforce that any electric vehicle demand must be balanced on a monthly basis, using demands derived from RAMP-Mobility.

We generate industry sector demands by considering each industry subsector separately. We assume most process heat can be met electrically^56^ and use JRC-IDEES electrical efficiency for meeting these demands to convert process demands to demand for electricity. Where JRC-IDEES has no electrical alternative for a process, such as for some steam processes, we retain methane demands in our model. We also mitigate the consumption of fossil fuels as feedstock to industrial processes in the iron & steel and chemicals subsectors, since these feedstocks contribute to a large proportion of these subsectors’ emissions.^56^^,^^57^ In iron & steel, we replace the conventional route of production (blast/basic oxygen furnace) with a completely electrified process: hydrogen-fueled direct reduction of iron followed by electric arc furnaces. To produce “high value chemicals” for plastics, we assume a feedstock of methanol to replace fossil fuels,^58^ which can be synthesized from hydrogen or from biofuels. In addition, we replace natural gas as a feedstock for ammonia and urea. To change processes in iron & steel and chemicals industries, we use demands for final products (steel, high value chemicals, etc.) from JRC-IDEES and calculate demands for hydrogen, CO_2_, and direct electricity based on estimated process efficiencies.

The final energy-consuming sectors given by the Eurostat annual energy balances not covered by any of the previous subsections are agriculture & forestry, fishing, and “not elsewhere specified.” These sectors account for approximately 2.5% of total European annual energy demand. We assume all oil consumption is for transport and add it to annual demand for heavy-duty vehicles (“agriculture & forestry” and non-kerosene use in “not elsewhere specified”), shipping (“fishing”), and aviation (kerosene in “not elsewhere specified”). All other non-electricity consumption is assumed to be for building heating applications, and therefore added to annual commercial building heat consumption.

Data gaps exist in demand, from both Eurostat and JRC-IDEES databases. In particular, JRC-IDEES does not extend beyond 2015 and only includes the EU28. Furthermore, Eurostat has limited or no data for some Balkan countries, Switzerland, and Iceland. Gap-filling is undertaken first by blending the JRC-IDEES and Eurostat dataset (e.g., demand per unit consumption from JRC-IDEES is applied to Eurostat energy consumption data in the years 2016–2018). If no data exist, data are interpolated in time and are based on neighboring countries in space. For Iceland, other Nordic countries act as the basis for data. For Switzerland, Germany, Austria, France, and Italy are the basis, although we also use specific data from Swiss government statistics. Similarly, for Balkan countries, direct neighbors are used. In all instances, demand *intensities* are used, not absolute demand. These intensities are then scaled based on country-specific data that are available, e.g., population, gross value added (GVA), and demand in other years. All of the resulting assumptions and data are freely accessible in the repositories linked to above.

### Supply data

Hourly wind farm and PV capacity factors are based on bias-corrected simulations using MERRA-2.^59^^,^^60^ We set upper limits on wind and PV capacities based on physical limits set by existing land use and infrastructure, following the bottom-up method described in Tröndle et al.^32^ Hourly hydropower capacity factors are based on ERA-5 runoff data, scaled to annual production of hydropower in each country. We assume hydropower capacities to be fixed, since expected future growth in Europe is limited.^61^ These capacities, for dams, run-of-river, and pumped hydro, and their regional distributions are all taken from version 7 of the JRC hydropower database.^62^ The available municipal waste supply is based on today’s consumption of municipal waste for energy, as defined by Eurostat. We do not assume any changes in municipal waste supply up to 2050. Nuclear capacity is limited according to possible ranges of future capacities from various sources. In most countries, this leads to no capacity, but in France and Finland, there is the opportunity for greater nuclear capacity than today. The nuclear capacity factor is limited to the range 75%–85% over the entire year, based on the capacity factor of the French nuclear fleet in 2018 and the worldwide median energy availability factor of nuclear reactors in 2006.^63^ Biofuel supply is based on projected 2050 residual biofuel availability (i.e., those leftover from existing agricultural and forestry processes rather than those specifically cultivated for the energy sector) given by the “medium” availability scenario in Ruiz et al.^64^ Hourly heat pump coefficients of performance (COPs) are based on gridded MERRA-2 air and ground temperature data scaled according to the average performance of new heat pumps sold by the manufacturer WAMAK,^65^ with a correction factor of 0.8 to scale for in-use performance.^51^ Gridded COP is then scaled to model regions using population and the proportion of ground-source (10%) and air-source (90%) heat pumps in the market today. To emulate the distributed nature of heat supply technologies, we introduce a constraint to ensure that the ratio of capacity investments is reflected in the share of each technology meeting demand in each hour. For instance, if 50% of heat supply capacity comes from heat pumps, they must also meet 50% (±2.5%) of heat demand in each hour. Synthetic fuels are an intermediate fuel to meet demand and can be derived from biofuels or electricity. The electricity route entails the generation of hydrogen by electrolysis and CO_2_ by direct air capture. Both energy sources and be used to produce any of the modeled hydrocarbon energy carriers: methane, kerosene, diesel, and methanol. Technology costs and all non-hourly characteristics are almost entirely sourced from the Danish Energy Agency technology catalog,^66^ using their 2050 projections, for internal consistency.

### Regionalization

The model represents 35 European countries: the EU-27 (minus Malta), Norway, Iceland, Switzerland, Bosnia and Herzegovina, Montenegro, North Macedonia, Serbia, Albania, and the United Kingdom. We have modeled larger countries by sub-national regions, based on those developed within the European Commission Seventh Framework Programme project e-HIGHWAY 2050.^67^ Sub-nationalization excludes Iceland, Ireland, Belgium, Luxembourg, the Netherlands, Estonia, Slovakia, Hungary, Slovenia, Croatia, Bosnia and Herzegovina, Montenegro, North Macedonia, Serbia, Albania, and Bulgaria. In total, there are 98 model regions. To regionalize sub-sectoral demands, different datasets have been used for different end-uses. Household and public and private passenger transport demand is regionalized using population. Commercial building and light-duty vehicle demand is regionalized using NUTS3 GVA from non-industrial subsectors (dataset: *nama_10r_3gva*, classifications G–U). Industry demand, including from freight transport, is regionalized depending on subsector. For industries with emitters registered in the EU emissions trading scheme (EU-ETS), we use the location and size of emitters in 2014 as a proxy for regional demand. For all other subsectors, we combine the number of employed individuals in each industry subsector (dataset: *sbs_r_nuts06_r2*) with quantity of loaded freight in each industry subsector (dataset: *road_go_na_rl3g*). We regionalize demand for aviation and shipping fuels based on average industry regionalization, on the assumption that these fuels would be synthetically generated in industrial regions, rather than exclusively at the point of consumption (e.g., major ports for shipping fuel).

Supply and storage capacities are regionalized for only a subset of technologies. Nuclear capacities can exist within a range, but the regions in which those capacities can be allocated is based on today’s concentration of regional capacities. Hydropower capacities are regionalization based on the JRC hydropower database v7. Wind and solar capacity regional upper bounds are based on a bottom-up process, combining high-resolution technical eligibility criteria described in Tröndle et al.^32^

### Spatial energy distribution

The initial high-voltage transmission network is based on the e-HIGHWAY 2050 project, in which a detailed analysis of the network was undertaken to produce simplified power capacities for each sub-region interconnection, as well as 48 planned/proposed new or upgraded connections described in the 2018 ENTSO-E ten year network development plan (TYNDP).^68^ The capacity of these connections act as a lower bound that can be further expanded. Modeling grid expansion purely linearly may underestimate the cost of grid expansion. However, we mitigated this by differentiating the cost of additional grid expansion based on the actual costs of planned and recently completed projects, differentiated by distance and terrain. Inter-regional fuel distribution is represented by grouping all industry synthetic fuel demands into European-level demands that can be contributed to by any model region. We do not model distribution networks within model regions, nor do we consider costs associated with them.

### SPORES

Thespatially explicit practically optimal results (SPORES) method by which we generate 441 equally feasible, near-optimal solutions is an advancement of the MGA method,^27^^,^^69^ which we introduced in previous work.^21^ Compared to other MGA approaches,^20^^,^^22^^,^^70^ SPORES is unique in making explicit the search for both technologically and spatially distinctive configurations of the energy system. Not only does the SPORES method look for equally feasible configurations in which, for instance, wind is deployed more than solar; it also explicitly looks for many feasible ways of spatially locating wind capacity at the sub-national scale, within roughly the same mix of deployed technologies. This proves particularly helpful to obtain configurations that may address regional equity and social acceptance concerns.^21^

The core of the SPORES approach, as applied in this work, is the following. First, we identify the cost-optimal solution as a starting point. Second, we assign an integer weight to every non-zero regional realization of technology capacity deployment in the cost-optimal solution, e.g., for wind deployed in Scotland. Third, we modify the model formulation such that the objective becomes the minimization of the sum of these integer weights. This means, in practice, that we push the model to avoid the deployment of those technology-region combinations, such as “wind in Scotland,” which have previously been part of a feasible solution. Finally, we implement total annualized system cost as a global constraint, such that feasible solutions with different technology-region combinations can only be more expensive than the cost-optimal solution by given margin, which we set to 10% for the base model runs. The process can be repeated indefinitely, each time incrementally updating the weights based on the values assumed by variables in the new feasible configuration. For a subset of SPORES, we run this process up to ten times.

To systematically explore the solution space, we apply the SPORES approach at three levels in parallel: across all technologies at once, for specific technology groups, and for electricity supply technologies alongside a secondary, technology-explicit objective. The second and third levels move from a technology-agnostic search for alternatives to one in which specific technologies or groups of technologies are targeted for minimal deployment in the system. This is repeated systematically for all electricity, heat, fuel, and transport supply technologies, as well as for storage and transmission technologies. As acknowledged by other recent applications of MGA to energy system optimization models of large size,^20^^,^^22^ the minimization of specific technologies within the selected cost relaxation margin allows to approximately capture the extreme points of the solution space. Our generation of a relatively large batch of SPORES for each of these extreme points ensures that we also find alternatives further inside the solution space. For instance, we might find alternatives in which deployment of wind is always minimized, but in which different technologies replace wind generation, or these technologies are distributed differently at the sub-national scale. For a system cost relaxation of 10%, we generate 14 SPORES with all technologies weighted equally, 119 with technologies targeted for spatial differentiation, and 308 with technologies targeted for minimization while considering electricity supply spatial differentiation. This leads to a total of 441 alternatives.

### Sensitivity analyses

We run the cost-optimization of the energy system for a full year, then apply a 10% cost relaxation for our SPORES runs. The baseline year we use is 2018, but we also run the cost-optimal run for the years 2010–2017. Since they are computationally intensive, SPORES are not run for these years. Rather, we check that least-cost feasible configurations across weather years do not lie outside the feasible decision space already outlined by SPORES for the reference weather year. Sensitivity to weather years is prioritized over other possible uncertain input parameters, such as cost and demand profiles, based on previous studies that showed it to be the parameter to which high-resolution energy system models with high shares of variable renewable generation are most sensitive.^21^^,^^71^^,^^72^ We also run a sensitivity analysis on the impact of annual demand projections, using simulated trends for changes in service demands from the models DESSTINEE^39^ and high-efficiency buildings (HEB)^40^ to update demands in the baseline year 2018 model. For the baseline year 2018, we also test a subset of SPORE runs with 5% and 15% relaxations. 120 SPORES are generated in total per cost relaxation sensitivity run, and 73 SPORES are generated in the demand projection sensitivity run. We compare the results for the equivalent runs in the baseline (10% relaxation) run. These SPORES focus on excluding specific technology groups while exploring spatial diversity of primary electricity supply. The results from the sensitivity analyses are in Note S2.

### Primary energy supply

We calculate primary energy supply according to the methodology set out in the Eurostat annual energy balances. This entails the use of lower heating value for fossil fuels, biofuels, and non-renewable waste. For renewable supply, including hydropower, wind, and solar, the primary energy supply is the electricity produced by these technologies. For nuclear power, we follow the Eurostat convention of converting the electricity generated back to the heat provided by the fission process, using our input plant efficiency of 40%. We do not include “ambient heat,” which is the heat extracted from the atmosphere when operating heat pumps. We also do not consider “Heat” (H8000), which is the heat made available from district heating systems; rather, we consider the primary energy into those systems (e.g., municipal waste). We group technologies into broader categories than those given by Eurostat’s Standard Code List,^73^ and provide human-readable names to the codes, as follows: electricity (*E7000*), other fossils (*C0000X0350-0370*, *C0350-0370*, *P1000*), oil (All codes starting in *O4000*, and *S2000*), natural gas (*G3000*), waste (*W6100_6220*), nuclear heat (*N900H*), renewables (all codes starting in *RA*[1–5]), biofuels (all codes starting in *R5*, and *W6210*).

## Data Availability

All code and data associated with this study are available on GitHub: https://github.com/calliope-project/sector-coupled-euro-calliope and Zenodo: https://doi.org/10.5281/zenodo.6546817.

## Supplemental information

Document S1. Supplemental experimental procedures, Figures S1–S26, Tables S1–S8, Notes S1–S4, and supplemental references

Document S2. Article plus supplemental information
