# Supplementary material for: Diversity of options to eliminate fossil fuels and reach carbon neutrality across the entire European energy system
Source: Joule. 2022 Jun 15;6(6):1253–76. doi: 10.1016/j.joule.2022.05.009 (PMC9220955; doi:10.1016/j.joule.2022.05.009)
Supplement: Document S1. Supplemental experimental procedures, Figures S1–S26, Tables S1–S8, Notes S1–S4, and supplemental references [file mmc1.pdf]

Joule, Volume *6*

Supplemental information

Diversity of options to eliminate fossil fuels and reach carbon neutrality across the entire European energy system

Bryn Pickering, Francesco Lombardi, and Stefan Pfenninger

SUPPLEMENTAL EXPERIMENTAL PROCEDURES

NOTE S1: SECTOR-COUPLED EURO-CALLIOPE MODEL

The model we use in this study is an extension of Euro-Calliope v1.0[1], to include all energy-consuming sectors in Europe. Euro-Calliope v1.0 includes only the power sector and only current electricity loads. This section will detail the process of extending Euro-Calliope to include each of the following sectors: household and commercial heat, passenger and freight transport, industry process heat and feedstocks, and energy consumption in all other sectors including agriculture. For each sector, demand and supply technology data has been acquired from various sources and combined in an automated workflow, openly available on GitHub. A data source overview is given in Table S1, for those data accessed for the purpose of adding new sectors. For a detailed understanding of data sources used in Euro-Calliope v1.0, refer to Tröndle et al.[2].

Table S1: Primary data sources used in Euro-Calliope model development. Resolution is given in the context of the Nomenclature of Territorial Units for Statistics (NUTS). Eurostat dataset codes are given in parentheses.

Source name	Data accessed	Resolution	Use of data	Sectors affected
**Eurostat**	Annual energy balances (*nrg_bal_c*)	NUTS0	Energy data in all subsectors	All
	Annual household energy end-uses (*nrg_d_hhq*)	NUTS0	Household end-use energy consumption	Household heat and electricity
	freight loading (*road_go_na_rl3g*)	NUTS3	Sub-regional disaggregation of industry demand	Industry
	employees by subsector (*sbs_r_nuts06_r2*)	NUTS2		
	dwelling number and types (*cens_11dwob_r3*)	NUTS3	Heat demand generation	Building space and water heat
	Gross value added by commercial subsector (*nama_10r_3gva*)	NUTS3	Sub-national disaggregation of commercial demand	Commercial building and transport
**Joint Research Centre (JRC)**	JRC IDEES[3]	NUTS0	Attribution of consumed resources per subsector to end-uses	All
	JRC open power plant database[4]	Site-specific	Location of existing conventional power supply technologies	Power
**Swiss federal office for energy (SFOE)**	Swiss equivalent of Eurostat data	NUTS0	Energy data in all subsectors, sub-regional demand disaggregation	All
**Danish energy agency**	Technology catalogue	N/A	Technology costs and operational characteristics	Heat, electricity, and renewable fuels

BUILDING HEAT SECTOR

We group heat demand in buildings into three end-uses: **space heat, hot water, and cooking**. These groups match the Eurostat household end-use categorisation, national data for which became available in 2020 (nrg_d_hhq). This data has been used to assign the consumption of fuels to different end-uses. We infer building heat demand in commercial and industry sectors from the JRC IDEES database[3]. We then transform the consumption of fuels to a demand for heat by assuming technology efficiencies of heating technologies including boilers and direct electric heaters (see Table S2). These efficiencies are consistent with those used in the Sector-Coupled Euro-Calliope for the respective heat supply technologies. Heat pumps are a special case, since heat demand can be calculated using the consumption of ambient heat: demand = ambient heat consumption + electricity consumption. We use these annual water and space heat demands to scale hourly demand profiles produced using the methods implemented for the When2Heat database[5], updated to account for (a) all Euro-Calliope countries and (b) the sub-national distribution of single- to multi-family homes across Europe, according to the Eurostat database of dwellings (cens_11dwob_r3). We generate cooking heat demand profiles using a bottom-up stochastic modelling. Our approach extends the open-source RAMP engine [6], developed and validated in previous work[7] with application to Italy, to stochastically model demand in all European countries.

To meet these demands, we define a range of new technologies in the model, key data for which can be found in Table S3. We source most data for these from the Danish Energy Agency Technology Catalogue[8]. Again, heat pumps are a special case, since their performance is weather-dependent. Our heat pump coefficients of performance (COPs) are based on a catalogue of 78 heat pumps provided by the manufacturer WAMAK. The WAMAK heat pump performance data represents state-of-the art technology and generally fits that given by previous studies (Figure S1)[5,9,10]. Following Ruhnau et al.[5], we assume COP to be 80% of published performance. The WAMAK catalogue data also includes actual technology heat delivery capacity, relative to nominal capacity (Figure S2). That is, the nominal capacity in which one invests is not the actual capacity that is realised, which instead depends on sink and source temperatures. We describe heat pumps using hourly COP and capacity variation; both timeseries rely on hourly temperature data from the MERRA-2 reanalysis[11] [a].

Table S2: Heat technology efficiencies used to translate consumed energy resources into demand for end-use heat.

	Technology	Efficiency
**Space and water heating**	Gas (natural gas, biogas)	0.97 [12]
	Petroleum products	0.9 [12]
	Solid fossil fuels	0.8 (assumed the same as for solid biofuels)
	Solid biofuels	0.8 [12–14]
	Solar thermal	1.0 (as per Eurostat energy balance methodology)
	Direct electric	1.0
**Cooking**	Gas (natural gas, biogas)	0.28 [15]
	Petroleum products	0.28 (assume same as gas)
	Solid fossil fuels	0.15 [16] scaled using [15]
	Solid biofuels	0.1 [16] scaled using [15]
	Direct electric	0.5 [15]

[a] *Air-source heat pumps use surface air temperature while ground-source heat pumps use sub-surface temperature (tsoil5) – 5°C, to account for heat transfer to the ground loop brine*

Table S3: Key data for heat supply technologies in the Sector-Coupled Euro-Calliope. Data is almost exclusively 2050 estimates from the Danish Energy Agency technology catalogue[8]. Greater detail can be found in the model implementation.

Technology	Energy input	Efficiency	Capital cost (EUR2015/kW)
methane boiler	Methane	97%	172
biofuel boiler	Biofuel	80%	445
air source heat pump	Electricity	Time varying COP	662
ground source heat pump	Electricity	Time varying COP	1100
solar thermal panels	Solar irradiance	Time varying efficiency	515
direct electric heaters	Electricity	100%	695
Combined heat and power plants	Waste / biofuel / methane	Depending on heat to power ratio	520 - 2783
hot water storage	Heat	0.01-0.02%/hour	3 (large-scale) – 410 (small-scale) EUR2015/kWh

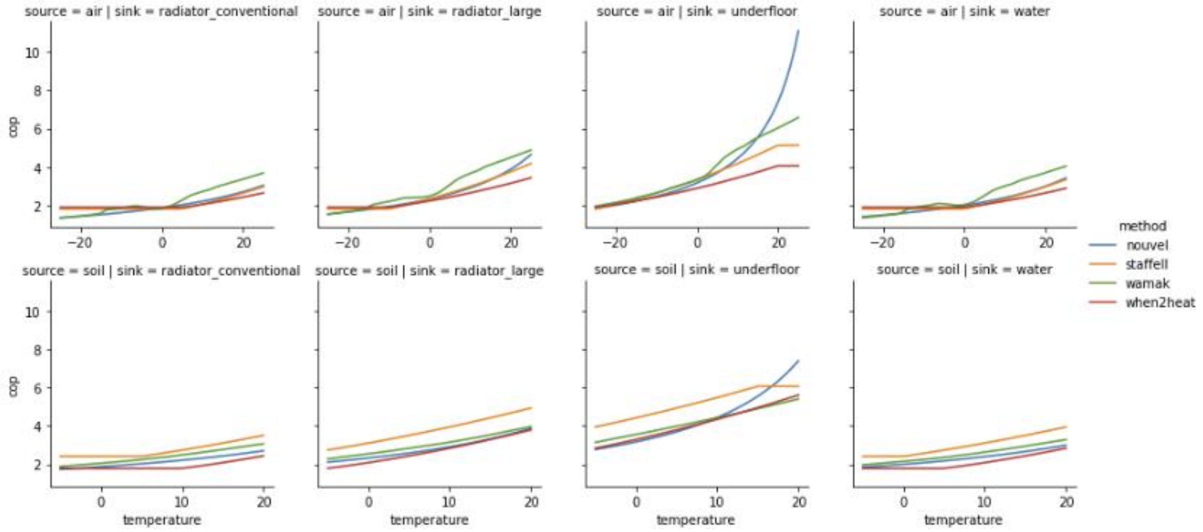

Figure S1: Comparison of heat pump coefficients of performance for different source and sink temperatures, as well as different technology types (air-source and ground-source heat pumps). Methods are named based on first authors of the respective studies[5,9,10], except WAMAK which is the manufacturer name from which performances were manually extracted from a catalogue of technologies.

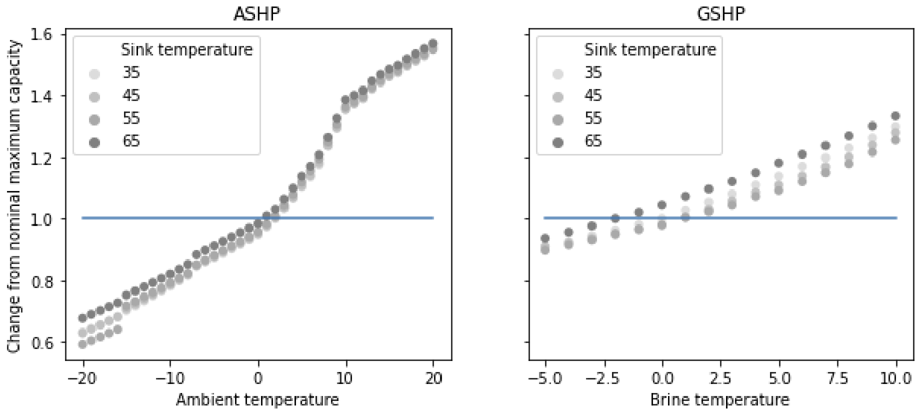

Figure S2: Change in heat pump delivery capacity as a function of source and sink temperature (ºC), based on the average performance of several heat pumps in the WAMAK catalogue of technologies. ASHP: air-source heat pump, GSHP: ground-source heat pump.

TRANSPORT SECTOR

The transport sector encompasses road, rail, air, and shipping. Electrification is only possible in some of these forms of transport, namely road and rail. In rail, we assume complete electrification is possible, such that all current fuel oil demand is replaced by direct electricity demand in 2050. The current consumption of fuel is taken from Eurostat, while the efficiency of different rail drive trains is taken from JRC IDEES. For air and shipping, we assume the opposite: there will be no electrification by 2050. Instead, the kerosene and diesel demand of these two forms of transport must be met by synthetic fuel generation. Accordingly, air and shipping (domestic and international) demands are taken directly from Eurostat. Unlike the other modes, we do not assume a 'winning' drive train for road transport. Instead, we calculate the distance travelled by all vehicles in each country and use this distance as the demand in the model. We use annual vehicle mileage from JRC IDEES, split into motorcycles, passenger cars, busses, light-duty commercial vehicles, and heavy-duty freight vehicles. Vehicle mileage is then transformed back to energy demand based on the efficiency of different drivetrains. The energy consumption per unit distance data given in Table S4 are based on the 25th percentile of all countries' vehicle energy consumption, as given by JRC IDEES for the year 2015. This represents a convergence on higher efficiency of vehicles in all countries in Europe, but not an improvement in countries with existing efficient vehicle fleets.

We assume only light-duty electric vehicle and passenger rail demands have hourly profiles impacting energy delivery; all other demands must be met on an annual basis, since they are synthetic fuels. We take rail electricity demand profiles from the DESTINEE demand model[17]. Electric vehicles are limited in the allowed energy delivery per hour based on the number of vehicles connected to the grid at any given time. We generate this plug-in profile using RAMP-Mobility[18], an extension of the aforementioned open-source RAMP engine[6]. The result is that in some hours, as few as 70% of electric vehicles are plugged in (Figure S3). We base the available charge capacity of plugged-in vehicles on the number of vehicles and an average battery size[19]. The result of this is that if the model chooses to electrify half a region's vehicle fleet of 100 cars, then there will be a maximum of 50 cars plugged in, each with a battery of 0.08MWh. Thus, 4MWh of energy can be delivered to vehicles in that hour. This method allows the model to decide when to charge cars (smart charging), but ensures that it is not unrealistic in the frequency of charging throughout the year (i.e. it cannot choose to charge all vehicles in one week of the year). However, initial tests showed that this was still not sufficient to ensure "realistic" EV charging, with regions having little to no EV charging in January weeks (Figure S4). Accordingly, a bound on EV supply was applied to ensure charging demands are met in every month, using monthly EV electricity demand from RAMP-mobility (Figure S3).

*Table S4: Average vehicle fleet energy consumption by drivetrain (oil or electricity driven) and battery capacity of electric vehicles. Vehicle classes and energy consumption values are based on JRC IDEES; energy consumption is the 25th percentile of energy consumption across all JRC IDEES countries in 2015. Battery capacity is an average of values given by The European Council for Automotive R&D (EUCAR)[19]. *these values are not given by EUCAR, so are assumed.*

	**Energy consumption (MWh/million km)**		**Battery capacity (MWh)**
**Vehicle class**	*Oil*	*Electricity*	
Heavy duty vehicle	5140	N/A	0.2
Light duty vehicle	855	480	0.1*
Motorcycle	419	200	0.01*
Bus	6057	3248	0.2*
Passenger car	675	324	0.08

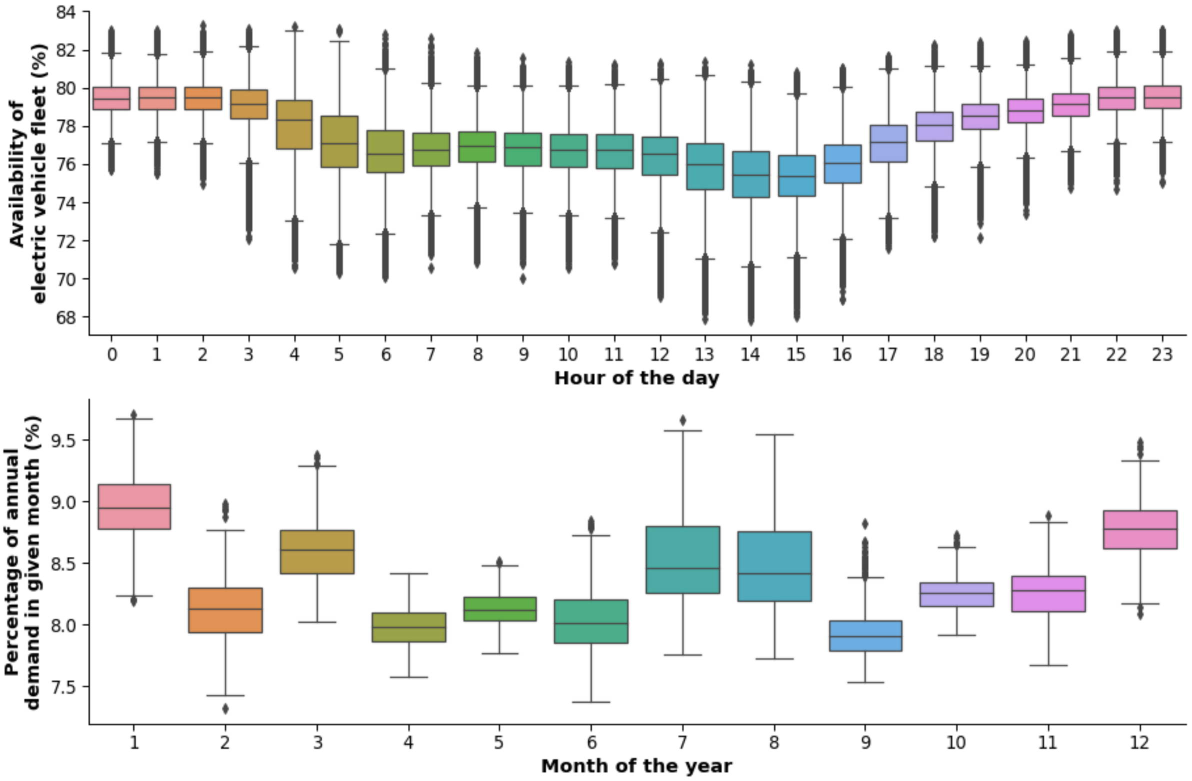

Figure S3: Overview of Euro-Calliope electric vehicle hourly plug-in schedule (top) and monthly demand (bottom), based on national stochastic profiles modelled in RAMP-mobility. Boxplots show the variation in availability per hour of the day (top) and month of the year (bottom), across years 2000-2018. Top: All hours are given in UTC+00:00, not local time.

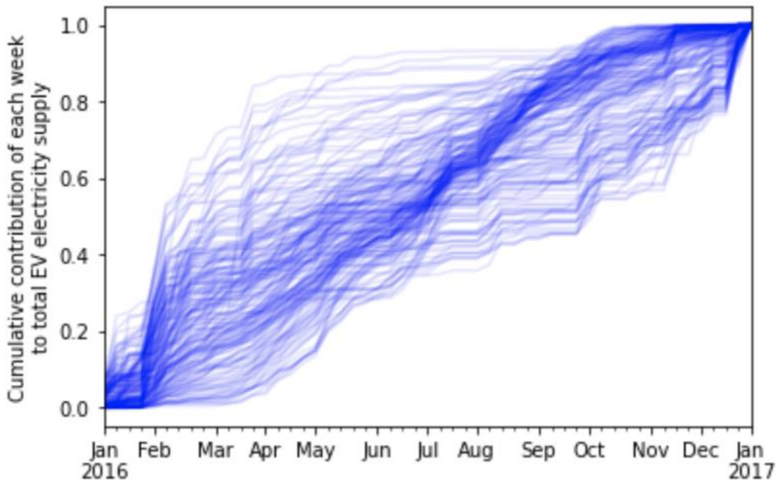

Figure S4: Normalised cumulative weekly EV charging across the 2016 weather year, based on results from optimising cost in the Euro-Calliope model with only a requirement to meet demand by the end of the year. Lines represent all 98 Euro-Calliope model regions and both electrified heavy-duty and light-duty vehicles. Each line has a low opacity, so high opacity areas indicate a greater degree of overlap.

Industry sector

The industry sector has demand for different levels of heat (space heating, low temperature heat, and high temperature heat), for already electrified or electrifiable end-uses (namely, operation of machinery), and for consumption of energy resources as feedstock (such as oil for the production of base chemicals). There are no European statistics on the breakdown of fuel consumption that can be attributed to each end use, but some countries publish their own statistics. Countries with sufficiently disaggregated demand data are Germany, Austria, and the UK. Switzerland also has disaggregated data, for *either* the fuel consumption per industry subsector or the end use demand of industry in total, but not the fuel consumption per end use per industry subsector.

Both bottom-up and top-down approaches to understanding industrial energy demand have been undertaken to date[3,20–23]. Top-down modelling attempts have mapped the end use demand of industry subsectors from a subset of contexts to all countries in Europe; for instance, Naegler et al.[21] use the German industry data in their analysis. Mantzos et al.[3] also undertake a top-down analysis to create the JRC-IDEES database, although it is unclear whether any country-specific end-use data was used to inform their model. Bottom-up modelling has used the FORECAST-Industry demand modelling tool[20,22,23], which demands a level of data input that is beyond the scope of our study. The HeatRoadmap Europe project[20] is an extension of Rehfeldt et al.[22], with the focus on a different reference year (2015 and 2012, respectively). There is no ground truth for industry end use demand, and datasets rarely align. Therefore we utilise the JRC-IDEES database, given that its structure best matches the data on total sectoral energy consumption published by Eurostat, as well as the use of 13 subsectors. JRC-IDEES provides sufficient data to understand electrified and electrifiable processes, as well as high and low temperature processes. We assume that steam processes still require methane and that diesel-powered backup generators are still in use for when grid access is disrupted**,** while all other processes can be electrified, according to the efficiencies provided in JRC-IDEES. A remaining issue is the consumption of fossil fuels as feedstock to industrial processes. Such consumption contributes to a large proportion of emissions in the chemicals industry[24] as well as for iron production[25]. To mitigate these emissions, new methods or feedstocks are required in the chemicals and steel industries, as will be detailed in the remainder of this subsection.

Iron and Steel

The process for producing steel requires two key steps: 1. iron ore to iron, and 2. iron to steel. In its current form, the first step is almost entirely conducted using *Blast Furnaces* (BFs) to produce "pig" iron (high carbon content iron) which cannot be fully decarbonised without CO_2 capture, due to the reliance on unreplaceable coke as the iron ore reductant[26,27]. A small (~6%) quantity of iron is produced as *Direct Reduced Iron* (DRI)[28], which relies on hydrogen (via natural gas) instead of carbon to reduce the iron ore. Although a proven process, the cost of reducing agent prohibits its large scale deployment[27].
The second step is currently dominated by the use of a *Basic Oxygen Furnace* (BOF), which reduces the carbon content of pig iron, to produce steel. The BOF requires large amounts of heat, as well as a source of oxygen (usually air), and is hard-linked to the BF as one unit, the *Blast/Basic Oxygen Furnace* (BF-BOF). This route releases CO_2 in the combustion of fuels, the production of lime and coke, and in the removal of oxygen from iron ore and excess carbon from pig iron, to produce steel[25].

The steel sector is highly circular, already around 85% of produced steel is recycled and approximately 40% of steel produced in Europe is from scrap[27]. Scrap steel enters the BF-BOF route, with blast furnace heat used to melt the scrap for addition into the basic oxygen furnace alongside iron, at about 10-20% of input ferrous material[25]. The remaining (majority of) scrap is processed using *Electric Arc Furnaces* (EAFs), which is an entirely electrifiable route of melting and recasting steel that accounted for 29% of all crude steel production in 2018[28].

Without carbon capture, the current BF-BOF cannot be fully decarbonised; only parts of the process could be replaced with biomass-based alternatives[25–27,29,30]. However, the direct reduced iron route could be decarbonised by direct use of hydrogen, instead of extracting it from natural gas. This process, known as H-DRI, could produce iron with low to no emissions. Following this, the electric arc furnace could be used as the primary method to produce steel; scrap steel and iron would be combined with a splash of carbon from coal to produce crude steel[27]. A study as part of the Hybrit project found that the H-DRI-EAF route would emit approximately 97% less CO_2 than the BF-BOF route, for the same crude steel production[31]. A less well developed, but potentially more energy efficient route for iron production involves direct electrolysis of iron ore (electrowinning). This technology is still at the laboratory phase[32], so little is known about its energy consumption at an industrial scale[b]. Indeed, it is explicitly not considered in the scenarios presented by the Material Economics consortium[27].

In the Sector-Coupled Euro-Calliope, we consider H-DRI-EAF as the primary route for future, decarbonised steel production, with an expected increased use of recycled steel overall to 50% of total ferrous material input (see Figure S5). This is in line with the Material Economics "new processes" pathway. Unlike these pathways, but similar to Hybrit, we would not only consider biomass sources of hydrogen, but also (and primarily) production by electrolysis from excess renewable generation. The hydrogen requirement for H-DRI is approximately 51kg per tonne of steel output[31], not accounting for the use of scrap steel in the EAF. That is, if 50% of the ferrous input in the EAF is scrap steel, and 50% is iron from H-DRI, then 25kg of Hydrogen would be required. In addition, electricity is needed for both the H-DRI and EAF processes. The Hybrit pre-feasibility

[b] *Fischedick et al.[32] use 9.3GJ/t crude steel in their calculations.*

study gives 2,633 kWh electricity for Hydrogen production, 322kWh for H-DRI, and 494kWh for EAF (+380kWh biomass and 42kWh coal), all per tonne of crude steel. Vogl et al.[31] agree on the electrolyser electricity consumption, but give 753kWh$_e$/t for EAF, approximately 250kWh/t for heating of iron ore, and <50kWh/t for H-DRI. Both ultimately give approximately 3,450kWh$_e$/t for the whole process, if no scrap steel is used, which is similar to the 3,640kWh$_e$/t given by Fischedick et al.[32]. On top of this, iron ore pelletising/sintering and downstream steel casting/rolling require 833 and 28/805 kWh, respectively[33].

Assuming 50% scrap and the production of one tonne of liquid steel, consumption of energy becomes: 25kg H_2, 135kWh$_{th}$ for iron ore heating and use in the H-DRI process, 710kWh$_e$/t for EAF, and 111kWh$_e$+625kWh$_{th}$ for pelletising, sintering and continuous casting[c]. Additionally, the "sponge" iron (output from H-DRI) can be allowed to cool and stored, effectively providing a buffer between energy production for H-DRI and for EAF; however, 159kWh/t is then required to reheat the iron for use in EAF. We ignore the requirement for coal and lime in the steel-making process but include energy demand for product 'finishing', as given by JRC-IDEES[3], which comes in at around 60-70kWh$_{th}$/t.

Quantity of steel production

For each country, we require the quantity of produced steel to understand future energy demand. Very few countries publish this data as part of Eurostat's PRODCOM database, so we instead use data on annual production for a select number of countries[28] to verify that energy consumption for iron and steel correlates with annual production, then map that to all Euro-Calliope countries.

All European countries (not including Turkey, Ukraine, Russia, and other CIS members) produced 172.8Mt of crude steel in 2018, 100Mt of which was produced by the BF-BOF route and the remaining 72.8Mt by EAF. BF-BOF derived steel is well matched to blast furnace energy consumption given by Eurostat (99.3% Pearson correlation; Figure S6a). All steel industry energy consumption is marginally less well matched to all crude steel production (97% Pearson correlation; Figure S6b), but still offers a useful avenue to disaggregate European steel production. In 2050, it is predicted that steel production will have increased across Europe to 199Mt[d]. We disaggregate this value to countries using total iron and steel subsector energy consumption.

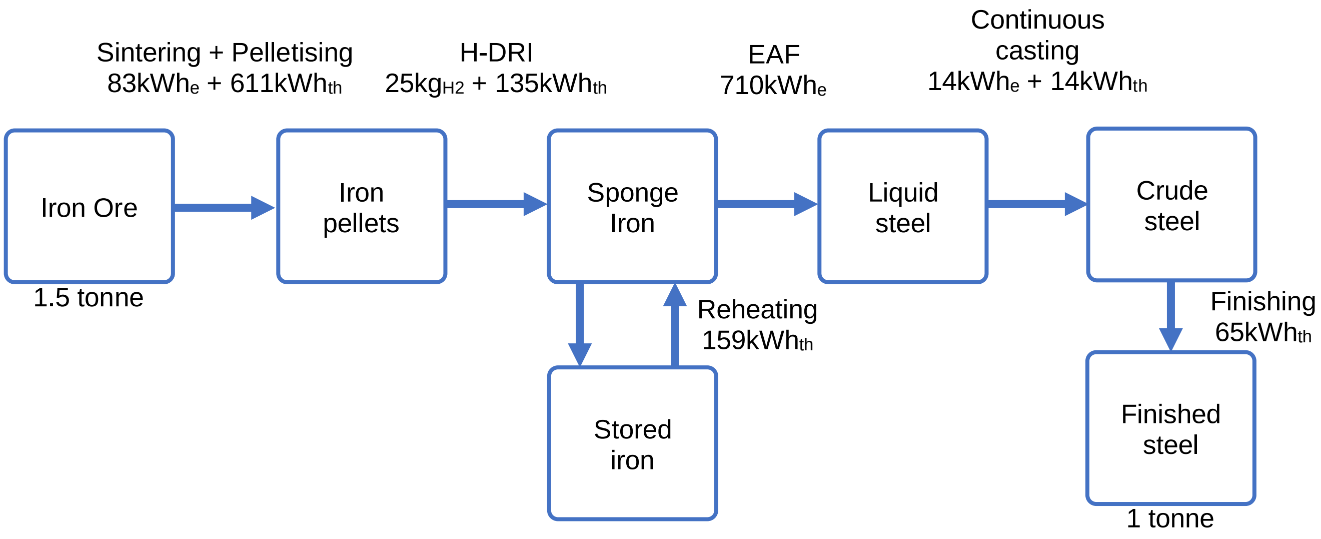

Figure S5: Iron -> Steel processes and the energy requirements to produce one tonne of cast steel.

[c] *We take the lower bound energy consumption for casting/rolling (i.e. for casting) since continuously cast steel makes up 97% of total crude steel production in the EU[28].*

[d] *193Mt from EU [27] + 6Mt from rest of Europe, using the same methodology of a 15% increase in steel production from 2016 to 2040.*

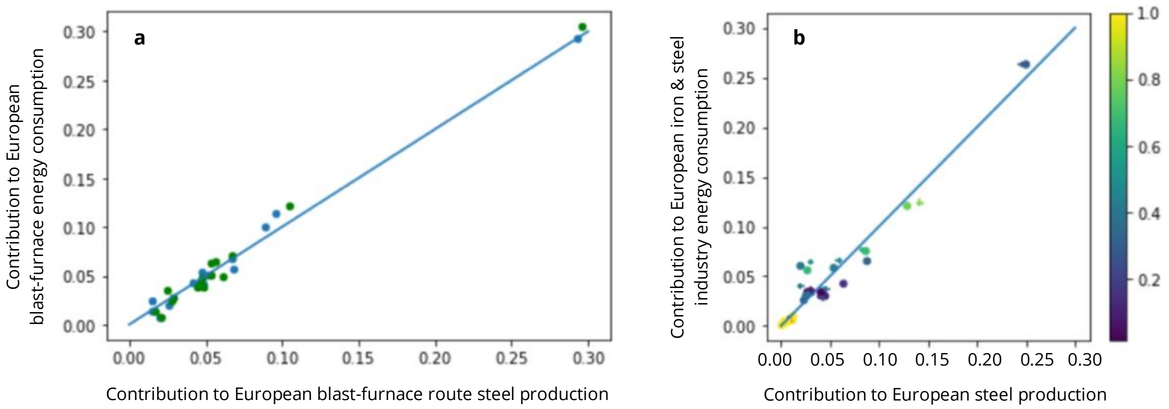

Figure S6: (a) Contribution of each European nation to total European BF-BOF steel production compared to national contribution to European blast furnace energy consumption. Blue = 2015, Green = 2018. (b) Contribution of each European nation to total European steel production compared to national contribution to European iron and steel industry energy consumption. Circles = 2015, crosses = 2018; colormap gives relative contribution of EAF to total steel production in each country. In both subplots, line shows correlation=1.

Chemicals

The chemical and petrochemical industry covers the production of end-use plastics, fertilizers, pharmaceuticals, and many other chemicals (glues, cleaning fluid, etc.). Of these, it is petrochemicals that require fossil feedstock; ammonia (for fertilisers), high value chemicals (HVCs, for plastic production), and methanol (for plastics and other chemicals) account for 90% of fossil feedstock in the chemical industry[34].

Plastics

There is a wide variety of plastics, almost all of which originate from the “cracking” of naphtha or liquified petroleum gas (LPG) into “high value chemicals” (HVCs) such as ethylene, propylene and BTX (benzene, toluene and mixed xylenes). The route to generating HVC without fossil fuel cracking is via methanol[35], which can be synthesised by hydrogenation of CO_2 or gasification/pyrolysis of biomass or waste plastics. Methanol synthesis is essentially the same as production of synthetic natural gas or synthetic liquid fuels, just with different compositions of the input gases. The energy requirements of these routes are given in Bazzanella and Ausfelder[35] and are (sometimes loosely) used by Material Economics[27] in its pathway generation. The “new processes” pathway assumes that chemical plastic recycling (40%) and biomass-to-plastics (33%) will dominate plastic generation in 2050, along with mechanical recycling (13%) and improved circular economies (14%). Figure S7 gives the process chains to realise final plastics production, with the inclusion of the option of hydrogen to plastics[35]. Energy requirements in some processes are ignored in Material Economics[27], and indeed there is little information available on some processes, including chemical recycling. Nevertheless, it is understood that external energy sources will be required to maintain e.g. the 900C required in a gasifier[36]. An additional route, mechanical recycling, could require 7MWh/t HVC[35], but this magnitude is too high relative to the available data on chemical recycling. Indeed, from various Swedish sources, Liljenström and Finnveden[37] found mechanical recycling to have an average energy consumption of 0.37MWh/t HVC.

According to the Material Economics “new processes” pathway, 28.8Mt of plastics in Europe will be generated by chemical recycling, 23.8Mt by new production (biomass or hydrogen), and 9.36Mt by mechanical recycling; an additional 10.1Mt would be circulated internally, so we do not consider them as energy consuming. This contrasts with the 64Mt of production in 2016 (60Mt by conventional means) given by Plastics Europe[38], and 47Mt of HVCs currently produced in Europe, according to the International Energy Agency[34]. The discrepancy between HVCs and plastics probably stems from imports of HVCs as well as production from other base chemicals.

Some countries have a greater discrepancy than others in their production of HVCs and subsequent production of end-use plastics. The Netherlands consumes 4.3% of Europe’s converter plastics (penultimate step before reaching end-use plastics)[38] but produces 18% of EU-28’s ethylene+propylene (see PRODCOM database), while Germany both produces 24.6% of those two chemicals and consumes 24.6% of Europe’s converter plastics. Since we are concerned with replacing fossil feedstocks, we will focus on the generation of HVCs, and therefore use non-energy consumption of Naphtha from the Eurostat annual energy balances to infer national contributions to European production of HVCs. Figure S8 shows that this is a reasonable assumption, with Naphtha consumption more often matching PRODCOM data compared to plastics production data. This

comparison comes with the caveat that many countries have no PRODCOM data, and BTX HVCs are not included (again, for lack of PRODCOM data).

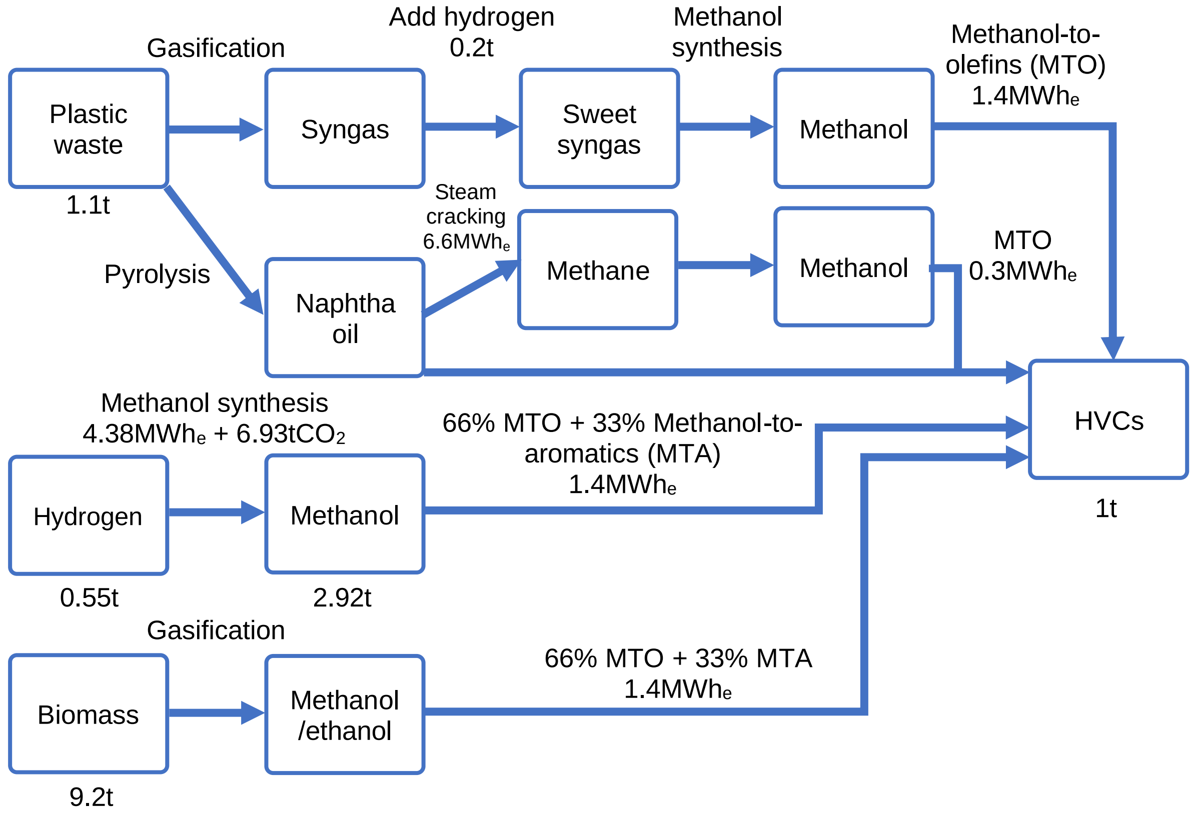

Figure S7: Possible routes to zero/low carbon plastic production, based on a combination of Material Economics[27] and Bazzanella & Ausfelder[35]. There are some data gaps with no reliable source, including energy demand for plastic gasification and pyrolysis. Biomass gasification energy use is incorporated into its overall efficieny (hence the high input requirements).

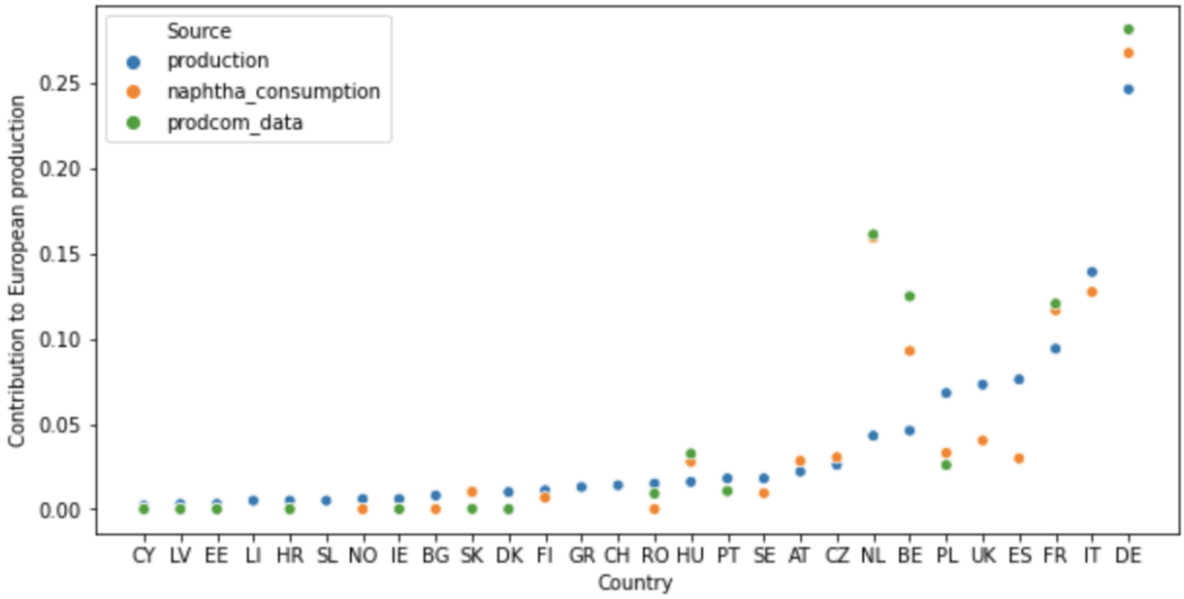

Figure S8: Comparison of data sources to infer the contribution of countries to European HVC production in 2018. 'production' is the contribution to production of end-use plastics according to Plastics Europe[38], Naphtha consumption is from Eurostat energy balances, and 'prodcom_data' relates to published data on produced volume of Ethylene and Propylene, where available.

Ammonia and Methanol

Produced from steam reformation of natural gas or gasification of coal, ammonia and methanol rely on a source of hydrogen for their production. Ammonia is produced by combination of hydrogen and nitrogen, while methanol is produced by the combination of hydrogen and CO_2. Of the replacement fossil feedstocks, this is the most straightforward: Ammonia requires 0.178t_{H2}/t + 1.73MWh_e/t for compression and N_2 production; Methanol requires (as seen in the previous subsection) 0.189t_{H2}/t + 1.5MWh_e/t + 1.373t_{CO2}/t[35]. An additional component of this process chain is urea, which currently relies on the steam and CO_2 output of Ammonia production, adding 0.92MWh/t + 0.32t_{CO2}/t urea. Table S5 shows the annual production of Ammonia, which ranges from 15 to 28Mt in a year. Given the collection of values in the range 15-19 Mt, we take 17Mt as the annual production, as given by Bazzanella and Ausfelder[35]. Their number is based on a dataset from Fertilizers Europe which is no longer available. We also take the urea production from the same source: 6Mt, leaving 13.6Mt for direct ammonia and 3.4Mt for ammonia-> urea[e].

Table S5: Annual ammonia production in Europe (EU or all Euro-Calliope regions) according to various sources.

Source	Year	Quantity
International energy agency[34f]	N/A	27.5Mt ammonia
PRODCOM	2017	16 Mt ammonia (13.1 Mt N)
USGS[g]	2017	18 Mt ammonia (14.6 Mt N)
Boulamanti and Moya[39]	2013	19 Mt ammonia
Fertilizers Europe	2017	15 Mt ammonia (12.4 Mt N)
Bazzanella and Ausfelder[35f]	2016?	17 Mt ammonia

Bazzanella and Ausfelder[35] depend on PRODCOM for methanol production data, which was 1.6Mt in 2018. This compares to 4.5 Mt given by the International energy agency[34]. Global demand for Methanol was 75 Mt in 2015, 3% of which was produced in the EU[34], giving 2.6 Mt. Based on these fluctuating values, we take a value of 2 Mt.

Molar contributions

To match with JRC IDEES basic chemicals (given in weight of ethylene), we take their values of production and attribute them by molar ratio to plastics, ammonia, and methanol. Results of this are shown in Table S6. Applying this to the JRC IDEES dataset leads to an overprediction of chemical production (by about 10-15%); this is expected, since we do not cover all basic chemicals in this analysis.

Table S6: Comparison of basic petrochemicals, including their approximate annual production in Europe, molar mass, and molar contribution to total moles of basic chemicals produced.

Chemical	Annual production (Mt)	Molar mass	% molar share of chemicals
Ethylene	21.7	28.05	32.1
Propylene	17.0	42.08	16.8
BTX	15.7	93.00[h]	7.01
Ammonia	17.0	17.01	41.5
Methanol	2.00	32.04	2.59

In the Sector-Coupled Euro-Calliope, we consider chemical production as requiring a combination of three molecules: CO_2, H_2, and methanol. Together, these can be used to produce the basic chemicals for plastics, as well as ammonia and urea. We estimate the quantity of each basic chemical produced in Europe, and assume that each country produces the same relative share of those chemicals. With this assumption, we can disaggregate the JRC IDEES estimate of national production of basic chemicals (in kt ethylene) to the chemicals of interest, and from there calculate the demand for CO_2, H_2, and methanol based on data of each transformation technology[35]. This gives the annual demand for CO_2 (kt), H_2 (MWh LHV), and methanol (MWh LHV) for the chemical industry of each country.

[e] *0.57 tonnes ammonia is needed per tonne of urea[35].*

[f] *Base source is Fertilizers Europe, but data nitrate subsets is only available to paying members.*

[g] *Only covers 22 of the Euro-Calliope countries (probably the biggest producers). Alternatively, taking total global production of 144Mt multiplied by the 9 and 12% contribution of the EU, according to fertilizers Europe and the IEA, respectively, we get 12.9-17.3Mt.*

[h] *Average of the components of BTX, which range from 78 to 106 g/mol.*

Other industry subsectors

Feedstocks are not such a concern in other industry subsectors, so we use JRC IDEES data directly. The only changes we envision in these subsectors is the electrification of machinery and medium temperature processes. The only subsector which produces notable emissions through processes that cannot be decarbonised is the cement industry. There is speculation about the possible avenues for processes and materials that could be used to mitigate cement industry emissions[24,27], but these are rarely energy-based solutions. Therefore, we ignore non-energy emissions from the cement industry.

OTHER SECTORS

The final sectors not covered by any of the previous subsections are agriculture & forestry, fishing, and “not elsewhere specified”. These sectors account for approximately 2.5% of total European annual energy demand. According to Eurostat, “not elsewhere specified” demand is attributed to the military, among other things. These sectors are not handled completely by JRC IDEES, so we take a different approach. We assume all oil consumption to be for mobility and added to annual demand for heavy-duty vehicles (*agriculture & forestry* and non-kerosene use in *not elsewhere specified*), shipping (*fishing*), and aviation (kerosene in *not elsewhere specified*). We assume all other non-electricity consumption to be for heating applications, and therefore is added to annual commercial heat consumption.

SYNTHETIC FUEL PRODUCTION

All subsectors have the option, or indeed the requirement, to meet demand with net-zero emission hydrocarbons (henceforth ‘synthetic fuels’). Kerosene, diesel (used also as a proxy for petrol), methanol, and methane are energy carriers in the Sector-Coupled Euro-Calliope. All of these fuels can be generated from electricity or biofuels. If generated from electricity, then hydrogen and CO_2 are first produced from electrolysis and direct air capture, respectively, before being combined in various processes to produce the hydrocarbons. We have collated these processes and associated technologies almost exclusively from the Danish energy agency technology database[12], with the exception of direct air capture of CO_2 (from Fasihi et al.[40]) and electrolysis data (following the same approach taken in Lombardi et al.[41]).

EURO-CALLIOPE REGIONALISATION

To represent the geographic disparity across all energy-consuming sectors, a sub-national spatial resolution is necessary. However, two issues arise when moving to a subnational level: (1) administrative regions are often not at the correct resolution or are at significantly different resolutions between two countries[i], and (2) the transmission system is not well understood by modellers below the national level. Between countries, Net Transfer Capacities (NTCs) are available from European Network of Transmission System Operators for Electricity (ENTSOE). At a sub-national level, this is rarely the case. To address both these issues we adopt a regionalisation first developed within the European Commission Seventh Framework Programme project e-HIGHWAY 2050. Within this project, a pan-European transmission system representation was developed based on grouping NUTS3 administrative regions to 106 model regions[42]. Grid transfer capacities between model regions were calculated in e-HIGHWAY 2050 based on a detailed, proprietary understanding of the transmission system. By assuming the same model regions, we are able to ensure a high resolution as well as a detailed understanding of transfer capacities of the transmission system in the Sector-Coupled Euro-Calliope. Figure S9 shows the final 98 model regions[j], including all possible inter-region high-voltage transmission connections. These connections include those already existing in e-HIGHWAY 2050 as well as planned connections in the medium and long term, predominantly high voltage DC (HVDC), according to ENTSOE 2018 network development plan (TYNDP). In the model, we use the capacities of these existing/planned transmission lines as a lower limit, then allow lines to increase at a cost. Upper limits on lines are set to allow initially low capacities to increase more than initially high capacities: up to 40x (<1GW), up to 10x (1-5GW), up to 5x (5-10GW), up to 3x (10-15GW), up to 2x (>15GW). These upper limits were chosen after testing different limits, to enable possible renewable generation hubs (e.g. northern Scandinavia) to increase transmission capacity sufficiently, whilst not allowing excessive capacity increases in already congested regions. The overall upper bound on system-wide capacity increase is 8.9x. We use known costs or expected costs of TYNDP planned connections to estimate transmission expansion costs in Euro-Calliope, grouping transmission lines into five types based on technology and geographic context (Figure S10). Each line type cost spans a relatively large range; we take the median cost for the baseline Euro-Calliope model in each instance.

---

[i] *For instance, Germany has 401 NUTS3 regions, while France has 101; France has almost twice the land area, and 80% the population of Germany, but has ¼ the number of NUTS3 regions.*

[j] *There are 98 model regions in Euro-Calliope compared to 106 in the original e-HIGHWAY 2050 model due to not including neighbouring countries (incl. Russia, Belarus, Ukraine, & North African countries).*

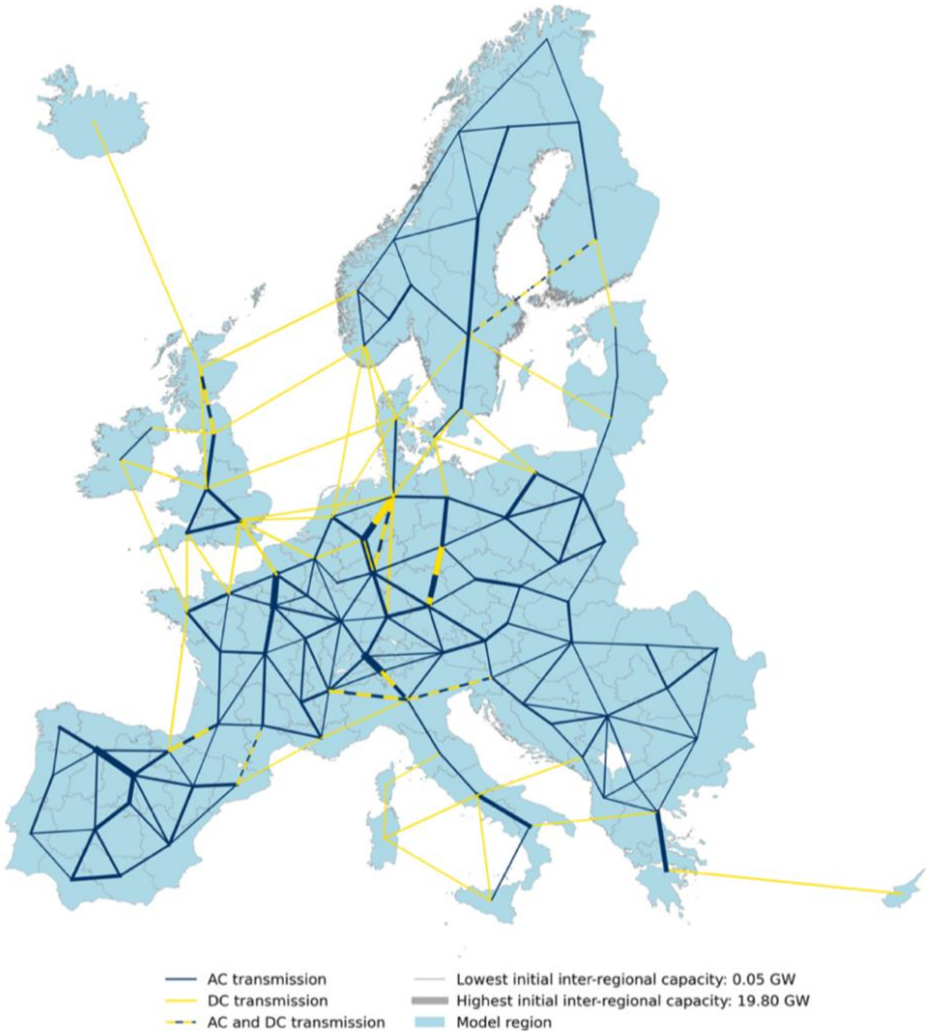

Figure S9: Sector-coupled Euro-Calliope 98 regions and inter-regional transmission lines. Lines are coloured based on the type of transmission available between regions. Thicker transmission lines represent larger modelled initial grid transfer capacities. All lines can expand beyond their initial capacities.

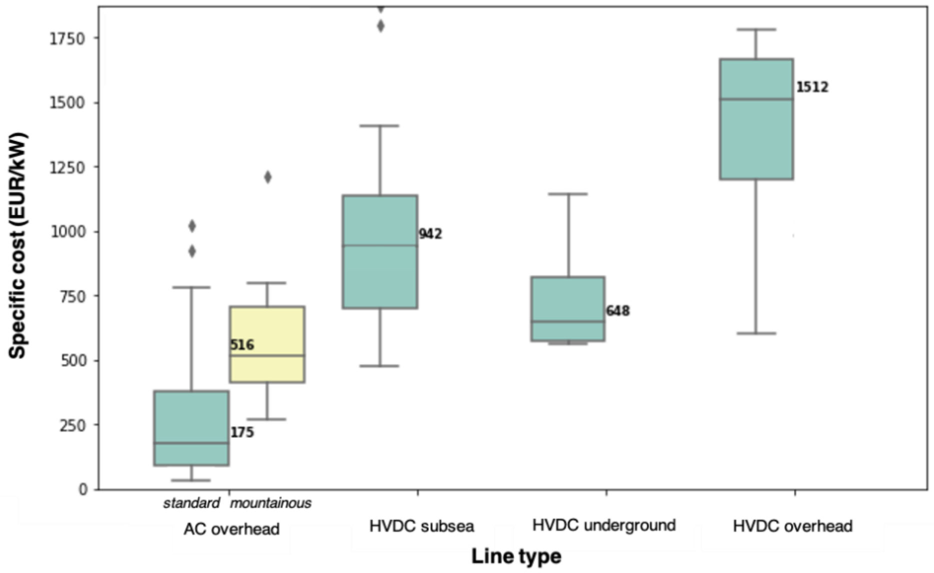

Figure S10: Distributions of transmission costs for five line types. AC = Alternating current, HVDC = high voltage direct current. AC lines are split into those which span mountainous regions and those that do not. Distributions are based on costs of implementing recent line extension projects as well as budgets for planned extensions; there are 92 data points in total.

To regionalise sub-sectors, we use different datasets for different end-uses. In each case, we compared several sub-national indicators to samples of published regional data, to test their viability. We regionalise **household and public and private passenger transport demand using populatio**[k]; **commercial building and light-duty vehicle demand using NUTS3 Gross Value Added** (GVA) from non-industrial subsectors (classifications *G-U*); and **industry demand, including from freight transport, is regionalised depending on subsector**. For industries with emitters registered in the EU-ETS, we use the location and size of emitters in 2014 for regionalisation (Figure S12). We found that these largest emitters capture most subsector emitters in each country, when compared to Eurostat annual emissions balances within each subsector (Figure S11). For all other subsectors, we found the number of employed individuals in each industry sub-sector (NUTS2) to be the best indicator, but it did not provide sufficient resolution for full disaggregation. We achieve NUTS3 regionalisation by combining number of employees with the quantity of loaded freight in each industry subsector. We regionalise **demand for aviation and shipping fuels based on average industry regionalisation**, on the assumption that these fuels would be synthetically generated in industrial regions, rather than exclusively at the point of consumption (e.g. major ports for shipping fuel).

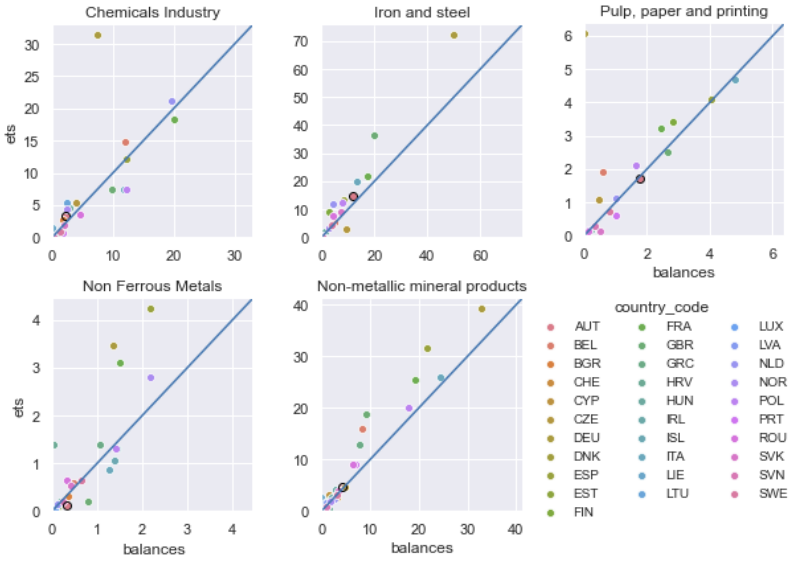

Figure S11: Published industry subsector CO_2 emissions ("balances") compared to the sum of reported emissions from industry sites within each subsector, via the EU-ETS ("ets"). Extreme outliers are caused by reporting inconsistencies; e.g. Chemicals industry emissions in Germany are not reported in Eurostat under the subsector emissions, but instead grouped into 'Other industry emissions'.

[k] *In the UK context, we found that subnational gas and electricity demand [43] was best correlated with population. Other indicators we tested include built environment land-use, GDP, and heating degree days.*

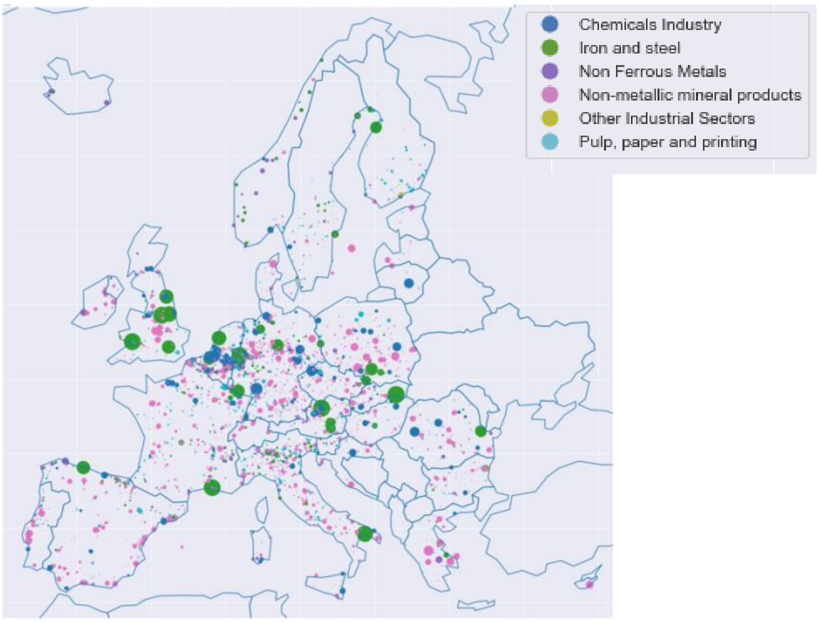

Figure S12: Spatial distribution of all sites which report to the EU-ETS / ERPTR and whose data has been collected in the Hotmaps database (3411 sites) as well as a further scraping of the EU-ETS database (722 additional sites). Marker colour depicts subsector classification, while the marker size relates to quantity of actual emissions reported for the year 2014.

POWER SECTOR UPDATES

Although our primary aim in updating Euro-Calliope was to add all non-electricity energy sectors to the existing model, we also made some updates to the v1.0 power system model. As well as the aforementioned updates to the transmission system representation, we have included 2050 nuclear power and combined cycle gas turbine (CCGT) technologies, current underground gas storage capacity from GIE, and updates to hydropower capacity. We base nuclear capacity in 2050 (Table S7) on published data of expected capacity in select countries, regionally distributed by current capacity from the JRC open power plants database[4]. CCGTs are expected to consume synthetic methane and they have no constraints on total capacity. We take the 2040 projection from the UK Department for Business, Energy & Industrial Strategy report on electricity generation costs to estimate the 2050 cost of CCGTs. Euro-Calliope v1.0 already relies on Hydropower capacity from the JRC Hydro-power database, which we update to version 7[44]. We also remove a scaling step, such that JRC data on pumped storage is used directly, rather than being scaled to fit capacities assumed by Geth et al.[45].

Table S7: Range of installed nuclear capacity in 2050 for subset of European countries in which some nuclear capacity is planned or under consideration. Countries given by their ISO3 codes.

		BGR	CZE	FIN	FRA	GBR	HUN	ROU	SVK
**Installed capacity (MW)**	*Min*	0	6230	0	22000	8900	2400	650	940
	Max	3200	7860	2750	58600	8900	2400	2650	3340

FILLING DATA GAPS

During data processing there are many cases where there are gaps for certain countries, years, end-uses, or energy carriers. Specific data filling can be found in the data processing workflow. The primary data filling methods we utilized are:

1. If not available in Eurostat or JRC IDEES, namely Switzerland, nationally published data has been used.
2. Where possible, gaps are filled using total sectoral energy demand. In years with data, an average contribution of each end use to total demand is calculated (e.g. X% of demand is for cooking); this average contribution is then applied to gaps (e.g. cooking demand in year Y = household demand in year Y * X%).
3. If end-use data is unavailable (e.g., for commercial and industrial heat demand), gaps are filled in at the energy consumption stage. The average relative contribution of each energy carrier to each end use is applied to all years without JRC-IDEES data but with Eurostat annual energy balance data.
4. If no data is available from the Eurostat annual energy balances, we take demand to be the average demand for that end-use for all years that we have data.
5. If no data is available at all for a country (e.g., we do not have cooking profiles for some eastern European countries), the average of data from the closest available neighbouring countries is used.

FINAL SECTOR-COUPLED ENERGY SYSTEM MODEL

In each of the aforementioned sectors, there exists some degree of electricity demand in today's energy system. The extent of this demand is quite limited in some sectors, e.g., 1.7 TWh in 2018 passenger road transport, but can be considerable in others, e.g., 670 TWh in 2018 building heat demand. To avoid double-counting demand, we remove existing heat and vehicle electricity end-use consumption from the electricity load curve.

Finally, although all abovementioned subsectors and energy carriers can be modelled in the Sector-Coupled Euro-Calliope, we have made some simplifications in the context of this study to ensure model tractability:

1. Road vehicles are grouped into *heavy* (heavy-duty vehicles and busses) and *light* (light-duty vehicles, motorcycles, and passenger cars), with electric vehicle plug-in profiles only applied to *light* vehicles.
2. Air-source and ground-source heat pumps are represented by a single technology, whose characteristics are a weighted average of the two main heat pump classes. The weighting is based on the ratio of air- to ground-source heat pump sales in 2016 and 2018, according to the European heat pump association.
3. The number of combined heat and power technologies has been reduced from six to three, by selecting only those technologies that are likely to be more prevalent in future according to expert opinion.
4. Industry feedstock demand for hydrogen and CO_2 can only be met by electrification in the model (electrolysis and direct air capture, respectively). We have therefore added this to industry electricity demand directly.

The final flow of energy carriers from supply to demand defined in Euro-Calliope is depicted in Figure S13.

Updates to the Calliope modelling framework

To represent all new sectors in Euro-Calliope, we have added mathematical constraints on top of the underlying Calliope modelling framework. New constraints include:

- Time varying technology capacity applied to heat pump heat capacity and electric vehicle charging capacity.
- Annual average capacity factor upper and lower bounds applied to nuclear technologies.
- Combined heat and power operating ranges, to capitalise on characteristics provided by the Danish Energy Agency technology database (C_b and C_v coefficients).
- Fixed demand share as a decision variable, applied to the share of heat and transport supply technologies. With this constraint we are able to model the operation of disaggregated technologies, e.g., household heat supply. For instance, if 60% of households invest in heat pumps then only approximately 60% of heat demand can be met by heat pumps in each hour.
- Demands can be time independent. This allows e.g., kerosene for aviation to be generated according to any profile, provided there is sufficient kerosene generated by the end of the model period.

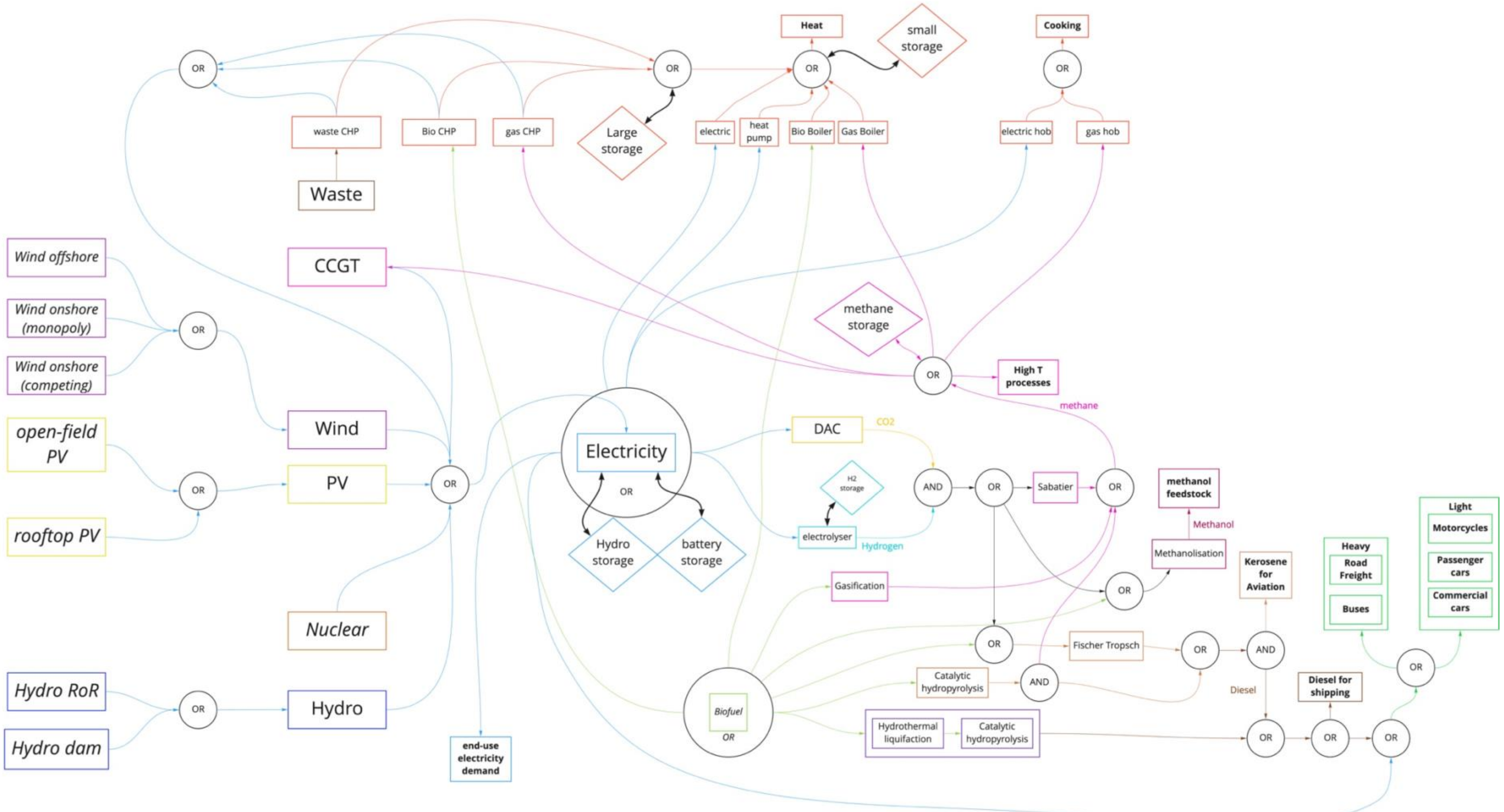

Figure S13: Flow chart of the final Euro-Calliope sector-coupled model, including supply technologies, energy carriers, and demand sources. AND and OR icons in the flow indicate when an in/output is a combination (AND) or choice (OR) of out/input. Where energy enters the systems from an external resource, the carrier is given in italics (e.g. Waste). Storage technologies are depicted as diamonds. Bold text refers to demands, where energy exits the system. Flow line colours are related to the energy carrier flowing along that line.

SUPPLEMENTAL ITEMS

NOTE S2: SENSITIVITY ANALYSES

The aim of the sensitivity analysis (SA) that we describe in this section is to assess how sensitive our conclusions are to input parameters that we deem most likely to change in reality. To do this we use the one-at-a-time (OAT) approach, which is commonly used in computationally intensive models that do not lend themselves to a global SA[46]. Unlike traditional energy modelling efforts which also undertake OAT SA (e.g. [47–49]), our conclusions are concerned with the diversity of options and the trade-offs between those options, and not with specific quantities of individual technologies or costs of a small set of solutions. Therefore, our SA focusses on assessing whether changes in input parameters produce significant deviations from our conclusions that the option space and trade-offs are general features of the European energy system.
Because of its spatial and temporal detail, the sector-coupled Euro-Calliope model is computationally intensive (12 to 20 hours needed to compute one SPORE, with limited scope for parallelisation). Therefore, we undertake an SA which balances the need to critically reflect on the modelling process with the computationally intense nature of our work. We limit ourselves to only three input parameters/parameter groups: weather year, annual demand projections, and cost relaxation. The setup and results of these analyses is described in the following sections.

WEATHER YEAR

The model is run for the baseline year of 2018, which we use to derive service demands and variable renewable technology resource availability. In our previous work, we have shown that the choice of weather year has a greater impact on the option space of SPORES than other key uncertain parameters (demand and technology costs)[41]. However, it is impractical to run SPORES for all weather years, so we focus on the structure of the cost-optimal solutions, to see whether there are any spatio-temporal dynamics of supply and demand in the years 2010 - 2017 that are so different to 2018 as to suggest the need for a drastically different energy system configuration. That is, we check to ensure that there is no overfitting in the context of our conclusions. We do not find evidence for this.

The result of this comparison is shown in Figure S14 and Figure S15. As can be seen, the cost optimal results are within the bounds of the SPORE results for the weather year 2018, whether for absolute capacities or our nine metrics of interest. There is often very little spread in capacity between the years, particularly for the highest capacity technologies (AC transmission, PV, wind, electrolysis). CCGT and battery storage capacity does vary more noticeably between weather years, suggesting that weather variability is more pronounced in 2015 and 2016, requiring additional flexibility. Across our metrics, differences are also generally very small, with biofuel utilisation varying most prominently. In addition, the system cost of the different systems varies by a maximum of 3.5% compared to the 2018 optimal system cost; i.e., significantly less than the cost relaxation. Since these variations between weather years are noticeably lower than the range of SPORES, with no weather year metric or capacity sitting outside the range of SPORES, we do not deem there to be significant weather year overfitting.

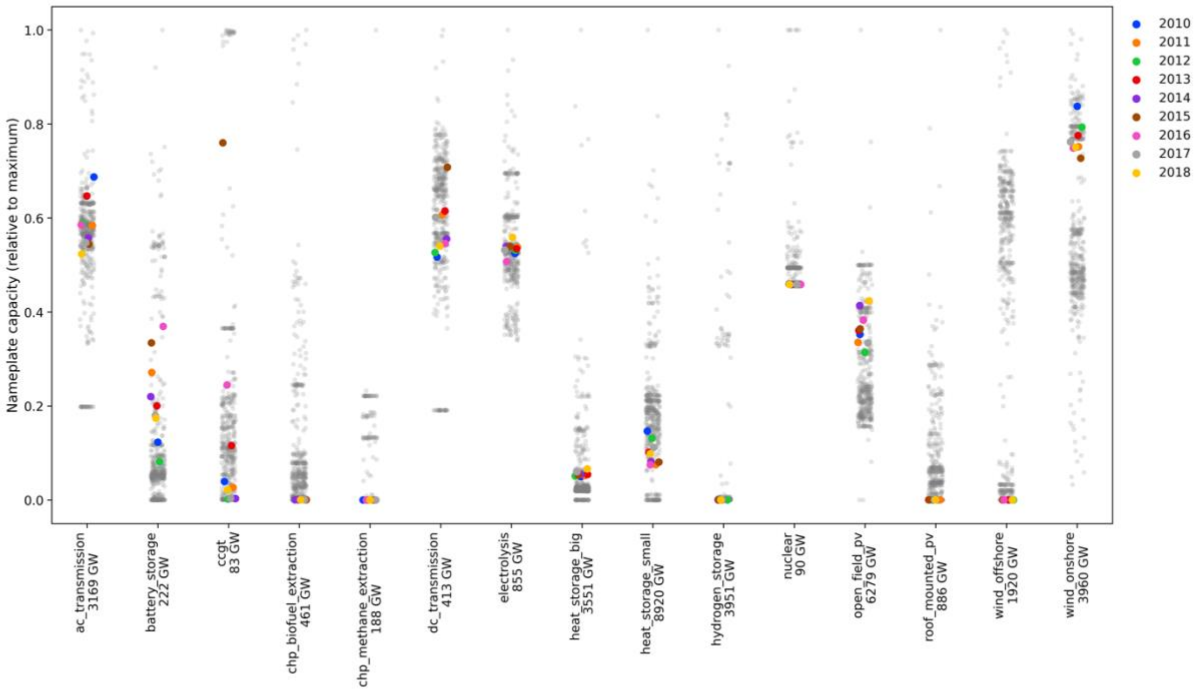

Figure S14: Technology nameplate capacities in all 10% relaxation SPORES (441 results; light grey markers) and in all cost-optimal results across the weather years 2010 – 2018 (coloured markers). SPORES are based on the 2018 weather year. Technologies include those that produce, transmit, or store energy. Storage capacities refer to the charge/discharge capacity. Results are shown scaled to the maximum capacity for each technology, across all results. The value of this maximum capacity is given next to the technology name on the x axis.

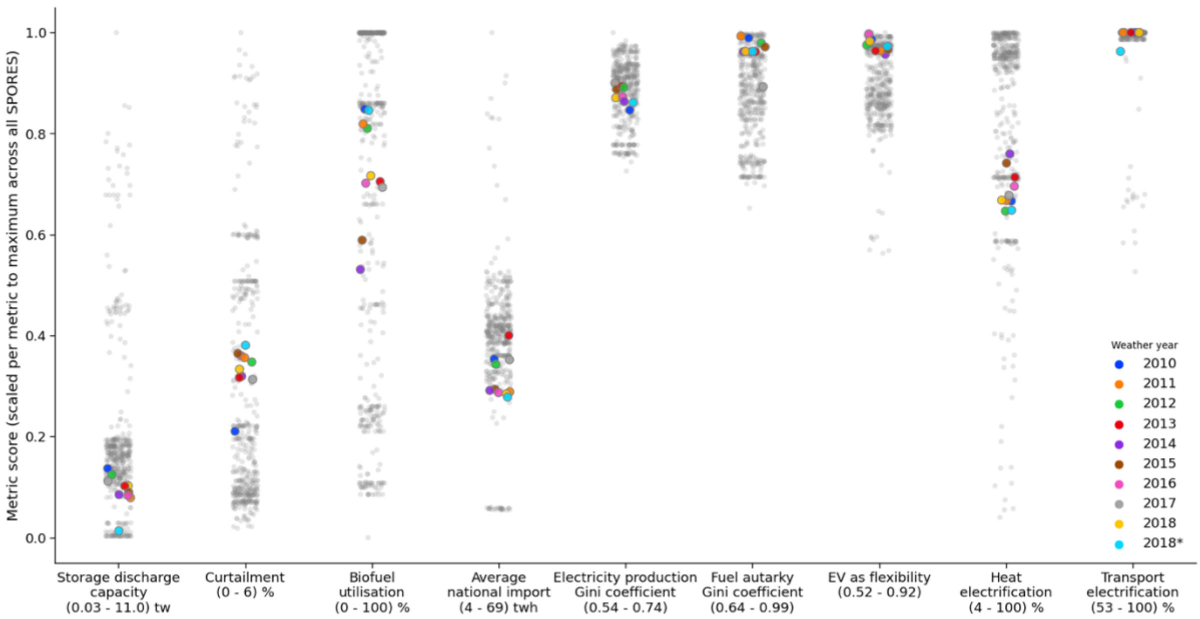

Figure S15: Scaled metric scores in all 10% relaxation SPORES (441 results; light grey markers) and in all cost-optimal results across the weather years 2010 – 2018 (coloured markers) and for the weather year 2018 with projected demands (2018). SPORES are based on the 2018 weather year. Absolute metric ranges are given in brackets underneath each metric name on the x-axis.*

DEMAND PROJECTIONS

In the base model, we use 2018 service demands derived from the pre-processing steps described in Note S1. We conduct this sensitivity analysis to check whether key features of the SPORES option space is affected significantly by our choice to use 2018 service demands directly.

We use simulated increases in national demands for end-use services from the models DESTINEE[17] and HEB[50], according to scenarios aimed at pan-European carbon-neutrality by 2050. We do not use the absolute 2050 demands directly, but scale Euro-Calliope service demands according to the simulated increases from 2015 to 2050. Scaled demands are described further in Table S8. We do not have data on demand scales for all 35 modelled countries; where no data is available, average scales from neighbouring countries have been used. Increases in demand are seen in industry and transport, due to the simulated effects of increases in population, average income, and value added. Building-level demands also include these effects, but they are outweighed by simulated reductions of demand intensity, due to appliance efficiency improvements and deep building retrofit. Final model demands after applying scaling factors are available in the data files published with this study: DOI:10.5281/zenodo.6546817.

We optimised the scaled demand model for the 2018 weather year, maintaining the shape of timeseries profiles from this weather year and only scaling the annual magnitudes of demand. We also produced 73 SPORES, focussing on producing system configurations with greatest technological differences, rather than spatial ones. As expected, the cost-optimal energy system differs when using scaled demands; the objective function value increases by 20%. Yet, the metric scores of this cost-optimal solution is very similar to that given by other cost optimal solutions (Figure S15 – 2018*). We also find that the option space exposed by our SPORES remains roughly the same, whether using 2018 end-use demands directly or modelling with projected demand increases. For our nine metrics defined in Table 1, Figure S16 shows the similarity in the extent of metric values between the two demand scenarios. Although SPORES are concentrated in different places between the two scenarios, the range they cover is similar. This means that irrespective of the demand scenario used, system designs exist that enable similar levels of maximisation/minimisation of particular metrics.

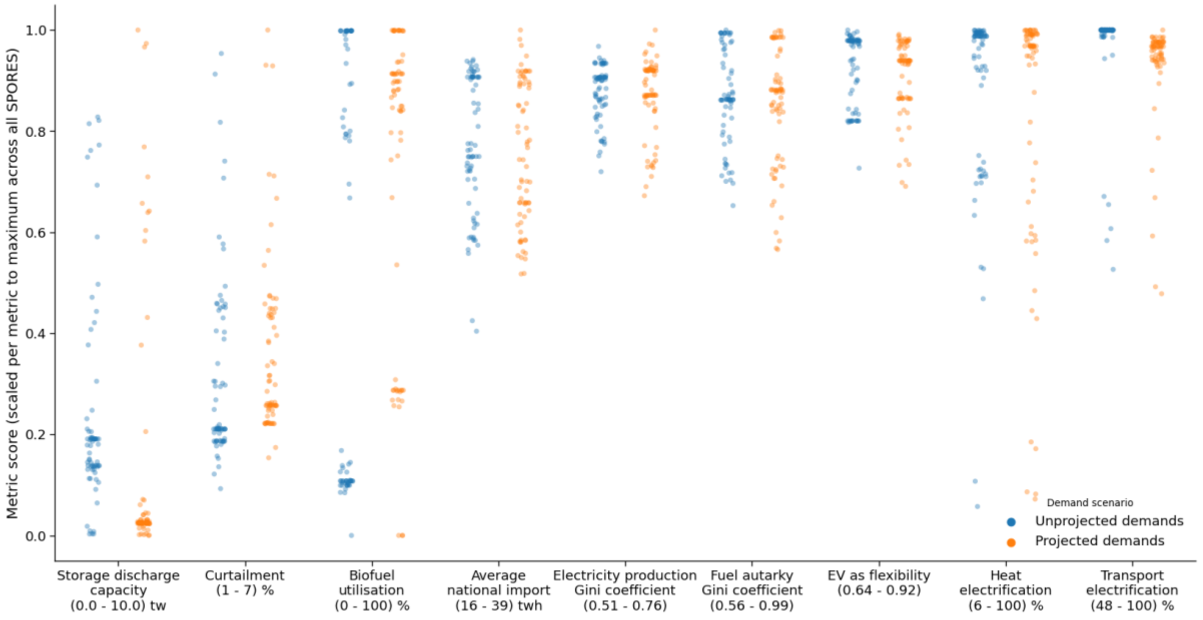

Figure S16: Impact of projected demands, summarized in Table S8, on the range of SPORES across the nine high-level metrics used to understand the option space in this study. Absolute metric ranges are given in brackets underneath each metric name on the x-axis.

Table S8: Summary of data sources to scale demands and the impact on modelled demands of including these scales.

Sector	Simulated data	Demand scenario source	Increase in total demand, relative to baseline 2018 model (%)
Industry	Sub-sector value added	DESTINEE	1.39
Road transport	Number of vehicles and total vehicle kms	DESTINEE	1.27
Aviation & shipping	Total vehicle kms	DESTINEE	1.62
Rail	Total vehicle kms	DESTINEE	1.59
Building appliances & cooling	Electricity consumption	DESTINEE	0.77
Building heat	Energy consumption	HEB	0.27

Cost relaxation

For the baseline year 2018, we also test a subset of SPORE runs with 5% and 15% relaxations. We generated 120 SPORES in total per sensitivity run. We compare the results for the equivalent runs in the baseline (10% relaxation) run. These SPORES focus on excluding specific technology groups whilst exploring spatial diversity of primary electricity supply. Here, we compare the range of high-level metrics across SPORES for the different relaxations, using the same metrics as shown in Figure 3 of the main text.

The result of this comparison is shown in Figure S17. As expected, the range of metric values increases with increasing cost relaxation. In some cases, a relaxation of 5% does not sit within the same range as achieved with a relaxation of 15%. For instance, the lowest average national import metric score is 0.48 for a relaxation of 5% and 0.53 for a relaxation of 15%. A relaxation of 15% could unlock metric scores which differ from our baseline 10% relaxation when they are already showing a significant difference in this subset of results; that is, transport electrification, biofuel utilisation, and EV as a flexibility source (through smart charging). If these metrics are of particular interest to minimise, a full set of SPORES at a 15% cost relaxation would need to be generated.

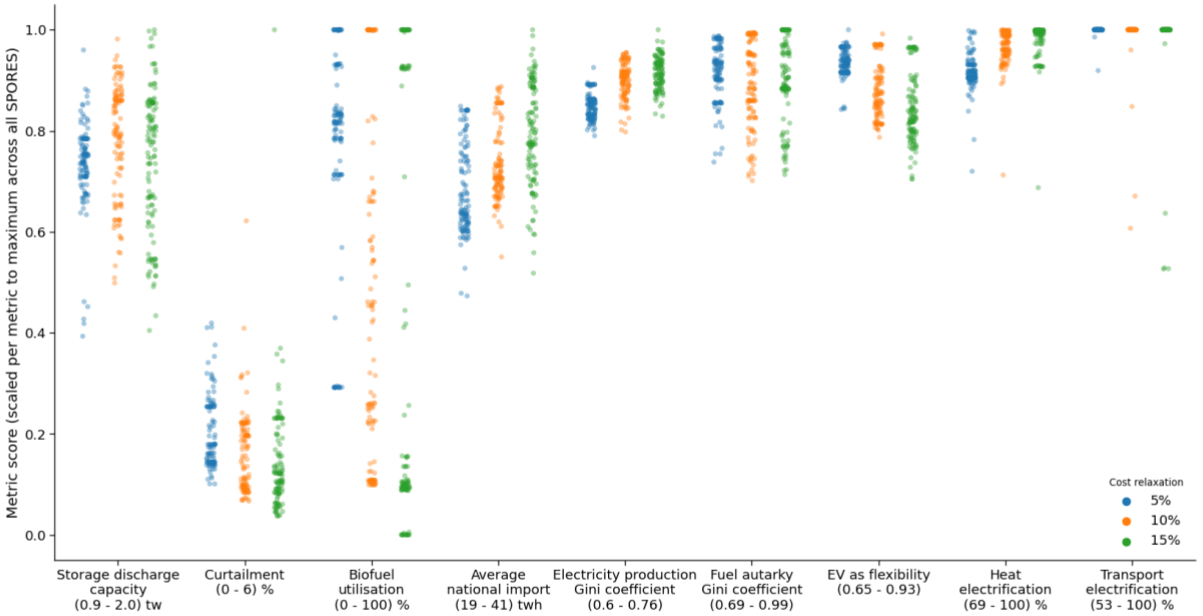

Figure S17: Comparison of high level metric scores, scaled to the maximum value across all SPORES. 120 SPORES are shown, including all those for which there is a result in all cost relaxations. A description of the metrics can be found in the main text (Table 1). Absolute metric ranges are given in brackets underneath each metric name on the x-axis.

Note S3: Seasonal balancing mechanisms

By comparing monthly energy production across all 441 baseline SPORES, we aim to identify the key sub-seasonal to seasonal balancing mechanisms across SPORES. Figure S18 shows that PV and wind monthly variations in production are most strongly (anti)correlated with monthly variations of demands. Indeed, strong dependence on wind production goes a great length to balance monthly demand variations across seasons. Dispatchable energy supply (biofuels, waste, and CCGT) can either help balance seasonal demand variations (positive correlation) or balance other flows in the system (negative correlation). For biofuel and waste supply, this choice on how much to correlate/anticorrelate with demand exists irrespective of the dependence of the SPORE on wind or solar production. Conversely, CCGT operation is used more often to balance seasonally in SPORES with high PV dependence, and used to balance on other timescales in SPORES with high wind dependence. SPORES with high dependence on PV production also rely on hydrogen (and, subsequently, synthetic fuel) production and storage. This leads to the strongest anticorrelation of hydrogen production with end-use demands, as fuel is produced predominantly in the summer months. Hydropower dependence can change seasonally, by controlling the output of reservoirs and use of pumped storage. Again, the strongest seasonal (anti)correlations are at the extremes of wind/PV production: high PV production means high dependence on reservoirs to follow seasonal demands, but high dependence on pumped storage to balance PV supply; high wind production means low dependence on reservoirs to follow seasonal demands, but (slightly) positive correlation between demand and pumped hydro output. Methane cavern storage acts inversely to pumped hydro storage.

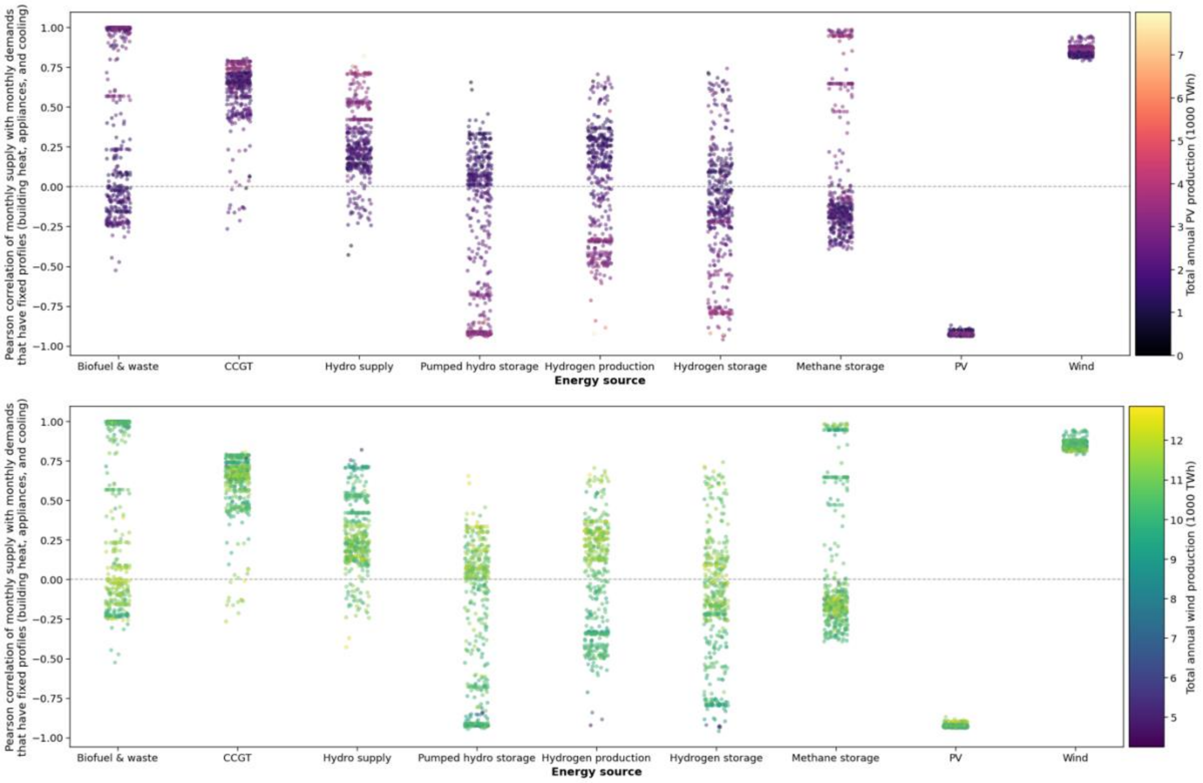

Figure S18: Pearson correlation of monthly total European production with demand across all 441 baseline SPORES, with each SPORE highlighted according to total annual European PV (top) and wind (bottom) electricity generation in that SPORE. A positive correlation indicates that a large fraction of the monthly variation in demand is matched by equal levels of monthly variation of supply. This means that matching on a monthly level without flexibility mechanisms is relatively easy. We have only selected for comparison those production technologies that can enable seasonal balancing: 1. Biofuel and municipal waste supplies, for use in direct combustion to meet end-use demands or to produce intermediate fuels; 2.combined cycle gas turbine (CCGT) electricity output; 3. reservoir and run-of-river hydropower electricity supply (Hydro supply); 4. Pumped hydro storage electricity output; 5. electricity consumed to produce hydrogen by electrolysis (Hydrogen production); 6. Hydrogen storage tank output; 7. Methane cavern storage output; 8. rooftop and open-field PV electricity production; 9. onshore and offshore wind electricity production. Only fixed timeseries demands are used in the correlation, which includes the base electricity demand (building appliances & cooling, rail, and industry processes) and building heat demands. Fuel and transport distance service demands are balanced on an annual basis, so are not included.

NOTE S4: LINKED SPORES METRIC VALUES

The following figures (S19 to S26) are the same as Figure 3a, but each highlights a different set of linked SPORES values. In the main text, linked SPORES are highlighted according to those which fall within the lowest 15 percentage points (+15pp) of the biofuel utilisation metric. In these additional figures, the linked +15pp SPORES of the remaining eight metrics are shown.

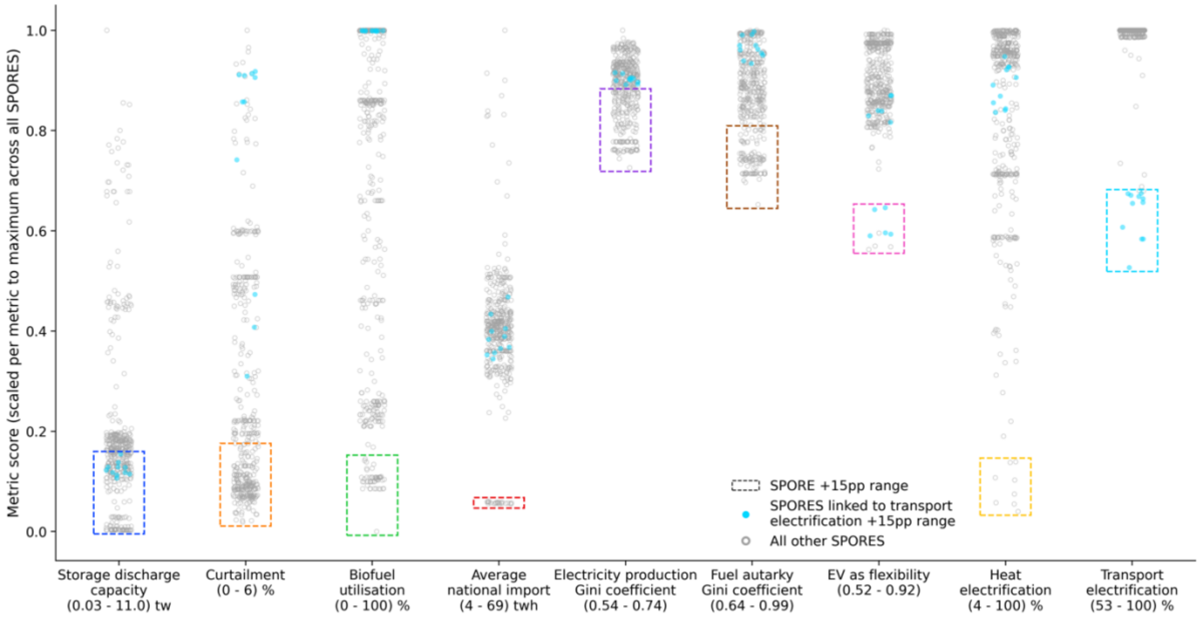

Figure S19: As with Figure 3a in the main text, but with SPORES linked to the "transport electrification" metric +15pp range highlighted.

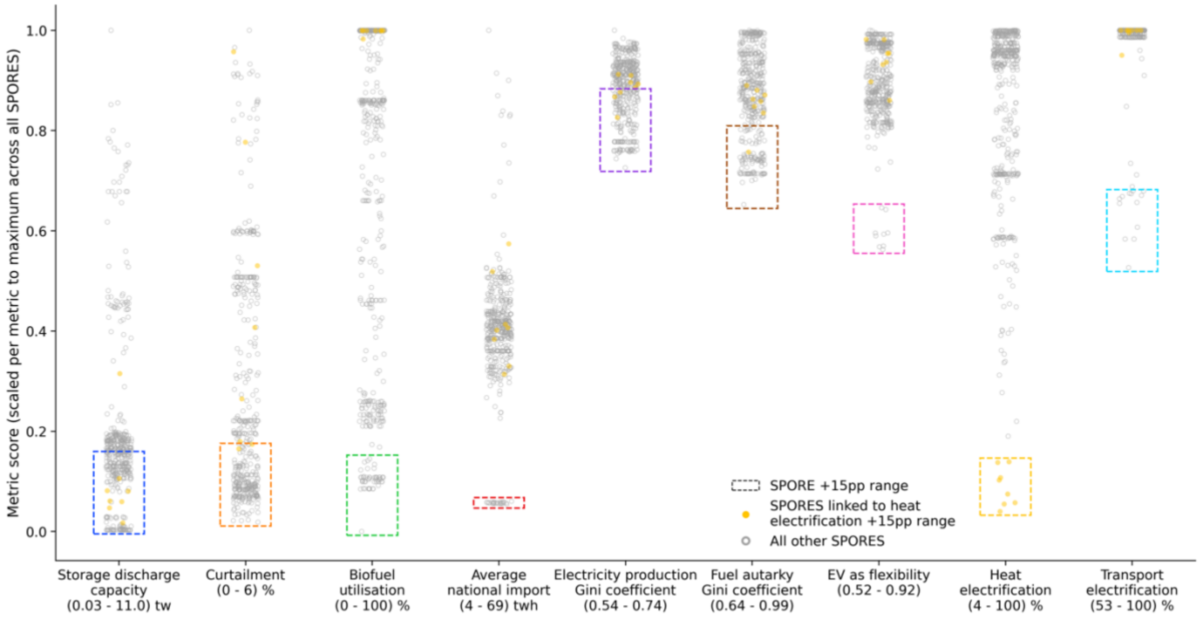

Figure S20: As with Figure 3a in the main text, but with SPORES linked to the "heat electrification" metric +15pp range highlighted.

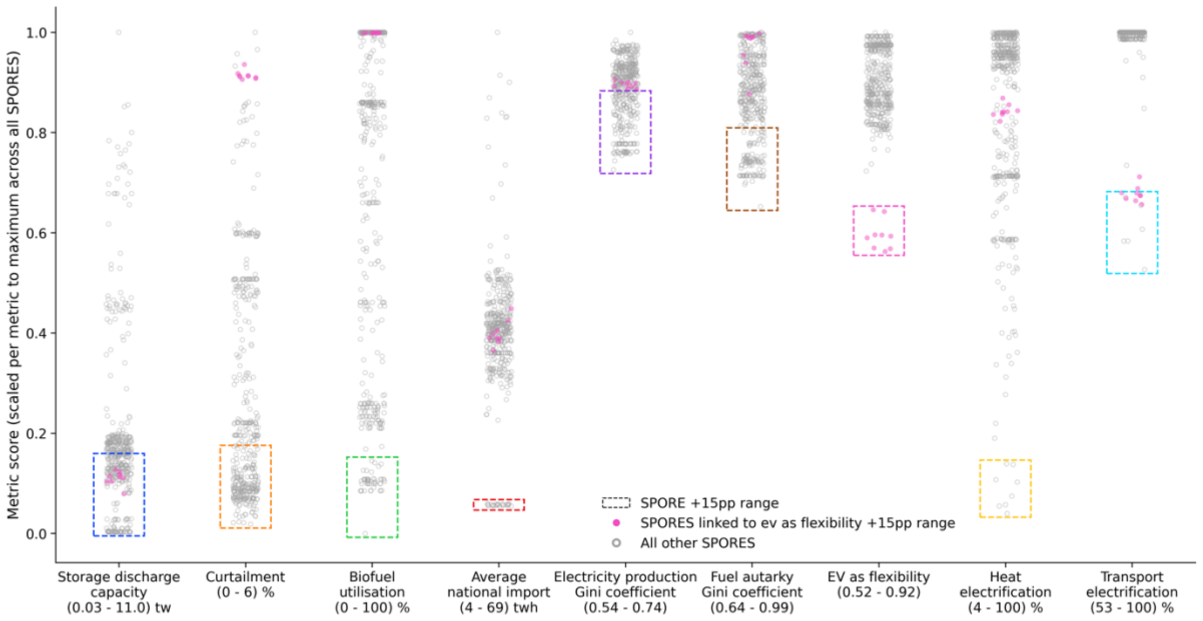

Figure S21: As with Figure 3a in the main text, but with SPORES linked to the "EV as flexibility" metric +15pp range highlighted.

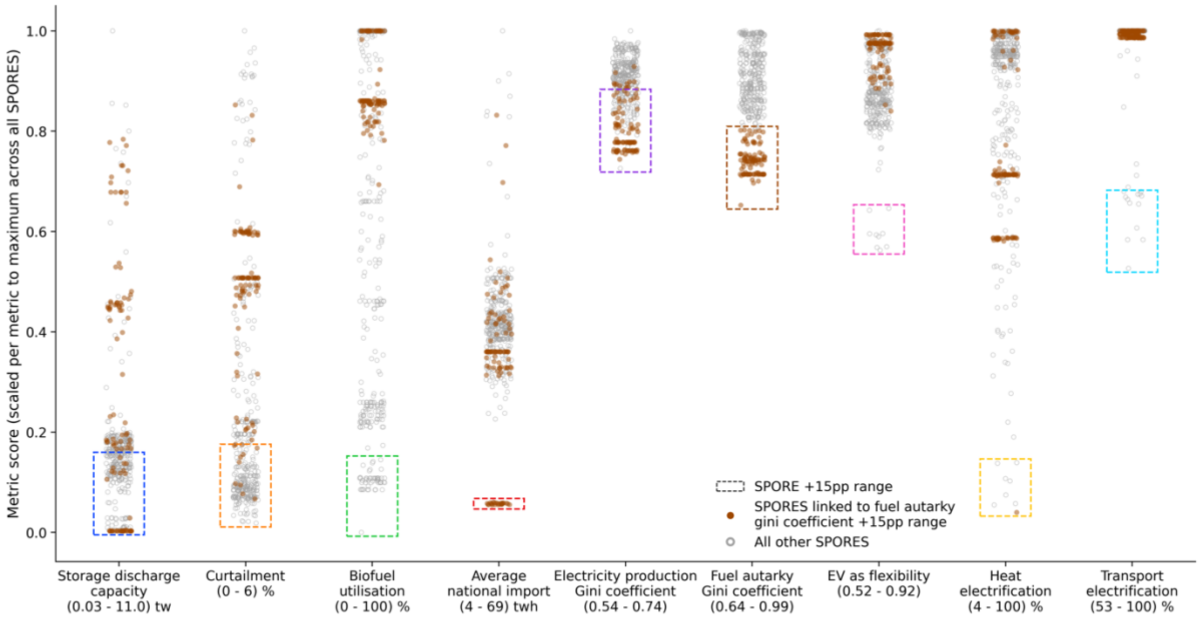

Figure S22: As with Figure 3a in the main text, but with SPORES linked to the "Fuel autarky gini coefficient" metric +15pp range highlighted.

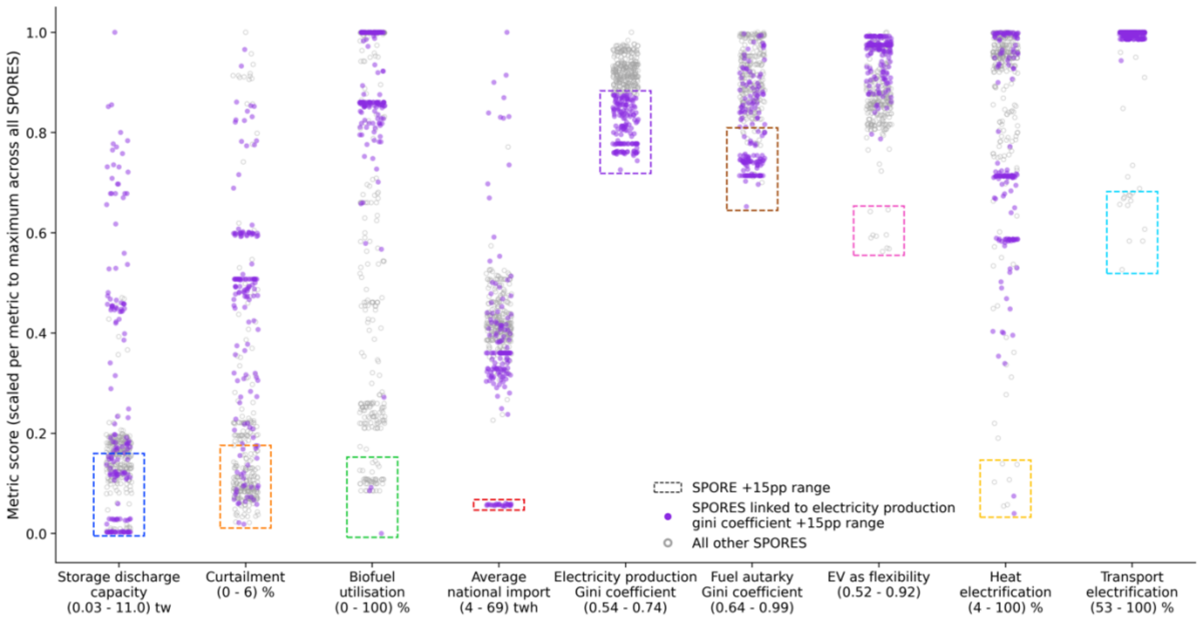

Figure S23: As with Figure 3a in the main text, but with SPORES linked to the "Electricity production gini coefficient" metric +15pp range highlighted.

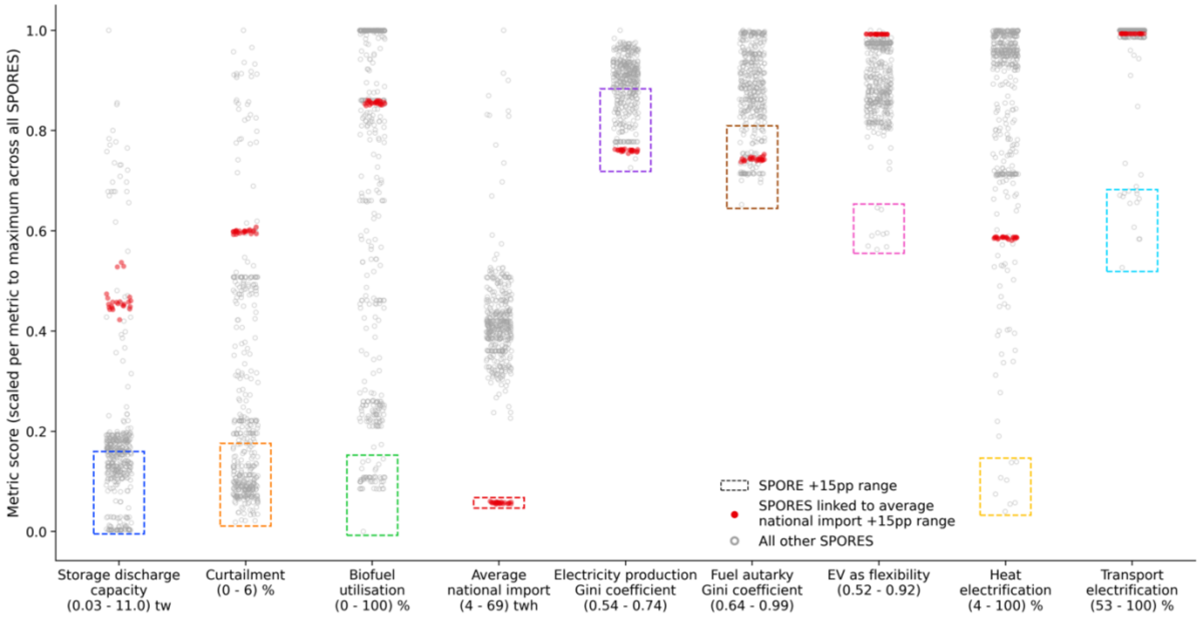

Figure S24: As with Figure 3a in the main text, but with SPORES linked to the "Average national import" metric +15pp range highlighted.

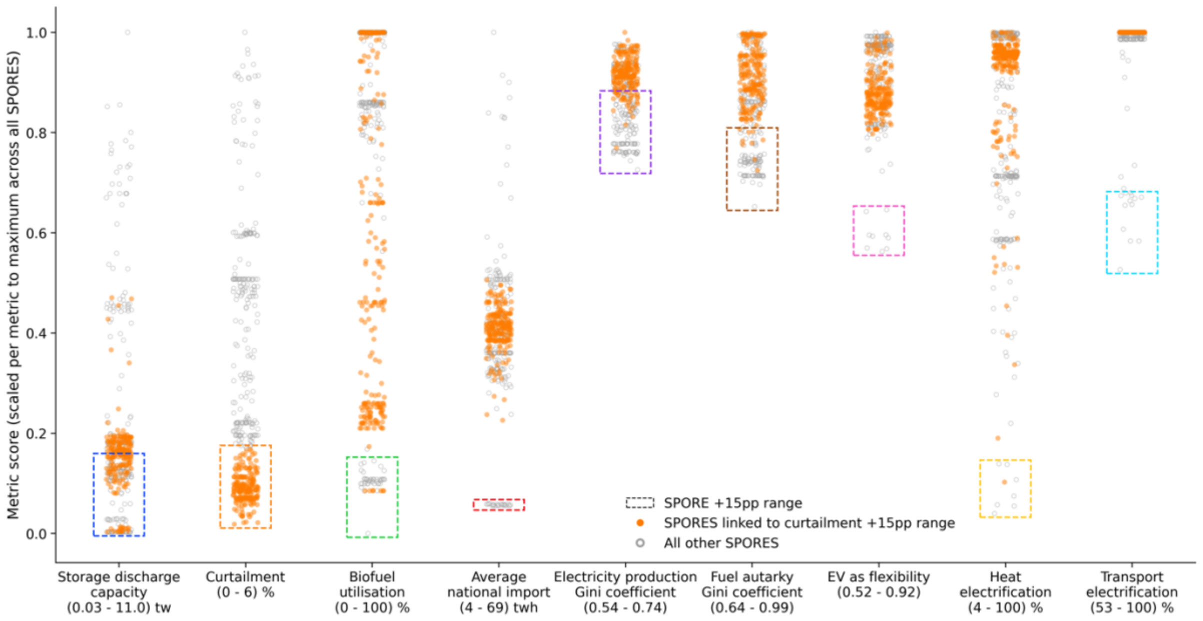

Figure S25: As with Figure 3a in the main text, but with SPORES linked to the "Curtailment" metric +15pp range highlighted.

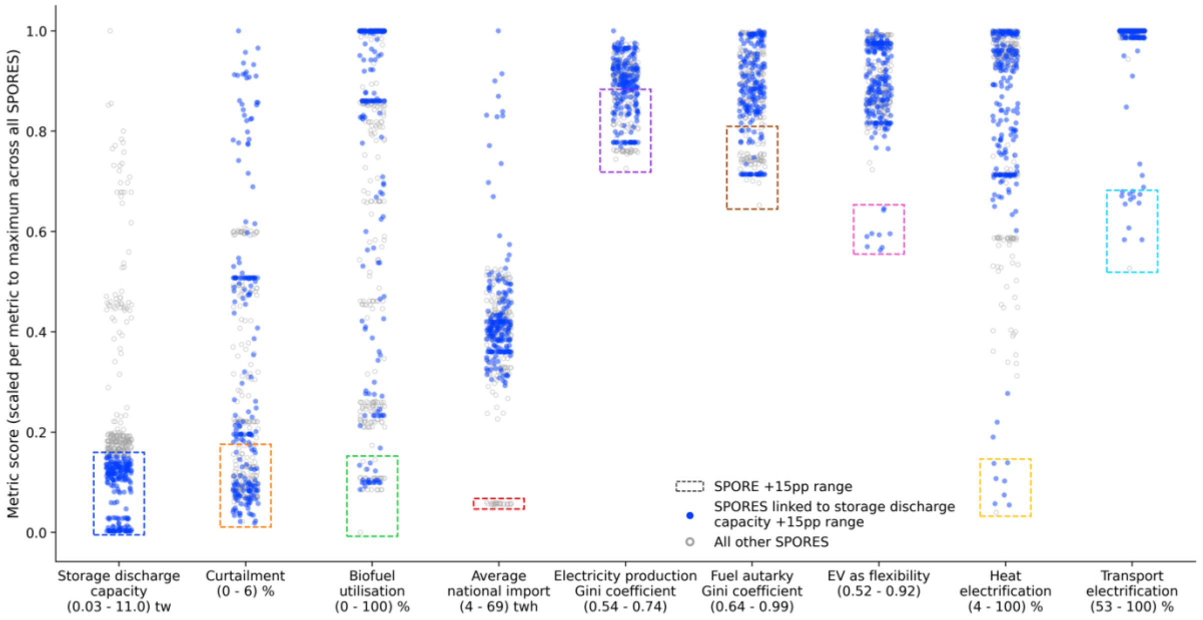

Figure S26: As with Figure 3a in the main text, but with SPORES linked to the "Storage discharge capacity" metric +15pp range highlighted.

Supplemental References

1. Tröndle, T. (2020). Euro-Calliope: pre-built models. https://zenodo.org/record/3949553.

2. Tröndle, T., Lilliestam, J., Marelli, S., and Pfenninger, S. (2020). Trade-Offs between Geographic Scale, Cost, and Infrastructure Requirements for Fully Renewable Electricity in Europe. Joule *4*, 1929–1948.

3. Mantzos, L., Wiesenthal, T., Matei, N.A., Tchung-Ming, S., Rozsai, M., Russ, P., and Ramirez, A.S. (2017). JRC-IDEES: Integrated Database of the European Energy Sector: Methodological Note (Joint Research Centre (Seville site)).

4. Kanellopoulos K., De Felice M., Hidalgo Gonzalez I., and Bocin A. (2019). JRC Open Power Plants Database (JRC-PPDB-OPEN). https://zenodo.org/record/3574566.

5. Ruhnau, O., Hirth, L., and Praktiknjo, A. (2019). Time series of heat demand and heat pump efficiency for energy system modeling. Sci Data *6*, 1–10.

6. Lombardi, F., Balderrama, S., Quoilin, S., and Colombo, E. (2019). Generating high-resolution multi-energy load profiles for remote areas with an open-source stochastic model. Energy *177*, 433–444.

7. Lombardi, F., Rocco, M.V., and Colombo, E. (2019). A multi-layer energy modelling methodology to assess the impact of heat-electricity integration strategies: The case of the residential cooking sector in Italy. Energy *170*, 1249–1260.

8. The Danish Energy Agency and Energinet (2016). Technology Data: Heating installations (The Danish Energy Agency).

9. Nouvel, R., Cotrado Sehgelmeble, M., and Pietruschka, D. (2015). European Mapping of Seasonal Performances of Air-source and Geothermal Heat Pumps for Residential Applications. In.

10. Staffell, I., Brett, D., Brandon, N., and Hawkes, A. (2012). A review of domestic heat pumps. Energy Environ. Sci. *5*, 9291–9306.

11. Gelaro, R., McCarty, W., Suárez, M.J., Todling, R., Molod, A., Takacs, L., Randles, C.A., Darmenov, A., Bosilovich, M.G., Reichle, R., et al. (2017). The Modern-Era Retrospective Analysis for Research and Applications, Version 2 (MERRA-2). Journal of Climate *30*, 5419–5454.

12. The Danish Energy Agency (2019). Technology Data - Renewable Fuels.

13. Chandrasekaran, S.R., Hopke, P.K., Newtown, M., and Hurlbut, A. (2013). Residential-Scale Biomass Boiler Emissions and Efficiency Characterization for Several Fuels. Energy Fuels *27*, 4840–4849.

14. Mermoud, F., Haroutunian, A., Faessler, J., and Lachal, B.M. (2015). Impact of load variations on wood boiler efficiency and emissions: in-situ monitoring of two boilers (2 MW and 0.65 MW) supplying a district heating system. Archives des Sciences *68*, 27–38.

15. Karunanithy, C., and Shafer, K. (2016). Heat transfer characteristics and cooking efficiency of different sauce pans on various cooktops. Applied Thermal Engineering *93*, 1202–1215.

16. Ramanathan, R., and Ganesh, L.S. (1994). A multi-objective analysis of cooking-energy alternatives. Energy *19*, 469–478.

17. Boßmann, T., and Staffell, I. (2015). The shape of future electricity demand: Exploring load curves in 2050s Germany and Britain. Energy *90*, 1317–1333.

18. Mangipinto, A., Lombardi, F., Sanvito, F.D., Pavičević, M., Quoilin, S., and Colombo, E. (2022). Impact of mass-scale deployment of electric vehicles and benefits of smart charging across all European countries. Applied Energy *312*, 118676.

19. The European Council for Automotive R&D (2019). Battery requirements for future automotive applications (EUCAR).

20. Fleiter, T., Elsland, R., Herbst, A., Manz, P., Popovski, E., Rehfeldt, M., Reiter, U., Catenazzi, G., Jakob, M., Harmsen, R., et al. (2017). Heat Roadmap Europe: Baseline scenario of the heating and cooling demand in buildings and industry in the 14 MSs until 2050.

21. Naegler, T., Simon, S., Klein, M., and Gils, H.C. (2015). Quantification of the European industrial heat demand by branch and temperature level. International Journal of Energy Research *39*, 2019–2030.

22. Rehfeldt, M., Fleiter, T., and Toro, F. (2018). A bottom-up estimation of the heating and cooling demand in European industry. Energy Efficiency *11*, 1057–1082.

23. Reiter, U., Catenazzi, G., Jakob, M., Naegeli, C., Fleiter, T., Steinbach, J., Ragwitz, M., Arens, M., Aydemir, A., Elsland, R., et al. (2016). Mapping and analyses of the current and future (2020-2030) heating/cooling fuel deployment (fossil/renewables) - Work package 1: Final energy consumption for the year 2012 (Fraunhofer Institute for Systems and Innovation Research (ISI)).

24. Madeddu, S., Ueckerdt, F., Pehl, M., Peterseim, J., Lord, M., Kumar, K.A., Krüger, C., and Luderer, G. (2020). The CO2 reduction potential for the European industry via direct electrification of heat supply (power-to-heat). Environ. Res. Lett.

25. Suopajärvi, H., Umeki, K., Mousa, E., Hedayati, A., Romar, H., Kemppainen, A., Wang, C., Phounglamcheik, A., Tuomikoski, S., Norberg, N., et al. (2018). Use of biomass in integrated steelmaking – Status quo, future needs and comparison to other low-CO2 steel production technologies. Applied Energy *213*, 384–407.

26. Mandova, H., Leduc, S., Wang, C., Wetterlund, E., Patrizio, P., Gale, W., and Kraxner, F. (2018). Possibilities for CO2 emission reduction using biomass in European integrated steel plants. Biomass and Bioenergy *115*, 231–243.

27. Material Economics (2019). Industrial transformation 2050-Pathways to net-zero emissions from EU heavy industry.

28. Word Steel Association (2019). World Steel in Figures 2019 (Brussels: Word Steel Association).

29. Mathieson, J., Rogers, H., Somerville, M., Ridgeway, P., and Jahanshahi, S. (2011). Use of biomass in the iron and steel industry - An Australian perspective. In.

30. Suopajärvi, H., Kemppainen, A., Haapakangas, J., and Fabritius, T. (2017). Extensive review of the opportunities to use biomass-based fuels in iron and steelmaking processes. Journal of Cleaner Production *148*, 709–734.

31. Vogl, V., Åhman, M., and Nilsson, L.J. (2018). Assessment of hydrogen direct reduction for fossil-free steelmaking. Journal of Cleaner Production *203*, 736–745.

32. Fischedick, M., Marzinkowski, J., Winzer, P., and Weigel, M. (2014). Techno-economic evaluation of innovative steel production technologies. Journal of Cleaner Production *84*, 563–580.

33. Worrell, E., Price, L., Neelis, M., Galitsky, C., and Zhou, N. (2007). World best practice energy intensity values for selected industrial sectors.

34. International Energy Agency (2018). The Future of Petrochemicals: Towards More Sustainable Plastics and Fertilisers (IEA Publications France).

35. Bazzanella, A., and Ausfelder, F. (2017). Low carbon energy and feedstock for the European chemical industry (DECHEMA, Gesellschaft für Chemische Technik und Biotechnologie eV).

36. Saebea, D., Ruengrit, P., Arpornwichanop, A., and Patcharavorachot, Y. (2020). Gasification of plastic waste for synthesis gas production. Energy Reports *6*, 202–207.

37. Liljenström, C., and Finnveden, G. (2015). Data for separate collection and recycling of dry recyclable materials (KTH Royal Institute of Technology).

38. AISBL, P. (2019). Plastics–the Facts 2018. Information accessed at http://www.-plasticseurope. org/application.

39. Boulamanti, A., and Moya, J.A. (2017). Production costs of the chemical industry in the EU and other countries: Ammonia, methanol and light olefins. Renewable and Sustainable Energy Reviews *68*, 1205–1212.

40. Fasihi, M., Efimova, O., and Breyer, C. (2019). Techno-economic assessment of CO2 direct air capture plants. Journal of Cleaner Production *224*, 957–980.

41. Lombardi, F., Pickering, B., Colombo, E., and Pfenninger, S. (2020). Policy Decision Support for Renewables Deployment through Spatially Explicit Practically Optimal Alternatives. Joule *0*.

42. Anderski, T., Surmann, Y., Stemmer, S., Grisey, N., Momot, E., Leger, A.-C., Betraoui, B., and van Roy, P. (2014). European cluster model of the Pan-European transmission grid (e-HIGHWAY 2050).

43. Department for Business, Energy & Industrial Strategy (2019). Sub-national Electricity and Gas Consumption: Regional and Local Authority, Great Britain, 2018.

44. De Felice, M., and Kavvadias, K. (2020). energy-modelling-toolkit/hydro-power-database: JRC Hydro-power database - release 07 (Zenodo).

45. Geth, F., Brijs, T., Kathan, J., Driesen, J., and Belmans, R. (2015). An overview of large-scale stationary electricity storage plants in Europe: Current status and new developments. Renewable and Sustainable Energy Reviews *52*, 1212–1227.

46. Pianosi, F., Beven, K., Freer, J., Hall, J.W., Rougier, J., Stephenson, D.B., and Wagener, T. (2016). Sensitivity analysis of environmental models: A systematic review with practical workflow. Environmental Modelling & Software *79*, 214–232.

47. Pietzcker, R.C., Osorio, S., and Rodrigues, R. (2021). Tightening EU ETS targets in line with the European Green Deal: Impacts on the decarbonization of the EU power sector. Applied Energy *293*, 116914.

48. Stöckl, F., Schill, W.-P., and Zerrahn, A. (2021). Optimal supply chains and power sector benefits of green hydrogen. Sci Rep *11*, 14191.

49. Zeyringer, M., Price, J., Fais, B., Li, P.-H., and Sharp, E. (2018). Designing low-carbon power systems for Great Britain in 2050 that are robust to the spatiotemporal and inter-annual variability of weather. Nat Energy *3*, 395–403.

50. Güneralp, B., Zhou, Y., Ürge-Vorsatz, D., Gupta, M., Yu, S., Patel, P.L., Fragkias, M., Li, X., and Seto, K.C. (2017). Global scenarios of urban density and its impacts on building energy use through 2050. Proc Natl Acad Sci U S A *114*, 8945–8950.
