# Supplementary material for: Diversity of options to eliminate fossil fuels and reach carbon neutrality across the entire European energy system
Source: Joule. 2022 Jun 15;6(6):1253–76. doi: 10.1016/j.joule.2022.05.009 (PMC9220955; doi:10.1016/j.joule.2022.05.009)
Supplement: Document S2. Article plus supplemental information [file mmc2.pdf]

Joule

CellPress
OPEN ACCESS

Article

Diversity of options to eliminate fossil fuels and reach carbon neutrality across the entire European energy system

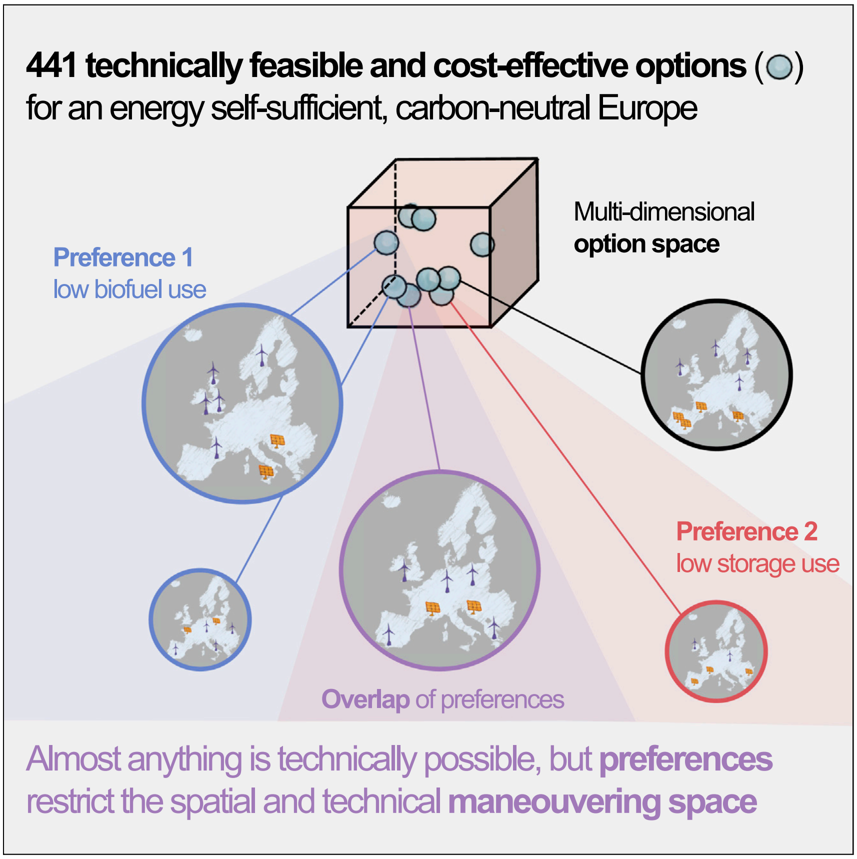

Many technical options exist to achieve carbon neutrality across all energy sectors and to completely eliminate fossil fuel imports in Europe. The energy system can be designed to rely to a varying extent on wind, solar, biofuels, and other technologies, and the infrastructure can be located in different regions. However, imposing specific preferences, such as biofuel dependence, reduces the maneuvering space for other decisions. In all cases, the scale of the transformation remains enormous and requires acceleration.

Bryn Pickering, Francesco Lombardi, Stefan Pfenninger

bryn.pickering@usys.ethz.ch

Highlights

Many cost-effective options exist for an energy self-sufficient, carbon-neutral Europe

A variety of spatial configurations are valid, and specific regions can be prioritized

Although firm capacity is not a must have, limiting it reduces the maneuvering space

For example, low bioenergy requires electrified heat and controlled vehicle charging

Pickering et al., Joule 6, 1253–1276
June 15, 2022 © 2022 The Author(s). Published by Elsevier Inc.
https://doi.org/10.1016/j.joule.2022.05.009

Joule

CellPress OPEN ACCESS

Article

Diversity of options to eliminate fossil fuels and reach carbon neutrality across the entire European energy system

Bryn Pickering,[1,3,*] Francesco Lombardi,[2] and Stefan Pfenninger[2]

SUMMARY

Disagreements persist on how to design a self-sufficient, carbon-neutral European energy system. To explore the diversity of design options, we develop a high-resolution model of the entire European energy system and produce 441 technically feasible system designs that are within 10% of the optimal economic cost. We show that a wide range of systems based on renewable energy are feasible, with no need to import energy from outside Europe. Model solutions reveal considerable flexibility in the choice and geographical distribution of new infrastructure across the continent. Balanced renewable energy supply can be achieved either with or without mechanisms such as biofuel use, curtailment, and expansion of the electricity network. Trade-offs emerge once specific preferences are imposed. Low biofuel use, for example, requires heat electrification and controlled vehicle charging. This exploration of the impact of preferences on system design options is vital to inform urgent, politically difficult decisions for eliminating fossil fuel imports and achieving European carbon neutrality.

Context & scale

Most studies to reach European carbon -neutrality focus on one or a few economically "optimal" scenarios, suggesting that only these system design options exist. We show a diversity of untold options to meet all energy demand based on renewable energy, with a complete phase-out of oil and gas imports. With a marginal increase above optimal cost, the reliance of an energy self-sufficient Europe on specific solutions, like biofuels, battery storage, transmission expansion, or heat electrification, can vary from not being used at all to being key to system stability.

With our work, policy makers can explore this option space. For instance, one can investigate where to locate hydrogen production hubs or look at the necessary trade-offs of imposing preferences like having to support consumers in electrifying heat and transport if biofuel use is minimized. Our code and data are open; hence, our approach can be applied to other continents or scaled to support decisions in specific regions.

INTRODUCTION

There is disagreement between models about what technical solutions are viable to achieve a carbon-neutral European energy system. Many studies have focused on a highly renewable electricity supply,[1–5] which has emerged as a credible way to achieve carbon-neutral energy, given the dramatic cost reductions of wind and photovoltaic power generation over the past decade.[6–8] However, techno-economic models designed primarily to understand the system integration of variable renewable generation use a high spatiotemporal resolution at the expense of considering the energy system beyond only electricity[1,9,10] or by making simplifying assumptions on full electrification of some sectors, leaving aside the role of non-electric carbon-neutral solutions.[2,11,12] Since there are in fact many non-electric energy end-uses,[2,13–15] these models often underestimate the scale of the transition and the extent to which Europe may need to remain dependent on fuel imports to meet energy demands.

In contrast, integrated assessment and energy-environment-economy models designed to understand climate mitigation pathways consider many interactions between human and earth systems, which includes all energy demands globally. However, they do this at the expense of spatiotemporal detail. Therefore, they underestimate the potential for sector coupling to help balance renewable variability.[16] The result is that pathway end-states from these models have generally contradicted the system designs from the former group of models, by suggesting

This is an open access article under the CC BY license (http://creativecommons.org/licenses/by/4.0/).

a significant need for firm capacity, including fossil-fired generation with carbon capture and storage (CCS).[8,17–19]

There is an additional drawback to most existing modeling studies: they generally consider a single, cost-optimal solution, or a limited set of cost-optimal scenarios. The growing field of modeling to generate alternatives (MGA) has shown how the realistic decision space is much broader.[20–22] However, no study has yet applied this approach to a Europe-wide high-resolution model including all energy demands.

Here, we address this gap to answer the question—what is the possible technological and spatial diversity in a self-sufficient and carbon-neutral European energy system based largely on renewable electricity generation? In doing so, we develop a model to represent all energy-consuming sectors in Europe with high resolution. We include demand in residential and commercial buildings; industry processes and feedstocks; passenger and freight transport by road, rail, air, and sea; and public services, agriculture, fisheries, and military facilities. We use a collection of novel methods to model demand and supply options across Europe at a high spatial and temporal resolution, tracking flows for electricity, heat, mobility, hydrogen, synthetic hydrocarbons, residual biofuels, and municipal waste. We summarize the main innovations and features of our model below and document them in detail in Note S1.

We use our model to explore the near-optimal decision space of an energy self-sufficient, carbon-neutral Europe and quantify trade-offs between competing interests according to nine high-level system metrics. We finish by discussing the implications of our exposed option space on the decision-making process. Given the extent of the option space, we cannot examine all trade-offs here. However, we release all of our results freely on Zenodo: https://doi.org/10.5281/zenodo.6546817, and our interactive web application at https://explore.callio.pe allows researchers and decision-makers to explore the impact of their preferences on the features of a self-sufficient, carbon-neutral European energy system.

SECTOR-COUPLED ENERGY SERVICE DEMAND IS UP TO 2.85× HIGHER THAN ELECTRICITY-ONLY DEMAND

We start by analyzing energy demand when grouping all energy consumption into four services (Figure 1). For each, we model total demand and its spatiotemporal variability using a combination of statistical datasets and simulation results (see experimental procedures and Note S1). The four main energy services are (1) space, water, and cooking heat demands (''building heat''); (2) hydrocarbon demands in place of fossil fuels for non-electrifiable industry processes or feedstocks as well as aviation and shipping (''synthetic fuel''); (3) the distance traveled by passenger, commercial, and freight vehicles on roads (''transport vehicle mileage''); and (4) electricity consumption by building-level appliances and cooling, passenger and freight rail, and industry processes (''electricity''). Most of these demands are based on 2018 levels (building heat, appliances, and cooling; transport distance; aviation and shipping fuels), whereas some are based on today's demands following electrification using today's technology efficiencies (rail and, where possible, industry processes) or a complete overhaul of processes to avoid reliance on fossil feedstocks (steel and chemical industries). Together, service demand in our model is 2.61–2.85 times (depending on the road transport technology choice) higher than electricity demand in 2018, highlighting the importance of our sector-coupled approach.

[1]Institute for Environmental Decisions, Department for Environmental Systems Science, ETH Zürich, Zürich, Switzerland

[2]Faculty of Technology, Policy and Management (TPM), Delft University of Technology, Delft, the Netherlands

[3]Lead contact

*Correspondence: bryn.pickering@usys.ethz.ch

https://doi.org/10.1016/j.joule.2022.05.009

CellPress
OPEN ACCESS

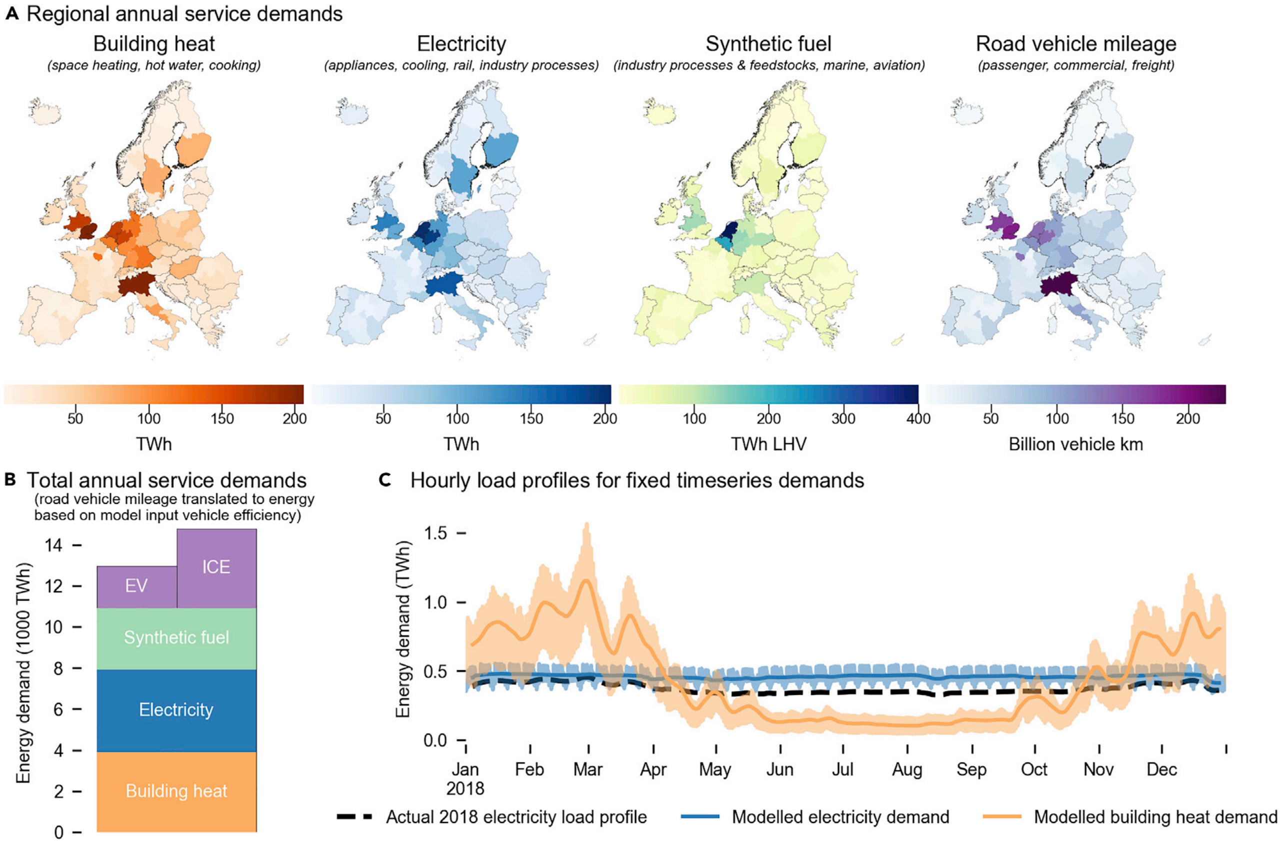

Figure 1. Modeled European energy demands and their spatiotemporal distribution

Magnitudes and spatiotemporal distributions of end-use service demands for the year 2018, resulting from the data processing pipeline described in the experimental procedures and Note S1. Service demands for the year 2018 are used as an input to the baseline model runs to match the 2018 weather year used to define renewable technology capacity factor profiles. We also model demands for the years 2010–2017 with their respective weather data, which we use for sensitivity analyses (see Note S2).

(A) Annual demand per sectoral group and modeled European region in 2018. ''Building heat'' refers to space heat, hot water, and cooking demand in residential, commercial, and industrial buildings. ''Electricity'' refers to all direct electrical end-use demand, based on historical electricity consumption minus electricity consumed to meet building heat and road transport demand plus additional demand from electrifying all rail and electrifying most industrial processes. ''Synthetic fuel'' refers to demand from industry for liquid and gaseous hydrocarbons as feedstock (e.g., methanol for chemicals) and for high temperature process heat (where it cannot be electrified), and for liquid fuel demands in domestic and international aviation and shipping. Fixed demand for hydrogen and CO_2 in industry is assumed to be directly electrified and thus combined into ''electricity'' demand. Road vehicle mileage encompasses all road vehicles; rail has been assumed to be fully electrified and is combined into ''electricity.''

(B) Total annual energy demand for the whole system in 2018. Groupings are the same as given in (A), with transport demand converted from vehicle km to demand for energy based on the entire fleet being electrified (EV) or the entire fleet consuming liquid fuels in internal combustion engines (ICE).

(C) Hourly (transparent area) and seven-day rolling average (solid line) demand of end-use electricity and building heat demand. Groupings are the same as given in (A). ''Current electricity load'' refers to 2018 electricity load across all modeled countries, according to ENTSO-E published statistics. For visual clarity, only the seven-day rolling average data are given for actual 2018 electricity load.

We explore how these demands can be met by a predominantly renewable energy system relying on proven and commercially available technologies as far as possible, in agreement with previous work[23] that has shown how waiting for unproven technologies to become available could result in substantially higher transition costs. This means we assume that building heat and transport vehicle demands can be met directly by electricity, with biofuel- or electricity-derived hydrocarbons, or with direct use of biofuels or municipal waste (heat only). This illustrates two potential distributions of burden across society: (1) industry and utility-scale actors drive a new synthetic fuel generation industry, enabling consumers to continue meeting demand with hydrocarbon-reliant end-use technologies, and (2) consumers electrify

their end-use technologies alongside the transformation of local supporting infrastructure, such as electric vehicle charging and reinforced local electricity distribution networks. To enable the transmission of energy between regions, we assume that high-voltage electricity grids and fuel transport infrastructure are available. Hydrogen can be used as a feedstock in producing synthetic fuels or for utility-scale stationary storage. However, we do not consider its direct use for road transport or building heat due to the need for an overhaul of transmission networks as well as end-use technologies to enable distributed hydrogen use[24] and the emerging market dominance of electrification, for instance, in passenger and freight vehicles.[25,26] Because of the limited availability of non-electrically derived carriers to satisfy most energy demands, namely biofuels and municipal waste, the total primary supply of electricity increases.

Today, demand is distributed unevenly across Europe (Figure 1A). For example, because of its large petrochemical industry, the Netherlands stands out as a large fuel consumer. The high population density of south-eastern United Kingdom, northern Italy, and north-western Germany concentrate electricity, heat, and transport demand. Building heat demand is not only spatially diverse, but its temperature-dependence leads to pronounced seasonal variability (Figure 1C). Because both demand and renewable energy supply vary in space and time, modeling the design of a continent-spanning renewable energy system for all energy-consuming sectors requires representing this variability with sufficient detail. Therefore, we build a linear optimization model with 98 nodes and a 2 h temporal resolution for 4,380 time steps over a full calendar year, with the objective to supply energy at lowest total cost. We enforce constraints to ensure that all demands are met while restrictions on the deployment of generation and transmission technologies are respected. We then systematically explore options close to the least-cost optimum, generating 441 technically and economically feasible, spatially-explicit practically optimal results (SPORES[21]). These SPORES represent feasible system designs in which all European demands for energy-consuming services can be met, based on 2018 magnitudes and spatiotemporal distributions, without any energy imports (for analyses concerning different weather years and a projected annual demand scenario, see Note S2). There are an infinite number of alternative configurations; our method, which is an extension of conventional MGA,[27] specifically looks for those which expose the greatest diversity in technology choice and spatial configuration within 10% of the least-cost system.

DIVERSE RANGE OF FEASIBLE DESIGNS FOR A SELF-SUFFICIENT, CARBON-NEUTRAL EUROPEAN ENERGY SYSTEM

We find that many near-optimal energy system configurations based predominantly on solar and wind electricity can supply all European energy demand. Across all SPORES, electrification efficiency gains would decrease primary energy supply compared with today, as illustrated by the two options that meet demand with the highest and lowest primary energy supply in Figure 2: both are lower than primary energy supply today.

Since we do not allow energy imports into our model region, the supply in all SPORES is generated within Europe. This shows that it is feasible to eliminate net imports currently equivalent to almost half of primary energy supply (9,122 TWh in 2018, predominantly by import of fossil fuels). Fixed synthetic fuel demands for industry processes, and marine and aviation fuels, mean that primary energy supply does not reduce as much as might be expected between historical levels and those given by our SPORES. That is, efficiency gains from electrification are partly offset by inefficient processes to produce carbon-neutral fuels in all SPORES. Exactly how much these inefficient intermediate processes affect the total primary energy

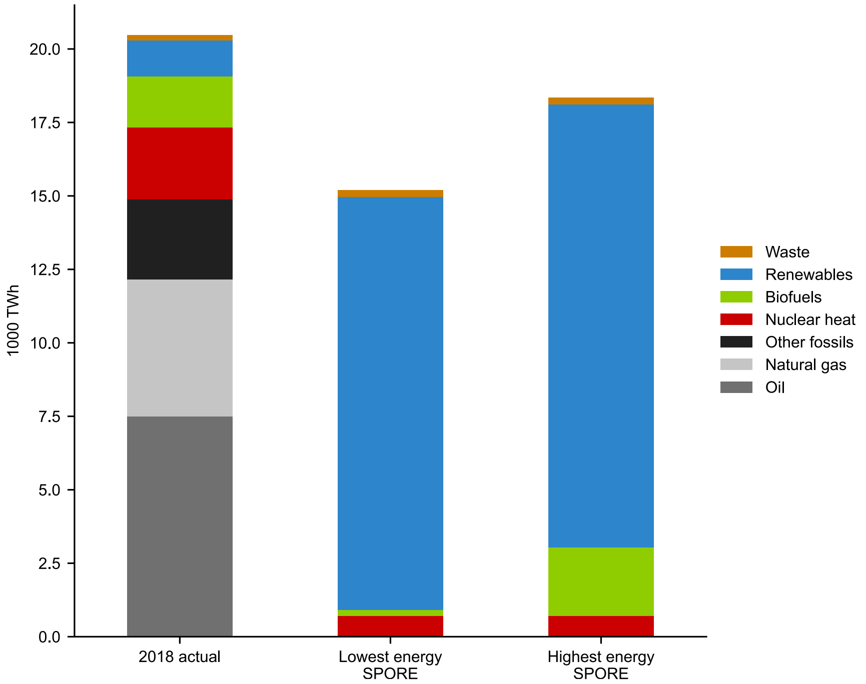

Figure 2. Current European gross available energy (GAE) compared with highest and lowest GAE in modeled energy self-sufficient, carbon-neutral Europe

Gross available energy for 34 European countries. "2018 actual" refers to 2018 data from the Eurostat annual energy balances, category *GAE*, that includes primary production, recycled and recovered products, changes in stock, and net imports. For more detail on mapping of Eurostat energy carriers to technology groupings in this figure; see primary energy supply. "Lowest energy SPORE" refers to a feasible energy system within 10% of the cost-optimal solution with the lowest overall primary energy supply. "Highest energy SPORE" refers to a feasible energy system within 10% of the cost-optimal solution with the highest overall primary energy supply. Since the end-use service demands are fixed across SPORES, the differences in primary energy supply are caused by inefficiencies in intermediate processes, such as producing synthetic fuels from electricity-derived hydrogen. Primary energy supply in the model result includes electricity directly produced by renewables (wind, solar, and hydropower), municipal waste, residual biofuel, and nuclear heat. Modeled nuclear heat is calculated according to the Eurostat energy balances as nuclear power electricity production divided by average plant efficiency. All countries in the study area except Switzerland are included.

depends on the approach to carbon neutrality taken in each sector, since the options to meet energy service demands have different efficiencies. For example, less energy is required for the same total distance traveled by vehicles if these vehicles are fully electric than if they still use liquid fuels (Figure 1B). The more Europe relies on its current means of heating (especially methane) and road transport (oil), the more primary electricity supply is required to manufacture carbon-neutral fuels.

The extent to which sectors should be electrified is just one of many decisions that can be taken when designing a carbon-neutral, renewable European energy system. Our 441 different SPORES allow us to explore how extensive this decision space is and to quantify the trade-offs between preferences in detail.

MANEUVERING SPACE EXISTS BETWEEN COMPETING INTERESTS WITHOUT COMPROMISING COST-EFFECTIVENESS

We first choose a set of metrics that quantify aspects that have received particular attention in energy policy debates, such as the uncertain role of energy storage in

Table 1. Definition of high-level energy system metrics and their range across the decision space

Metric name	Metric description	Metric range
Storage discharge capacity	total capacity of all storage technologies to discharge energy in any given hour, including low-temperature heat, hydrogen, and electricity	0.03–11 TW
Curtailment	percentage of maximum available renewable electricity production from wind and solar photovoltaic technologies that is curtailed	0%–6%
Biofuel utilization	percentage of available residual biofuels that are consumed	0%–100%
Average national import	average annual import of electricity across all countries within the study area	4–69 TWh
Electricity production Gini coefficient	degree of inequality of spatial distribution of electricity across all model regions, measured by the Gini coefficient of regional electricity production	0.54–0.74
Fuel autarky Gini coefficient	degree of inequality of spatial distribution of industry synthetic fuel production relative to industry fuel demand across all model regions, measured by the Gini coefficient of regional overproduction	0.64–0.99
EV as flexibility	Pearson correlation between timeseries of electric vehicle charging and that of primary electricity supply	0.52–0.92
Heat electrification	percentage of heat demand met by electricity-consuming, heat-producing technologies	4%–100%
Transport electrification	percentage of road passenger and freight transport demand met by electric vehicles	53%–100%

Definition of high-level metrics that describe energy systems that may be particularly relevant to specific stakeholders or interest groups, and the range of values of each metric across all SPORES results. The metric values across all SPORES are shown scaled relative to their maximum values in Figures 3 and 4.

highly renewable energy systems[28–31] or the extent to which countries are electricity autarkic[32] (Table 1). We formulate the metrics such that lower values are more preferable in a broad sense: more equally distributed infrastructure, less electrification (i.e., less consumer-level change), or less use of possibly problematic or controversial technologies like energy storage or biofuels. Figure 3A illustrates the range of each metric across all SPORES after scaling the metric relative to its highest value in any SPORE. Sub-selections of SPORES within 15 percentage points of the lowest value for each scaled metric are highlighted in colored boxes (the "+15pp range"). We see that for some metrics, such as biofuel utilization, there are solutions across the entire range. This means that there are near-optimal solutions that use all of the available biofuel potential and others that use next to none of it. For other metrics, most of the possible energy system configurations are within a narrow band; e.g., road transport does not go below 53% electrified in any SPORE.

We can investigate how severely the decision space is constrained if we are concerned about one particular metric, for example, the use of energy storage (utility-scale batteries, hydrogen tank storage, and low-temperature heat storage). In Figure 3B, we plot the range that each of the remaining metrics can take when one metric is held to within its +15pp range. We see that even when constraining storage discharge capacity, there is still maneuvering space to choose the degree to which we wish our energy system to depend on biofuels, expansion of the electricity transmission network, or the electrification of building heat. Similarly, electricity and synthetic fuel production can be distributed more evenly across Europe (lower gini coefficients) while retaining maneuvering space in most other metrics.

CellPress
OPEN ACCESS

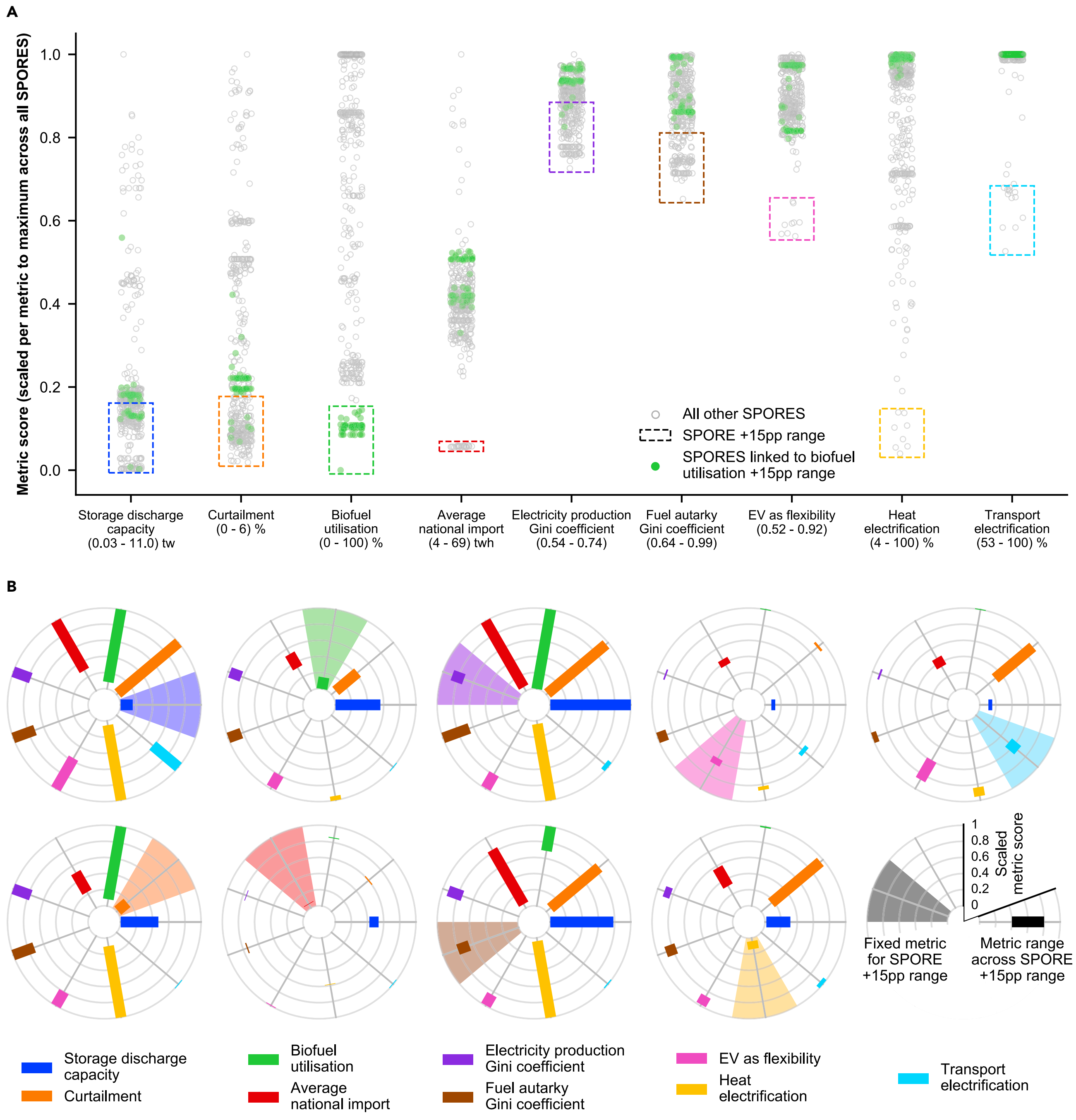

Figure 3. Near-optimal decision space described by 441 energy system configurations

Decision space of 441 feasible energy system configurations (SPORES) within 10% of the cost-optimal total system cost, as shown through a selection of nine metrics that may be particularly relevant for decision-makers making energy system planning decisions.

(A) Performance of SPORES, according to scores for the nine different metrics scaled to their maximum value in any SPORE. SPORE ranges do not describe a statistical distribution since our method is focused on exploring options around the periphery of the decision space (for more detail, see SPORES). The SPORES within 15 percentage points of the lowest score per metric after scaling (the "+15pp range") are highlighted by colored boxes. All SPORES connected with the +15pp range of biofuel utilization are colored green, which we use to define the metric ranges depicted in (B). See Note S4 for SPORES highlighted according to the +15pp range of other metrics. Horizontal placement of SPORE markers in each metric is random to better view the spread of data points that would otherwise overlap, using the "jitter" functionality provided by the Python package *Seaborn*.

Figure 3. ***Continued***

(B) Scaled range that the other eight metrics can take (shown as bars) when one metric is fixed to within the +15pp range highlighted in (A). Each fixed metric is shown as a bar with a highlighted background. Each radial chart holds one metric to within the +15pp range and therefore highlights how strongly the decision space is constrained if a low value for that metric is of particular importance. Colors of metric bars and background highlights in (B) match the colors of the boxes surrounding the +15pp range of SPORES in (A).

However, this distribution does require vehicle electrification rates to be close to 100%.

Constraining biofuel utilization to its +15pp range dramatically reduces the decision space remaining on most other metrics. Given the technological options we consider, it implies a high degree of heat electrification and of using electric vehicles as a flexibility source in the power system. The maneuvering space is even more restricted when electricity transmission network utilization is kept low, implying effectively fixed metric values in all other SPORES.

Even with more constrained sets of solutions, maneuvering space still exists. However, the more we wish to maintain specific preferences, the more the remaining maneuvering space is reduced. It is not possible to keep all metrics within their lower bound: at most, four metrics can be within +15pp of their lowest scaled values before trade-offs have to be made.

Furthermore, there are many relevant dimensions beyond the nine metrics we start our analysis with. For example, we find that many resulting configurations place a large amount of synthetic fuel production in Britain and Ireland, exploiting the particularly high wind power potential in that area. We also find that many system configurations rely on wind more than on PV.

To explore these dimensions and the effects of trade-offs across multiple high-level metrics, we select four example SPORES for more in-depth analysis. We choose two SPORES on trade-offs from the nine metrics: both keep storage use, curtailment, and biofuel use low, with one prioritizing the minimization of curtailment and the other of biofuel use. The other two SPORES we choose are aimed at exploring the aforementioned dimensions of the system beyond our chosen metrics: both keep storage use low, with one having PV capacity in the 90th percentile of capacities across SPORES and the other having fuel production within Britain and Ireland below 10%. We highlight the four resulting SPORES in Figure 4. Since there are an almost infinite number of reasons to select SPORES for more detailed analysis and comparison, we use our four selected SPORES as an example of how to hone in on features of interest in the option space. Other researchers and decision-makers will want to focus on other aspects from the many dimensions which our set of results spans, which they can do using our interactive data explorer: https://explore.callio.pe.

We see that in some instances, two different rationales for imposing preferences can lead to similar impacts on metrics that have not been considered. High PV capacity and moving fuel production outside Britain and Ireland lead to the selection of SPORES with a similar degree of technology curtailment and the same biofuel utilization. However, that is where the similarities end; there is markedly different dependence on transmission and heat electrification of these two configurations. Infrastructure planning is a key aspect of the energy transition and one key aspect of our method is to explore the spatial diversity of infrastructure deployment. Indeed, a criterion like "low use of storage" can have a wide variety of regional effects. To explore this, we turn to examining the spatial dimension of our four selected example SPORES.

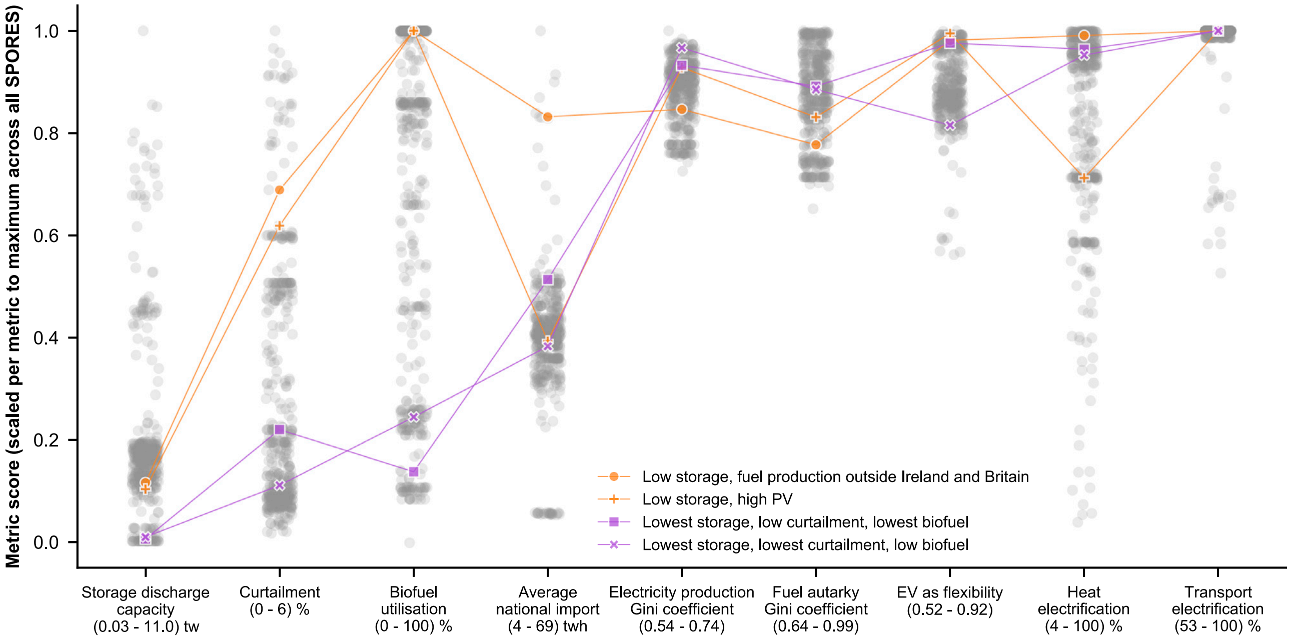

Figure 4. Position of four example energy system configurations in the near-optimal decision space

Four example SPORES selected from the total number of 441 to cover both low and medium deployment of storage discharge capacity and different allowed degrees of curtailment, biofuel consumption, and spatial distribution of generation and synthetic fuel production. Connecting lines between markers do not imply data interpolation between metrics but are to visually aid the trade-offs between metrics for a specific SPORE. These examples illustrate the synergies and trade-offs that open up between competing goals across all predefined metrics (see Table 1) but should not be considered the only trade-offs that can be analyzed from our results. Readers are encouraged to use these examples as a guide to explore further trade-offs themselves, with our interactive data explorer: https://explore.callio.pe. Highlighted in purple are the two SPORES selected based on keeping storage discharge capacity below 0.1 of its scaled score (''lowest storage'') and one of curtailment or biofuel utilization below 0.3 (''low''), while the other is minimized (''lowest''). Highlighted in orange are the two SPORES selected based on keeping storage discharge capacity below 0.2 (''medium storage'') while defining preferences outside the scope of the metrics. One is chosen to maximize total PV deployment in Europe (''high PV''); the other is chosen by filtering SPORES to those in which hydrogen production in Britain and Ireland is below 10% of total European hydrogen production (''fuel production outside Ireland and Britain''). Overlaps between SPORES on any metric are purely coincidental, showing how trade-offs are not immediately obvious from the primary selection criteria of a SPORE. Metric values across all SPORES (gray circles) do not describe a statistical distribution, since our method is focused on exploring options around the periphery of the decision space (for more detail, see SPORES). Horizontal placement of SPORE markers in each metric is random to better view the spread of data points that would otherwise overlap, using the ''jitter'' functionality provided by the Python package *Seaborn*.

DIFFERENT SPATIAL CONFIGURATIONS CAN SATISFY HIGH-LEVEL CONSTRAINTS EQUALLY WELL

We now compare our four selected example SPORES with respect to wind farm and PV deployment, net electricity imports and contributions to carbon-neutral fuel production, and expansion of the electricity transmission grid. Maintaining low reliance on storage, biofuels, and curtailment tends to require capitalizing on the high wind power productivity around Britain and Ireland to create hydrogen production hubs. However, capacity deployment can still vary greatly (Figures 5A and 5D). For instance, wind capacity can be split differently between Ireland and Great Britain and between onshore and offshore wind farms. In addition, large hydrogen production facilities could either be entirely concentrated in Britain and Ireland (Figure 5E) or include hotspots in Spain, France, and the Netherlands (Figure 5B). Strong reinforcement of transmission lines between northern European regions would occur in both cases (Figures 5C and 5F), but further reinforcements toward the Iberian peninsula would be required in the case in which generation capacity is more distributed (Figure 5C).

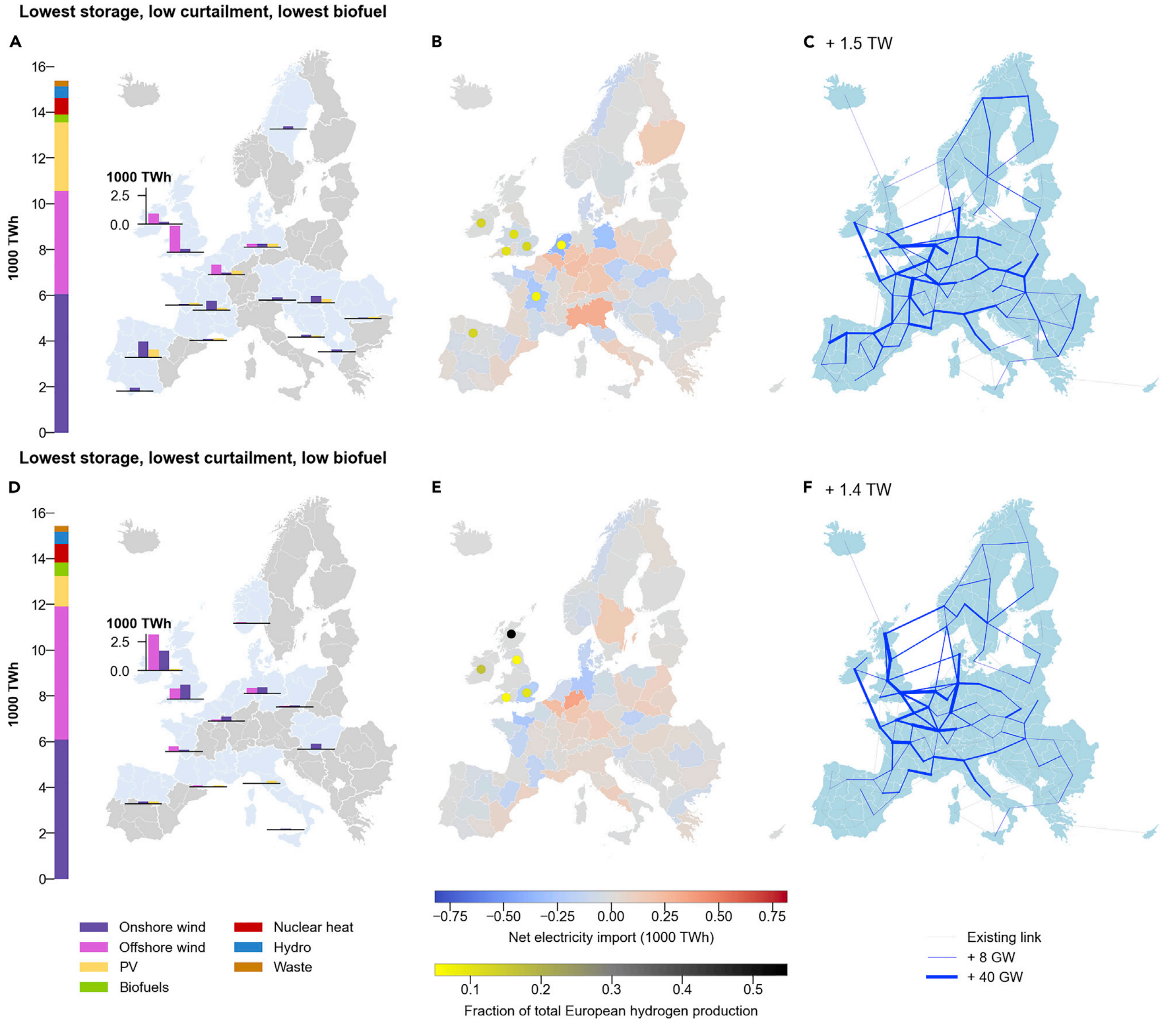

Figure 5. Spatial distribution of energy generation and total primary energy supply of two example energy system configurations in the near-optimal decision space

Spatial distributions and total primary energy supply for two of the four selected SPORES defined in Figure 4; "lowest storage, low curtailment lowest biofuel" (top) and "lowest storage, lowest curtailment, low biofuel" (bottom). Readers are encouraged to use these examples as a guide to explore further trade-offs themselves, with our interactive data explorer: https://explore.callio.pe.

(A and D) Spatial distribution of onshore wind, offshore wind, and PV supply (right) and total primary energy supply across all regions (left). Capacities are shown for 29 zones that are aggregated from the 98 model regions to give comparable land area. Zonal supply is only shown when the sum of supply in that zone is greater than 6% of maximum supply from one technology in any region, making it visually easier to see the major supply hubs. All zones for which supply is not shown constitute 8%–11% of total European supply. Cyprus is not shown, but these data are included in the same zone as Greece in the maps. Biofuels, waste, hydro, and nuclear electricity supply are not shown on the maps.

(B and E) Annual regional net electricity import and high synthetic fuel-producing regions. Data are shown at the resolution of the 98 model regions. For each region, annual net electricity import is the sum of all electricity imported from connected regions over the year minus electricity exported to connected regions over the year. A positive net import indicates a region imports more electricity than it exports, while a negative net imports indicates more exports than imports. High synthetic fuel-producing regions are those producing above 5% of European total hydrogen. Since hydrogen cannot be transported between regions in our models or directly consumed to meet service demands, high hydrogen production is equivalent to high synthetic fuel production.

(C and F) Electricity grid transmission expansion beyond existing or planned capacities between regions. In the top-left of each panel is the total line capacity added across Europe above the baseline capacities for that feasible configuration. Light gray lines depict regions connected by transmission lines that are not expanded. Where transmission expansion occurs, lines are shown in blue, with increasing thickness indicating increasing transmission expansion. Two values in the legend mapping expansion to line thickness refer to the mean and maximum expansion of any one line of all those that are expanded.

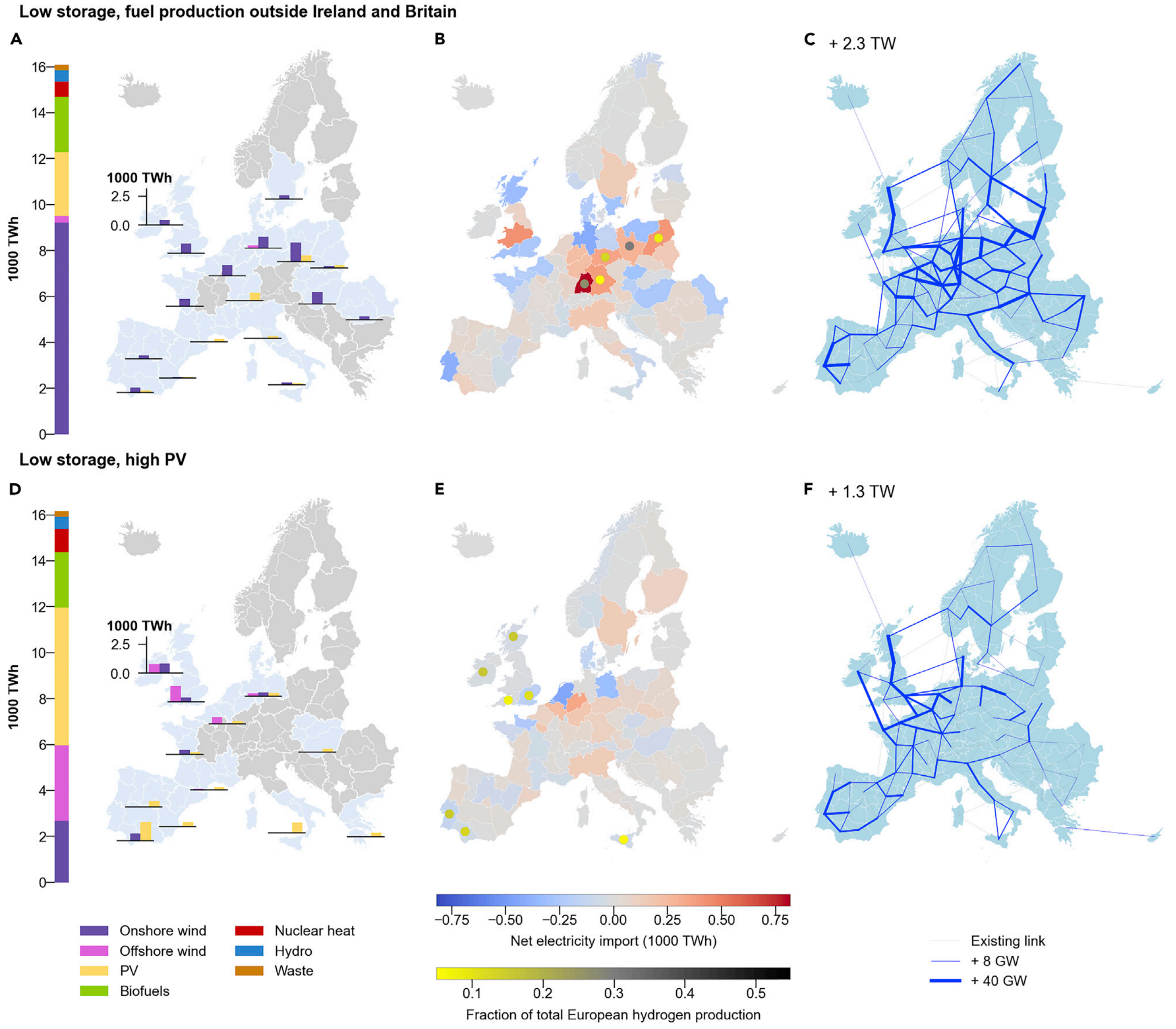

Figure 6. Spatial distribution of energy generation and total primary energy supply of two example energy system configurations in the near-optimal decision space

Spatial distributions and total primary energy supply for two of the four selected SPORES defined in Figure 4; "low storage, fuel production outside Ireland and Britain" (top) and "low storage, high PV" (bottom). For a full description of each panel, refer to the caption of Figure 5, which shows the same information for two other SPORES

When we slightly relax our restrictions on high-level metrics, we can see that radically different system configurations are possible. For instance, it is possible to move fuel production outside Britain and Ireland to eastern European countries (Figure 6A). In this deployment strategy, such countries would become net electricity importers and key hubs for the production of hydrogen (Figure 6B). This would be made possible by marked expansion of transmission lines throughout the continent (Figure 6C) and by full utilization of residual biofuels. Alternatively, similar relaxations on storage deployment and biofuel use could allow a much larger deployment of solar capacity in southern Europe, combined with a substantially lower deployment of wind overall (Figure 6D). This would enable splitting hydrogen production facilities into southern and northern hubs (Figure 6E) and would come with lower

requirements in terms of infrastructural change, such as a moderate electrification of the heat sector and a limited expansion of transmission lines (Figure 6F).

These are just two examples of how a high-level goal can be met by very different system configurations. In both cases, many more options can be drawn from our full set of results. We can thus conclude that there is considerable maneuvering space for infrastructure siting, even when we want certain high-level preferences to be fulfilled and that this maneuvering space expands even more when we are willing to compromise on some metrics, such as allowing more biofuel use.

DISCUSSION

Our analysis leads to three important implications for the implementation of a self-sufficient, carbon-neutral European energy system. These results are relevant both to guide the development of detailed pathways in future academic work and for policy makers to decide on priorities when translating decarbonization targets into more concrete plans. First, we find that there is a diversity of options to design a self-sufficient, carbon-neutral energy system relying as much as possible on technologies that are already available. This requires a large expansion of wind and solar power together with electrification and synthetic fuels. This finding is strengthened by our high spatiotemporal detail on both energy demand and renewable supply, which enables our model to depict both the drawbacks of renewable variability and the benefits of sector coupling to deal with this variability.

Second, we find that there are few must haves: a system can be designed to rely to a varying extent on wind energy, PV, biofuels, intra-European transmission, storage, electrification of heat and transport, or use of controlled vehicle charging. We also find that "firm generation" is not strictly necessary. This result differs from many recent studies that expect a reasonable proportion of demand to be met by some form of firm capacity, be it through biofuels,[11] nuclear,[33] or fossil generation with measures to offset or capture emissions.[19,34,35] Our portfolio of configurations also encompasses recent studies that do not use firm capacity. Bogdanov et al.[5] present wind (32%) and PV (62%) as the two primary electricity sources in a carbon-neutral Europe. Pleßmann and Blechinger[36] agree on the extent of renewables, but with an opposite ratio of 63% from wind and 20% from PV; in other studies, wind and PV ratios sit somewhere in between.[12,37,38] We show that all of these studies are correct, in that all of these solutions are possible. When considering the impact of PV-to-wind ratio on seasonal balancing of variability, we find that there is substantial flexibility on what solutions to deploy to address this problem, although generally in high solar production years, summer overproduction is dealt with by more electrolysis and winter underproduction dealt with by combined-cycle gas turbines (CCGTs) and hydropower (see Note S3). By modeling an entire decision space rather than a single solution, we can quantify the trade-offs between choices, supporting decision-makers in their deliberations.

Third, there is a great deal of flexibility in where to locate infrastructure in Europe. We show that specific regions, like Britain and Ireland or eastern Europe, can be prioritized for electricity or fuel production. As regional equity becomes increasingly important in the transition discourse, an understanding of this regional maneuvering space can support discussions on benefits and drawbacks of new infrastructure. For example, regions could capitalize on becoming synthetic fuel production hubs. Our modeling approach allows decision-makers to examine different spatial configurations, which can inform incentive schemes to foster such regional economic

development. In addition, we show it is possible to eliminate the need for fossil fuel imports from outside of Europe. The resulting economic benefits captured within Europe itself could be considerable, while simultaneously ensuring security of supply.

Our sensitivity analyses confirm the robustness of these conclusions, with respect to the allowable cost relaxation and the choice of weather year (and thus variability of renewable generation and demand). In addition, although we do not project future energy service demands for our main results, given the uncertainty associated with them, our sensitivity analysis shows that our decision space remains qualitatively the same if we use simulated changes in annual demand to 2050[39,40] (see Note S2). Nevertheless, there are several limitations in our work. The true robustness of system designs to a range of weather conditions expected to occur over the decades of investment lifetime needs more work bringing together climate science, meteorology, and energy engineering.[41] Furthermore, in our model, the technological and spatial maneuvering space is enabled by a willingness to have a system up to 10% more expensive than the cost-minimal continent-wide system configuration. The option space diminishes if we restrict this willingness to pay and increases with a greater willingness (see experimental procedures and Figure S17). However, for a model of all energy-using sectors, "total system cost" is difficult to interpret, given that there is no single actor to which these costs accrue; therefore, we refrain from making statements about the cost of the transformation. Still, it is possible to examine the designs from the perspective of the burden carried by different groups of actors in different regions—for example, consumers (rooftop PV, car purchases, and heating technology choice), utilities (power plants or grid expansion), and industries (process changes and synthetic fuel generation). This offers a basis for future work to focus on.

It is also important not to interpret our results as a forecast or prediction of the future. Instead, they are a systematic exploration of a fully linearized representation of the European energy system design space under the broad assumptions we outline above and in Note S1, like the large-scale reliance on variable renewable generation. Further work is required to broaden the scope of available technological solutions and to analyze the impact of nonlinear processes, such as transmission grid power flow. However, a model like ours can only be run on specialized high-performance computers due to its complexity. Therefore, a practical approach to conducting further work is to first constrain the option space based on features of interest and then generate near-optimal solutions with updated assumptions in this constrained space. That is, we recommend expending effort only on the parts of the design space that are of interest to decision-makers. This approach would also be necessary to examine carbon-neutral system designs from perspectives that we do not consider in this study, such as macro-economic impacts, local infrastructure effects, and feasible transition pathways. Indeed, investigating possible lock-in effects, including how the option space becomes increasingly constrained the longer we wait to make decisions, is an urgent problem that requires more attention in future work.

We show a broad variety of ways to build a carbon-neutral energy system that meets all European energy demands. However, to avoid catastrophic climate change, the scale of the transformation and the speed at which it must proceed remain enormous. For instance, if we assume that the deployment of renewables in European follows an "S-curve" to 2050,[42] then the maximum growth requirements of combined wind and PV in Europe would be 681–932 TWh/year across our option space. This requires a ten-fold increase in annual growth by the early 2030s, when compared

with Europe's maximum historical annual growth of 66 TWh/year. For this reason, we focus on technologies that are ready to scale up or are already in the process of scaling up rapidly, with two exceptions: some industry processes in the steel and chemicals subsectors and the formation of a synthetic fuel industry that can manufacture liquid and gaseous fuels from electricity and biofuels. Irrespective of the exact system configuration, large-scale infrastructure deployment is necessary. The solutions we show will require deployment of renewable energy at an unprecedented scale and will affect all industry subsectors and all individuals in their homes, vehicles, and workplaces.

The maneuvering space we identify decreases substantially as soon as we wish certain preferences to be fulfilled: for example, if we wish to completely forego the use of biofuels or energy storage. Understanding such trade-offs—and the implications they have for how quickly the continent can reach carbon neutrality—is important for decision-makers and for society as a whole. This requires bringing stakeholder views into the techno-economic modeling process and reflecting the real decision space back to stakeholders.[43] Ultimately, the extent to which our model is useful depends on whether it captures real-world trade-offs; as academics, we are not in the best position to make this judgment. For this reason, we make all results available to actual decision-makers through our web interface (https://explore.callio.pe) and encourage further work to explicitly bring real-world decision-makers into the loop of modeling exercises. By using an approach like ours to guide and structure the process on narrowing down the economically, socially, and politically acceptable design space for the target system, follow-up analyses can investigate specific technical aspects—such as grid operation—in more detail and can examine possible pathways and supporting policy mechanisms to reach the target design. Our analysis of the trade-offs within the designs we select above can be seen as a guide for others to explore the myriad additional trade-offs further, using our models, data, and interactive interface.

EXPERIMENTAL PROCEDURES

Resource availability

Lead contact

Further information and requests for resources and materials should be directed to and will be fulfilled by the lead contact, Bryn Pickering (bryn.pickering@usys.ethz.ch).

Materials availability

The model data processing workflow generated in this study has been deposited to GitHub: https://github.com/calliope-project/sector-coupled-euro-calliope. The final model using the baseline 2018 data, ready for use in Calliope, has been deposited to Zenodo: https://doi.org/10.5281/zenodo.5774988. All model results, for all baseline SPORES and sensitivity runs, have been deposited to Zenodo: https://doi.org/10.5281/zenodo.6546817.

Data and code availability

All code and data associated with this study are available on GitHub: https://github.com/calliope-project/sector-coupled-euro-calliope and Zenodo: https://doi.org/10.5281/zenodo.6546817.

European energy system model setup

The European energy system model is an expansion of the stylized power system model Euro-Calliope v1.0.[1] Our sector-coupled Euro-Calliope model takes the current configuration of *all* European energy consumption as a departure point to

CellPress
OPEN ACCESS

model credible future configurations in a realistic manner. Compared to the single energy carrier considered in power system models, we represent 13 carriers in our sector-coupled Euro-Calliope: electricity, hydrogen, CO_2, liquid and gaseous hydrocarbons (kerosene, methanol, diesel, and methane), solids (residual biofuel and municipal waste), low-temperature heat (combined space heat and hot water, and cooking heat), and vehicle distance (heavy- and light-duty road vehicles). These carriers can be consumed, produced, and converted by a variety of technologies to meet demand. In addition, low-temperature heat, hydrogen, electricity, and methane can be stored. Since future international energy commodity prices are highly uncertain, energy imports from outside our model region are not allowed. Accordingly, all our model results represent system designs in an energy self-sufficient Europe. We describe the key components of input data processing in the following subsections, with an overview of primary data sources given in Table 2. Further details on the model setup and data processing are provided in Note S1, and the full representation of carrier and technology connections is given in Figure S13.

The model is optimized as a linear programming problem at two-hour resolution over a whole year using the Calliope energy system modeling framework.[44] The base year of the study, for weather and demand data, is 2018. We choose this year as we have the most complete statistical datasets available, thus requiring the least amount of gap-filling. For instance, prior to 2016/7, Albania and Bosnia and Herzegovina have limited data availability from Eurostat and ENTSO-E. See sensitivity analyses for information on the additional years used as sensitivity analyses. The full model workflow, including references to all data sources and the processing steps to generate the model, is freely and openly available online (see resource availability).

Demand data

We source annual demand data from the Eurostat,[45] JRC-IDEES,[46] and Open Power System Data[47] databases. We do not make any assumptions on changes in demand for services, such that our model is looking at a feasible, carbon-neutral configurations that would work with demand as we know it today. We do this for two reasons. First, the demand for services in the future is highly uncertain, with assumptions varying depending on modeling group and scenario.[19] To take a specific example of this problem, the EU reference scenario 2020 assumes a 26% increase in distance traveled,[48] while Bogdanov et al.[5] assume an 80% increase. Second, our focus is on the features of system design exhibited when modeling energy service demands resolved in space and time. Because of this high resolution, we are able to model the potential for flexibility and sectoral coupling in the design of the energy system. This could make things easier, for example by balancing variable renewable generation with flexible charging of electrified transport. It could also make things harder, for example by adding additional pressure on the transmission system because of electrification of processes. It is therefore important to have synchronized energy demand and weather profiles, both in time and space. Without synchronicity, we risk missing the effect of sub-daily to seasonal meteorological phenomena that influence variable renewable supply as well as demand, both for heat in buildings and for electricity.[49,50] Data from recent years are inherently synchronized; applying demand assumptions might lead us to unknowingly break this synchronicity. Although we do not attempt to project demand, the increase in final energy consumption given by our SPORE results (2018 to 2050: +(73%–93%) is in line with that given by the EU reference scenario 2020 (2015 to 2050: +84%), and remains in line when analyzing individual subsectors (industry, buildings, and land transport). Therefore, although we do not assume increases in service demands or decreases

Table 2. Summary of primary sources used in data processing pipeline

Model component	Temporal processing: Annual	Temporal processing: Hourly	Spatial processing: National	Spatial processing: Sub-national
Electrified rail	Eurostat & JRC-IDEES	DESSTINEE	Eurostat & JRC-IDEES	population & industry density
Road transport	Eurostat & JRC-IDEES	RAMP-mobility	Eurostat & JRC-IDEES	population & industry density
Aviation	Eurostat	–	Eurostat	industry density
Shipping	Eurostat	–	Eurostat	industry density
Existing industry processes	Eurostat & JRC-IDEES	–	Eurostat & JRC-IDEES	industry density
New industry processes	Eurostat, JRC-IDEES, & literature	–	Eurostat & JRC-IDEES	industry density
Buildings: cooking	Eurostat & JRC-IDEES	RAMP-cooking	Eurostat & JRC-IDEES	population
Buildings: heat and hot water	Eurostat & JRC-IDEES	MERRA-2 & When2Heat	Eurostat & JRC-IDEES	population
Buildings: appliances and cooling	–	OPSD	OPSD	population
Viable nuclear regions	–	–	–	JRC powerplant database
Biofuel supply capacity	JRC ENSPRESO	–	JRC ENSPRESO	land use categorization
Municipal waste supply capacity	Eurostat	–	Eurostat	JRC powerplant database
Heat pump performance	–	MERRA-2 & WAKAM technology database	–	population & MERRA-2
Gas cavern storage capacity	–	–	GIE	equal distribution
PV and wind capacity factors	–	renewables.ninja	–	renewables.ninja & land use
PV and wind capacity limits	–	–	–	land use categorization
Hydro capacity factors	IRENA	MERRA-2 & atlite	IRENA	JRC Hydro database v7
Hydro capacity	–	–	JRC Hydro database v7	JRC Hydro database v7

Summary of primary sources used to process spatiotemporal demand and supply data to use as inputs to the sector-coupled Euro-Calliope model

in demand intensity due to efficiency improvements, our resulting system representation is not inconsistent with those studies which do make such assumptions. Nevertheless, to assess whether changes in demand assumptions could make an impact on our modeling results, we have generated a subset of SPORES with scaled demands (see Note S2). These resulting SPORES suggest that the conclusions we draw from our study on the extent of the near-optimal option space is not affected by our use of 2018 service demands.

We group building heat demand into three end-uses: space heat, hot water, and cooking. These groups match the Eurostat database household end-use categorization, national data for which became available in 2020 (dataset: *nrg_d_hhq*). Commercial and Industrial sector building heat demands are not available on Eurostat, so we use JRC-IDEES, which has data for the period 2010–2015. We transform fuel consumption to a demand for heat by assuming technology efficiencies of heating technologies including boilers and direct electric heaters (see Table S2). These efficiencies are consistent with those used in our model for the available heat supply technologies. We use annual water and space heat demands to scale normalized hourly demand profiles produced using the methods implemented for the When2Heat database,[51] updated to account for (1) all countries in our model scope and (2) the sub-national distribution of single- to multi-family homes across Europe, according to the Eurostat database of dwellings (dataset: *cens_11dwob_r3*). To generate cooking heat demand profiles, we extend the open-source RAMP engine[52,53] to stochastically model demand in all European countries from the bottom up.

The transport sector encompasses road, rail, air, and shipping. We assume electrification is only possible in some of these forms of transport, namely road and rail. In

CellPress
OPEN ACCESS

rail, we assume complete electrification, taking current consumption of fuel for rail from the Eurostat annual energy balances (dataset: *nrg_bal_c*) and converting it to electricity demand using the efficiency of different rail drivetrains from JRC-IDEES. For airplanes and shipping, we take domestic and international fuel demands directly from the Eurostat annual energy balances and require them to be met by synthesis from hydrogen or from biofuels. Unlike for the other modes, we do not assume a "winning" drive train for road transport. Instead, we calculate the distance traveled by all vehicles in each country and use this distance as the road transport demand in the model. Annual vehicle mileage is based on JRC-IDEES and is split into motorcycles, passenger cars, buses, light-duty commercial vehicles, and heavy-duty freight vehicles. Vehicle mileage is then transformed back to energy demand based on the efficiency of different drivetrains. We use the 25th percentile of all countries' vehicle energy consumption, as given by JRC-IDEES for the year 2015 to define vehicle efficiency. This represents a convergence on higher efficiency of vehicles in all countries in Europe, but not an improvement in countries with already efficient vehicle fleets.

Only light-duty (including passenger and commercial) electric vehicle and passenger rail demands are assumed to have hourly profiles impacting energy delivery; all other demands, which are for liquid fuels, must be met on an annual basis. Rail electricity profiles are taken from th Demand for Energy Services, Supply and Transmission in EuropE (DESSTINEE) demand model.[39] Electric vehicles are limited in the allowed energy delivery per hour based on the number of vehicles connected to the grid at any given time. We generate this plug-in profile using RAMP-Mobility,[54] an extension of the open-source RAMP engine mentioned above.[52] The available charge capacity of plugged-in vehicles is based on the number of vehicles and an average battery size.[55] This method allows the model to decide when to charge cars (smart charging), but ensures that it is not unrealistic in the frequency of charging throughout the year. That is, it cannot choose to charge all vehicles in one week of the year. In addition, we enforce that any electric vehicle demand must be balanced on a monthly basis, using demands derived from RAMP-Mobility.

We generate industry sector demands by considering each industry subsector separately. We assume most process heat can be met electrically[56] and use JRC-IDEES electrical efficiency for meeting these demands to convert process demands to demand for electricity. Where JRC-IDEES has no electrical alternative for a process, such as for some steam processes, we retain methane demands in our model. We also mitigate the consumption of fossil fuels as feedstock to industrial processes in the iron & steel and chemicals subsectors, since these feedstocks contribute to a large proportion of these subsectors' emissions.[56,57] In iron & steel, we replace the conventional route of production (blast/basic oxygen furnace) with a completely electrified process: hydrogen-fueled direct reduction of iron followed by electric arc furnaces. To produce "high value chemicals" for plastics, we assume a feedstock of methanol to replace fossil fuels,[58] which can be synthesized from hydrogen or from biofuels. In addition, we replace natural gas as a feedstock for ammonia and urea. To change processes in iron & steel and chemicals industries, we use demands for final products (steel, high value chemicals, etc.) from JRC-IDEES and calculate demands for hydrogen, CO_2, and direct electricity based on estimated process efficiencies.

The final energy-consuming sectors given by the Eurostat annual energy balances not covered by any of the previous subsections are agriculture & forestry, fishing, and "not elsewhere specified." These sectors account for approximately 2.5% of total European annual energy demand. We assume all oil consumption is for transport

and add it to annual demand for heavy-duty vehicles (''agriculture & forestry'' and non-kerosene use in ''not elsewhere specified''), shipping (''fishing''), and aviation (kerosene in ''not elsewhere specified''). All other non-electricity consumption is assumed to be for building heating applications, and therefore added to annual commercial building heat consumption.

Data gaps exist in demand, from both Eurostat and JRC-IDEES databases. In particular, JRC-IDEES does not extend beyond 2015 and only includes the EU28. Furthermore, Eurostat has limited or no data for some Balkan countries, Switzerland, and Iceland. Gap-filling is undertaken first by blending the JRC-IDEES and Eurostat dataset (e.g., demand per unit consumption from JRC-IDEES is applied to Eurostat energy consumption data in the years 2016–2018). If no data exist, data are interpolated in time and are based on neighboring countries in space. For Iceland, other Nordic countries act as the basis for data. For Switzerland, Germany, Austria, France, and Italy are the basis, although we also use specific data from Swiss government statistics. Similarly, for Balkan countries, direct neighbors are used. In all instances, demand *intensities* are used, not absolute demand. These intensities are then scaled based on country-specific data that are available, e.g., population, gross value added (GVA), and demand in other years. All of the resulting assumptions and data are freely accessible in the repositories linked to above.

Supply data

Hourly wind farm and PV capacity factors are based on bias-corrected simulations using MERRA-2.[59,60] We set upper limits on wind and PV capacities based on physical limits set by existing land use and infrastructure, following the bottom-up method described in Tröndle et al.[32] Hourly hydropower capacity factors are based on ERA-5 runoff data, scaled to annual production of hydropower in each country. We assume hydropower capacities to be fixed, since expected future growth in Europe is limited.[61] These capacities, for dams, run-of-river, and pumped hydro, and their regional distributions are all taken from version 7 of the JRC hydropower database.[62] The available municipal waste supply is based on today's consumption of municipal waste for energy, as defined by Eurostat. We do not assume any changes in municipal waste supply up to 2050. Nuclear capacity is limited according to possible ranges of future capacities from various sources. In most countries, this leads to no capacity, but in France and Finland, there is the opportunity for greater nuclear capacity than today. The nuclear capacity factor is limited to the range 75%–85% over the entire year, based on the capacity factor of the French nuclear fleet in 2018 and the worldwide median energy availability factor of nuclear reactors in 2006.[63] Biofuel supply is based on projected 2050 residual biofuel availability (i.e., those leftover from existing agricultural and forestry processes rather than those specifically cultivated for the energy sector) given by the ''medium'' availability scenario in Ruiz et al.[64] Hourly heat pump coefficients of performance (COPs) are based on gridded MERRA-2 air and ground temperature data scaled according to the average performance of new heat pumps sold by the manufacturer WAMAK,[65] with a correction factor of 0.8 to scale for in-use performance.[51] Gridded COP is then scaled to model regions using population and the proportion of ground-source (10%) and air-source (90%) heat pumps in the market today. To emulate the distributed nature of heat supply technologies, we introduce a constraint to ensure that the ratio of capacity investments is reflected in the share of each technology meeting demand in each hour. For instance, if 50% of heat supply capacity comes from heat pumps, they must also meet 50% ($\pm$2.5%) of heat demand in each hour. Synthetic fuels are an intermediate fuel to meet demand and can be derived from biofuels or electricity. The electricity route entails the generation of hydrogen by electrolysis

CellPress
OPEN ACCESS

and CO_2 by direct air capture. Both energy sources and be used to produce any of the modeled hydrocarbon energy carriers: methane, kerosene, diesel, and methanol. Technology costs and all non-hourly characteristics are almost entirely sourced from the Danish Energy Agency technology catalog,[66] using their 2050 projections, for internal consistency.

Regionalization

The model represents 35 European countries: the EU-27 (minus Malta), Norway, Iceland, Switzerland, Bosnia and Herzegovina, Montenegro, North Macedonia, Serbia, Albania, and the United Kingdom. We have modeled larger countries by sub-national regions, based on those developed within the European Commission Seventh Framework Programme project e-HIGHWAY 2050.[67] Sub-nationalization excludes Iceland, Ireland, Belgium, Luxembourg, the Netherlands, Estonia, Slovakia, Hungary, Slovenia, Croatia, Bosnia and Herzegovina, Montenegro, North Macedonia, Serbia, Albania, and Bulgaria. In total, there are 98 model regions. To regionalize sub-sectoral demands, different datasets have been used for different end-uses. Household and public and private passenger transport demand is regionalized using population. Commercial building and light-duty vehicle demand is regionalized using NUTS3 GVA from non-industrial subsectors (dataset: *nama_10r_3gva*, classifications G–U). Industry demand, including from freight transport, is regionalized depending on subsector. For industries with emitters registered in the EU emissions trading scheme (EU-ETS), we use the location and size of emitters in 2014 as a proxy for regional demand. For all other subsectors, we combine the number of employed individuals in each industry subsector (dataset: *sbs_r_nuts06_r2*) with quantity of loaded freight in each industry subsector (dataset: *road_go_na_rl3g*). We regionalize demand for aviation and shipping fuels based on average industry regionalization, on the assumption that these fuels would be synthetically generated in industrial regions, rather than exclusively at the point of consumption (e.g., major ports for shipping fuel).

Supply and storage capacities are regionalized for only a subset of technologies. Nuclear capacities can exist within a range, but the regions in which those capacities can be allocated is based on today's concentration of regional capacities. Hydropower capacities are regionalization based on the JRC hydropower database v7. Wind and solar capacity regional upper bounds are based on a bottom-up process, combining high-resolution technical eligibility criteria described in Tröndle et al.[32]

Spatial energy distribution

The initial high-voltage transmission network is based on the e-HIGHWAY 2050 project, in which a detailed analysis of the network was undertaken to produce simplified power capacities for each sub-region interconnection, as well as 48 planned/proposed new or upgraded connections described in the 2018 ENTSO-E ten year network development plan (TYNDP).[68] The capacity of these connections act as a lower bound that can be further expanded. Modeling grid expansion purely linearly may underestimate the cost of grid expansion. However, we mitigated this by differentiating the cost of additional grid expansion based on the actual costs of planned and recently completed projects, differentiated by distance and terrain. Inter-regional fuel distribution is represented by grouping all industry synthetic fuel demands into European-level demands that can be contributed to by any model region. We do not model distribution networks within model regions, nor do we consider costs associated with them.

SPORES

Thespatially explicit practically optimal results (SPORES) method by which we generate 441 equally feasible, near-optimal solutions is an advancement of the MGA method,[27,69] which we introduced in previous work.[21] Compared to other MGA approaches,[20,22,70]

SPORES is unique in making explicit the search for both technologically and spatially distinctive configurations of the energy system. Not only does the SPORES method look for equally feasible configurations in which, for instance, wind is deployed more than solar; it also explicitly looks for many feasible ways of spatially locating wind capacity at the sub-national scale, within roughly the same mix of deployed technologies. This proves particularly helpful to obtain configurations that may address regional equity and social acceptance concerns.[21]

The core of the SPORES approach, as applied in this work, is the following. First, we identify the cost-optimal solution as a starting point. Second, we assign an integer weight to every non-zero regional realization of technology capacity deployment in the cost-optimal solution, e.g., for wind deployed in Scotland. Third, we modify the model formulation such that the objective becomes the minimization of the sum of these integer weights. This means, in practice, that we push the model to avoid the deployment of those technology-region combinations, such as "wind in Scotland," which have previously been part of a feasible solution. Finally, we implement total annualized system cost as a global constraint, such that feasible solutions with different technology-region combinations can only be more expensive than the cost-optimal solution by given margin, which we set to 10% for the base model runs. The process can be repeated indefinitely, each time incrementally updating the weights based on the values assumed by variables in the new feasible configuration. For a subset of SPORES, we run this process up to ten times.

To systematically explore the solution space, we apply the SPORES approach at three levels in parallel: across all technologies at once, for specific technology groups, and for electricity supply technologies alongside a secondary, technology-explicit objective. The second and third levels move from a technology-agnostic search for alternatives to one in which specific technologies or groups of technologies are targeted for minimal deployment in the system. This is repeated systematically for all electricity, heat, fuel, and transport supply technologies, as well as for storage and transmission technologies. As acknowledged by other recent applications of MGA to energy system optimization models of large size,[20,22] the minimization of specific technologies within the selected cost relaxation margin allows to approximately capture the extreme points of the solution space. Our generation of a relatively large batch of SPORES for each of these extreme points ensures that we also find alternatives further inside the solution space. For instance, we might find alternatives in which deployment of wind is always minimized, but in which different technologies replace wind generation, or these technologies are distributed differently at the sub-national scale. For a system cost relaxation of 10%, we generate 14 SPORES with all technologies weighted equally, 119 with technologies targeted for spatial differentiation, and 308 with technologies targeted for minimization while considering electricity supply spatial differentiation. This leads to a total of 441 alternatives.

Sensitivity analyses

We run the cost-optimization of the energy system for a full year, then apply a 10% cost relaxation for our SPORES runs. The baseline year we use is 2018, but we also run the cost-optimal run for the years 2010–2017. Since they are computationally intensive, SPORES are not run for these years. Rather, we check that least-cost feasible configurations across weather years do not lie outside the feasible decision space already outlined by SPORES for the reference weather year. Sensitivity to weather years is prioritized over other possible uncertain input parameters, such as cost and demand profiles, based on previous studies that showed it to be the parameter to which high-resolution energy system models with high shares of variable renewable generation are most sensitive.[21,71,72]

We also run a sensitivity analysis on the impact of annual demand projections, using simulated trends for changes in service demands from the models DESSTINEE[39] and high-efficiency buildings (HEB)[40] to update demands in the baseline year 2018 model. For the baseline year 2018, we also test a subset of SPORE runs with 5% and 15% relaxations. 120 SPORES are generated in total per cost relaxation sensitivity run, and 73 SPORES are generated in the demand projection sensitivity run. We compare the results for the equivalent runs in the baseline (10% relaxation) run. These SPORES focus on excluding specific technology groups while exploring spatial diversity of primary electricity supply. The results from the sensitivity analyses are in Note S2.

Primary energy supply

We calculate primary energy supply according to the methodology set out in the Eurostat annual energy balances. This entails the use of lower heating value for fossil fuels, biofuels, and non-renewable waste. For renewable supply, including hydropower, wind, and solar, the primary energy supply is the electricity produced by these technologies. For nuclear power, we follow the Eurostat convention of converting the electricity generated back to the heat provided by the fission process, using our input plant efficiency of 40%. We do not include "ambient heat," which is the heat extracted from the atmosphere when operating heat pumps. We also do not consider "Heat" (H8000), which is the heat made available from district heating systems; rather, we consider the primary energy into those systems (e.g., municipal waste). We group technologies into broader categories than those given by Eurostat's Standard Code List,[73] and provide human-readable names to the codes, as follows: electricity (*E7000*), other fossils (*C0000X0350-0370*, *C0350-0370*, *P1000*), oil (All codes starting in *O4000*, and *S2000*), natural gas (*G3000*), waste (*W6100_6220*), nuclear heat (*N900H*), renewables (all codes starting in *RA*[1–5]), biofuels (all codes starting in *R5*, and *W6210*).

SUPPLEMENTAL INFORMATION

Supplemental information can be found online at https://doi.org/10.1016/j.joule.2022.05.009.

ACKNOWLEDGMENTS

This work has received funding from the SENTINEL project of the European Union's Horizon 2020 research and innovation program under grant agreement no. 837089, from the ECEMF project of the European Union's Horizon 2020 research and innovation program under grant agreement no. 101022622, and from the Swiss Federal Office of Energy's "SWEET" program and performed in the PATHFNDR consortium. Model runs were performed on the ETH Euler cluster.

AUTHOR CONTRIBUTIONS

B.P., F.L., and S.P. designed the research approach and wrote the paper. B.P. and F.L. gathered the data. B.P. built and ran the models, analyzed the results, and created the figures.

DECLARATION OF INTERESTS

The authors declare no competing interests.

Received: January 11, 2022
Revised: March 21, 2022
Accepted: May 17, 2022
Published: June 8, 2022

REFERENCES

1. Tröndle, T., Lilliestam, J., Marelli, S., and Pfenninger, S. (2020). Trade-offs between geographic scale, cost, and infrastructure requirements for fully renewable electricity in Europe. Joule *4*, 1929–1948. ISSN 2542-4351. https://doi.org/10.1016/j.joule.2020.07.018.

2. Brown, T., Schlachtberger, D., Kies, A., Schramm, S., and Greiner, M. (2018). Synergies of sector coupling and transmission reinforcement in a cost-optimised, highly renewable European energy system. Energy *160*, 720–739. ISSN 0360-5442. https://doi.org/10.1016/j.energy.2018.06.222.

3. Heide, D., von Bremen, L., Greiner, M., Hoffmann, C., Speckmann, M., and Bofinger, S. (2010). Seasonal optimal mix of wind and solar power in a future, highly renewable Europe. Renew. Energy *35*, 2483–2489. ISSN 0960-1481. https://doi.org/10.1016/j.renene.2010.03.012.

4. Hansen, K., Breyer, C., and Lund, H. (2019). Status and perspectives on 100% renewable energy systems. Energy *175*, 471–480. ISSN 0360-5442. https://doi.org/10.1016/j.energy.2019.03.092.

5. Bogdanov, D., Ram, M., Aghahosseini, A., Gulagi, A., Oyewo, A.S., Child, M., Caldera, U., Sadovskaia, K., Farfan, J., De Souza Noel Simas Barbosa, L., et al. (2021). Low-cost renewable electricity as the key driver of the global energy transition towards sustainability. Energy *227*, 120467. ISSN 0360-5442. https://doi.org/10.1016/j.energy.2021.120467.

6. IRENA (2020). Renewable Power Generation Costs in 2019 (International Renewable Energy Agency). ISBN 978-92-9260-244-4.

7. Jansen, M., Staffell, I., Kitzing, L., Quoilin, S., Wiggelinkhuizen, E., Bulder, B., Riepin, I., and Müsgens, F. (2020). Offshore wind competitiveness in mature markets without subsidy. Nat. Energy *5*, 614–622. ISSN 2058-7546. https://doi.org/10.1038/s41560-020-0661-2.

8. Grant, N., Hawkes, A., Napp, T., and Gambhir, A. (2021). Cost reductions in renewables can substantially erode the value of carbon capture and storage in mitigation pathways. One Earth *4*, 1588–1601. ISSN 2590-3322. https://doi.org/10.1016/j.oneear.2021.10.024.

9. Hörsch, J., Hofmann, F., Schlachtberger, D., and Brown, T. (2018). PyPSA-Eur: an open optimisation model of the European transmission system. Energy Strategy Rev *22*, 207–215. ISSN 2211-467X. https://doi.org/10.1016/j.esr.2018.08.012.

10. Schlott, M., Kies, A., Brown, T., Schramm, S., and Greiner, M. (2018). The impact of climate change on a cost-optimal highly renewable European electricity network. Appl. Energy *230*, 1645–1659. ISSN 0306-2619. https://doi.org/10.1016/j.apenergy.2018.09.084.

11. Zappa, W., Junginger, M., and van den Broek, M. (2019). Is a 100% renewable European power system feasible by 2050? Appl. Energy *233–234*, 1027–1050. ISSN 0306-2619. https://doi.org/10.1016/j.apenergy.2018.08.109.

12. Victoria, M., Zhu, K., Brown, T., Andresen, G.B., and Greiner, M. (2020). Early decarbonisation of the European energy system pays off. Nat. Commun. *11*, 6223. ISSN 2041-1723. https://doi.org/10.1038/s41467-020-20015-4.

13. Davis, S.J., Lewis, N.S., Shaner, M., Aggarwal, S., Arent, D., Azevedo, I.L., Benson, S.M., Bradley, T., Brouwer, J., Chiang, Y.-M., et al. (2018). Net-zero emissions energy systems. Science *360*, eaas9793. https://doi.org/10.1126/science.aas9793.

14. IEA (2021). Net Zero by 2050. Technical report (International Energy Agency). https://www.iea.org/reports/net-zero-by-2050.

15. Victoria, M., Zhu, K., Brown, T., Andresen, G.B., and Greiner, M. (2019). The role of storage technologies throughout the decarbonisation of the sector-coupled European energy system. Energy Convers. Manag. *201*, 111977. ISSN 0196-8904. https://doi.org/10.1016/j.enconman.2019.111977.

16. Collins, S., Deane, J.P., Poncelet, K., Panos, E., Pietzcker, R.C., Delarue, E., and Ó Gallachóir, B.P.Ó. (2017). Integrating short term variations of the power system into integrated energy system models: a methodological review. Renew. Sustain. Energy Rev. *76*, 839–856. ISSN 1364-0321. https://doi.org/10.1016/j.rser.2017.03.090.

17. Pietzcker, R.C., Ueckerdt, F., Carrara, S., de Boer, H.S., Després, J., Fujimori, S., Johnson, N., Kitous, A., Scholz, Y., Sullivan, P., and Luderer, G. (2017). System integration of wind and solar power in integrated assessment models: a cross-model evaluation of new approaches. Energy Econ *64*, 583–599. ISSN 0140-9883. https://doi.org/10.1016/j.eneco.2016.11.018.

18. Rogelj, J., Shindell, D., Jiang, K., Fifita, S., Forster, P., Ginzburg, V., Handa, C., Kheshgi, H., Kobayashi, S., Kriegler, E., et al. (2018). Mitigation pathways compatible with 1.5°C in the context of sustainable development. In Global Warming of 1.5°C. An IPCC Special Report on the Impacts of Global Warming of 1.5°C above Pre-industrial Levels and Related Global Greenhouse Gas Emission Pathways, in the Context of Strengthening the Global Response to the Threat of Climate Change, Sustainable Development, and Efforts to Eradicate Poverty, V. Masson-Delmotte, P. Zhai, H.-O. Pörtner, D. Roberts, J. Skea, P.R. Shukla, A. Pirani, W. Moufouma-Okia, C. Péan, and R. Pidcock, et al., eds. (Intergovernmental Panel on Climate Change (IPCC)).

19. Tsiropoulos, I., Nijs, W., Tarvydas, D., and Ruiz, P. (2020). Towards Net-Zero Emissions in the EU Energy System by 2050 – Insights from Scenarios in Line with the 2030 and 2050 Ambitions of the European Green Deal (Publications Office of the European Union). Technical Report EUR 29981 EN.

20. Neumann, F., and Brown, T. (2021). The near-optimal feasible space of a renewable power system model. Electr. Power Syst. Res. *190*, 106690. ISSN 0378-7796. https://doi.org/10.1016/j.epsr.2020.106690. https://www.sciencedirect.com/science/article/pii/S0378779620304934.

21. Lombardi, F., Pickering, B., Colombo, E., and Pfenninger, S. (2020). Policy decision support for renewables deployment through spatially explicit practically optimal alternatives. Joule *4351*, 2542–4351. ISSN 2542-4785. https://doi.org/10.1016/j.joule.2020.08.002.

22. Pedersen, T.T., Victoria, M., Rasmussen, M.G., and Andresen, G.B. (2021). Modeling all alternative solutions for highly renewable energy systems. Energy *234*, 121294. ISSN 0360-5442. https://doi.org/10.1016/j.energy.2021.121294. https://www.sciencedirect.com/science/article/pii/S0360544221015425.

23. Heuberger, C.F., Staffell, I., Shah, N., and Mac Dowell, N. (2018). Impact of myopic decision-making and disruptive events in power systems planning. Nat. Energy *3*, 634–640. ISSN 2058-7546. https://doi.org/10.1038/s41560-018-0159-3. https://www.nature.com/articles/s41560-018-0159-3.

24. Staffell, I., Scamman, D., Velazquez Abad, A., Balcombe, P., Dodds, P.E., Ekins, P., Shah, N., and Ward, K.R. (2019). The role of hydrogen and fuel cells in the global energy system. Energy Environ. Sci. *12*, 463–491. https://doi.org/10.1039/C8EE01157E.

25. van Renssen, S. (2020). The hydrogen solution? Nat. Clim. Change *10*, 799–801. ISSN 1758-6798. https://doi.org/10.1038/s41558-020-0891-0. https://www.nature.com/articles/s41558-020-0891-0.

26. Jahangir Samet, M., Liimatainen, H., van Vliet, O.P.R., and Pöllänen, M. (2021). Road freight transport electrification potential by using battery electric trucks in Finland and Switzerland. Energies *14*, 823. https://doi.org/10.3390/en14040823. https://www.mdpi.com/1996-1073/14/4/823.

27. DeCarolis, J.F. (2011). Using modeling to generate alternatives (MGA) to expand our thinking on energy futures. Energy Econ *33*, 145–152. ISSN 0140-9883. https://doi.org/10.1016/j.eneco.2010.05.002. https://www.sciencedirect.com/science/article/pii/S0140988310000721.

28. Tong, D., Farnham, D.J., Duan, L., Zhang, Q., Lewis, N.S., Caldeira, K., and Davis, S.J. (2021). Geophysical constraints on the reliability of solar and wind power worldwide. Nat. Commun. *12*, 6146. ISSN 2041-1723. https://doi.org/10.1038/s41467-021-26355-z.

29. Arbabzadeh, M., Sioshansi, R., Johnson, J.X., and Keoleian, G.A. (2019). The role of energy storage in deep decarbonization of electricity production. Nat. Commun. *10*, 3413. ISSN 2041-1723. https://doi.org/10.1038/s41467-019-11161-5.

30. Dowling, J.A., Rinaldi, K.Z., Ruggles, T.H., Davis, S.J., Yuan, Mengyao, Tong, F., Lewis, N.S., and Caldeira, K. (2020). Role of long-duration energy storage in variable renewable electricity systems. Joule *4*, 1907–1928. ISSN 2542-4351. https://doi.org/10.1016/j.joule.2020.07.007.

31. Schlachtberger, D.P., Brown, T., Schäfer, M., Schramm, S., and Greiner, M. (2018). Cost optimal scenarios of a future highly renewable European electricity system: exploring the influence of weather data, cost parameters and policy constraints. Energy *163*, 100–114. ISSN 0360-5442. https://doi.org/10.1016/j.energy.2018.08.070.

32. Tröndle, T., Pfenninger, S., and Lilliestam, J. (2019). Home-made or imported: on the possibility for renewable electricity autarky on all scales in Europe. Energy Strategy Rev *26*, 100388. ISSN 2211-467X. https://doi.org/10.1016/j.esr.2019.100388.

33. Jenkins, J.D., Zhou, Z., Ponciroli, R., Vilim, R.B., Ganda, F., de Sisternes, F., and Botterud, A. (2018). The benefits of nuclear flexibility in power system operations with renewable energy. Appl. Energy *222*, 872–884. ISSN 0306-2619. https://doi.org/10.1016/j.apenergy.2018.03.002.

34. Capros, P., Kannavou, M., Evangelopoulou, S., Petropoulos, A., Siskos, P., Tasios, N., Zazias, G., and DeVita, A. (2018). Outlook of the EU energy system up to 2050: the case of scenarios prepared for European Commission's "clean energy for all Europeans" package using the PRIMES model. Energy Strategy Rev *22*, 255–263. ISSN 2211-467X. https://doi.org/10.1016/j.esr.2018.06.009.

35. Sepulveda, N.A., Jenkins, J.D., de Sisternes, F.J., and Lester, R.K. (2018). The role of firm low-carbon electricity resources in deep decarbonization of power generation. Joule *2*, 2403–2420. ISSN 2542-4351. https://doi.org/10.1016/j.joule.2018.08.006.

36. Pleßmann, G., and Blechinger, P. (2017). How to meet EU GHG emission reduction targets? a model based decarbonization pathway for Europe's electricity supply system until 2050. Energy Strategy Rev *15*, 19–32. https://doi.org/10.1016/j.esr.2016.11.003.

37. Golombek, R., Lind, A., Ringkjøb, H.-K., and Seljom, P. (2022). The role of transmission and energy storage in European decarbonization towards 2050. Energy *239*, 122159. ISSN 0360-5442. https://doi.org/10.1016/j.energy.2021.122159.

38. Jacobson, M.Z. (2021). The cost of grid stability with 100 % clean, renewable energy for all purposes when countries are isolated versus interconnected. Renew. Energy *179*, 1065–1075. ISSN 0960-1481. https://doi.org/10.1016/j.renene.2021.07.115.

39. Boßmann, T., and Staffell, I. (2015). The shape of future electricity demand: exploring load curves in 2050s Germany and Britain. Energy *90*, 1317–1333. ISSN 0360-5442. https://doi.org/10.1016/j.energy.2015.06.082.

40. Güneralp, B., Zhou, Y., Ürge-Vorsatz, D., Gupta, M., Yu, S., Patel, P.L., Fragkias, M., Li, Xiaoma, and Seto, K.C. (2017). Global scenarios of urban density and its impacts on building energy use through 2050. Proc. Natl. Acad. Sci. USA *114*, 8945–8950. ISSN 0027-8424. https://doi.org/10.1073/pnas.1606035114.

41. Craig, M.T., Wohland, J., Stoop, L.P., Kies, A., Pickering, B., Bloomfield, H., et al. (2022). Overcoming the disconnect between energy system and climate modeling. Joule. https://doi.org/10.1016/j.joule.2022.05.010.

42. Cherp, A., Vinichenko, V., Tosun, J., Gordon, J.A., and Jewell, J. (2021). National growth dynamics of wind and solar power compared to the growth required for global climate targets. Nat. Energy *6*, 742–754. ISSN 2058-7546. https://doi.org/10.1038/s41560-021-00863-0.

43. Süsser, D., Ceglarz, A., Gaschnig, H., Stavrakas, V., Flamos, A., Giannakidis, G., and Lilliestam, J. (2021). Model-based policymaking or policy-based modelling? How energy models and energy policy interact. Energy Res. Soc. Sci. *75*, 101984. ISSN 2214-6296. https://doi.org/10.1016/j.erss.2021.101984.

44. Pfenninger, S., and Pickering, B. (2018). Calliope: a multi-scale energy systems modelling framework. J. Open Source Software *3*, 825. https://doi.org/10.21105/joss.00825.

45. European Commission (2020). Eurostat database. https://ec.europa.eu/eurostat/data/database.

46. Mantzos, L., Wiesenthal, T., Matei, N.A., Tchung-Ming, S., Rozsai, Mate, Russ, P., and Soria Ramirez, A. (2017). JRC-IDEES: Integrated Database of the European Energy Sector: Methodological Note. Technical Report (Joint Research Centre) (Seville site).

47. Wiese, F., Schlecht, I., Bunke, W.-D., Gerbaulet, C., Hirth, L., Jahn, M., Kunz, F., Lorenz, C., Mühlenpfordt, J., Reimann, J., and Schill, W.-P. (2019). Open power system data—frictionless data for electricity system modelling. Appl. Energy *236*, 401–409. ISSN 0306-2619. https://doi.org/10.1016/j.apenergy.2018.11.097.

48. European Commission; Directorate-General for Climate Action; Directorate-General for Energy; Directorate-General for Mobility, Transport, De Vita, A., Capros, P., Paroussos, L., Fragkiadakis, K., Karkatsoulis, P., Höglund-Isaksson, L., Winiwarter, W., et al. (2021). EU Reference Scenario 2020: Energy, Transport and GHG Emissions: Trends to 2050 (Publications Office). https://doi.org/10.2833/35750.

49. van der Wiel, K., Stoop, L.P., van Zuijlen, B.R.H., Blackport, R., van den Broek, M.A., and Selten, F.M. (2019a). Meteorological conditions leading to extreme low variable renewable energy production and extreme high energy shortfall. Renew. Sustain. Energy Rev. *111*, 261–275. ISSN 1364-0321. https://doi.org/10.1016/j.rser.2019.04.065.

50. van der Wiel, K., Bloomfield, H.C., Lee, R.W., Stoop, L.P., Blackport, R., Screen, J.A., and Selten, F.M. (2019b). The influence of weather regimes on European renewable energy production and demand. Environ. Res. Lett. *14*, 094010. ISSN 1748-9326. https://doi.org/10.1088/1748-9326/ab38d3.

51. Ruhnau, O., Hirth, L., and Praktiknjo, A. (2019). Time series of heat demand and heat pump efficiency for energy system modeling. Sci. Data *6*, 189. ISSN 2052-4463. https://doi.org/10.1038/s41597-019-0199-y.

52. Lombardi, F., Balderrama, S., Quoilin, S., and Colombo, E. (2019a). Generating high-resolution multi-energy load profiles for remote areas with an open-source stochastic model. Energy *177*, 433–444. ISSN 0360-5442. https://doi.org/10.1016/j.energy.2019.04.097.

53. Lombardi, F., Rocco, M.V., and Colombo, E. (2019b). A multi-layer energy modelling methodology to assess the impact of heat-electricity integration strategies: the case of the residential cooking sector in Italy. Energy *170*, 1249–1260. ISSN 0360-5442. https://doi.org/10.1016/j.energy.2019.01.004.

54. Mangipinto, A., Lombardi, F., Sanvito, F.D., Pavičević, M., Quoilin, S., and Colombo, E. (2022). Impact of mass-scale deployment of electric vehicles and benefits of smart charging across all European countries. Appl. Energy *312*, 118676. ISSN 0306-2619. https://doi.org/10.1016/j.apenergy.2022.118676.

55. The European Council for Automotive. (2019). R&D. Battery Requirements for Future Automotive Applications. Technical Report (EUCAR).

56. Madeddu, S., Ueckerdt, F., Pehl, M., Peterseim, J., Lord, M., Kumar, K.A., Krüger, C., and Luderer, G. (2020). The CO_2 reduction potential for the European industry via direct electrification of heat supply (power-to-heat). Environ. Res. Lett. *15*. https://doi.org/10.1088/1748-9326/abbd02.

57. Suopajärvi, H., Umeki, K., Mousa, E., Hedayati, A., Romar, H., Kemppainen, A., Wang, C., Phounglamcheik, A., Tuomikoski, S., Norberg, N., et al. (2018). Use of biomass in integrated steelmaking—status quo, future needs and comparison to other low-CO_2 steel production technologies. Appl. Energy *213*, 384–407. ISSN 0306-2619. https://doi.org/10.1016/j.apenergy.2018.01.060.

58. Bazzanella, A., and Ausfelder, F. (2017). Low Carbon Energy and Feedstock for the European Chemical Industry (DECHEMA, Gesellschaft für Chemische Technik und Biotechnologie eV).

59. Pfenninger, S., and Staffell, I. (2016). Long-term patterns of European PV output using 30 years of validated hourly reanalysis and satellite data. Energy *114*, 1251–1265. ISSN 0360-5442. https://doi.org/10.1016/j.energy.2016.08.060.

60. Staffell, I., and Pfenninger, S. (2016). Using bias-corrected reanalysis to simulate current and future wind power output. Energy *114*, 1224–1239. ISSN 0360-5442. https://doi.org/10.1016/j.energy.2016.08.068.

61. European Commission (2013). Technology Map of the European Strategic Energy Technology Plan (Publications Office). ISBN 9789279347214. https://doi.org/10.2790/9986.

62. De Felice, M., and Kavvadias, K. (2020). Energy-Modelling-Toolkit/Hydro-power-Database: JRC Hydro-power Database. Release 07 (Zenodo).

63. Curley, G.M., and Mandula, J. (2008). Thermal Generating Plant Unavailability Factors and Availability Statistics (World Energy Council). Technical Report.

64. Ruiz, P., Sgobbi, A., Nijs, W., Thiel, C., Dalla Longa, F., Kober, T., Elbersen, B., and Hengeveld, G. (2015). The JRC-EU-TIMES model. Bioenergy potentials for EU and neighbouring countries (JRC Science for Policy Report). (European Commission).

65. WAMAK (2020). Heat pump catalogue. https://www.wamak.eu/en/heat-pumps.

66. Danish Energy Agency (2020). Technology data. https://ens.dk/en/our-services/projections-and-models/technology-data.

67. Anderski, T., Surmann, Y., Stemmer, S., Grisey, N., Momot, E., Leger, A.-C., Betraoui, B., and van Roy, P. (2014). European cluster model of

the Pan-European transmission grid. Deliverable D2, 2. e-HIGHWAY 2050.

68. ENTSOE. TYNDP (2018). Project Sheets, 2018. https://tyndp.entsoe.eu/tyndp2018/projects/projects.

69. Brill, E.D. (1979). The use of optimization models in public-sector planning. Manag. Sci. *25*, 413–422. ISSN 0025-1909. https://doi.org/10.1287/mnsc.25.5.413. https://pubsonline.informs.org/doi/abs/10.1287/mnsc.25.5.413.

70. Sasse, J.-P., and Trutnevyte, E. (2020). Regional impacts of electricity system transition in Central Europe until 2035. Nat. Commun. *11*, 4972. ISSN 2041-1723. https://doi.org/10.1038/s41467-020-18812-y. https://www.nature.com/articles/s41467-020-18812-y.

71. Bloomfield, H.C., Gonzalez, P.L.M., Lundquist, J.K., Stoop, L.P., Browell, J., Dargaville, R., De Felice, M., Gruber, K., Hilbers, A., Kies, A., et al. (2021). The importance of weather and climate to energy systems: a workshop on next generation challenges in energy–climate modeling. Bull. Am. Meteorol. Soc. *102*, E159–E167. ISSN 0003-0007, 1520-0477. https://doi.org/10.1175/BAMS-D-20-0256.1. https://journals.ametsoc.org/view/journals/bams/102/1/BAMS-D-20-0256.1.xml.

72. Pfenninger, S. (2017). Dealing with multiple decades of hourly wind and PV time series in energy models: a comparison of methods to reduce time resolution and the planning implications of inter-annual variability. Appl. Energy *197*, 1–13. ISSN 0306-2619. https://doi.org/10.1016/j.apenergy.2017.03.051.

73. European Commission (2021). Eurostat reference and management of nomenclatures. https://ec.europa.eu/eurostat/ramon/.

Joule, Volume *6*

Supplemental information

Diversity of options to eliminate fossil fuels and reach carbon neutrality across the entire European energy system

Bryn Pickering, Francesco Lombardi, and Stefan Pfenninger

Supplemental Experimental Procedures

Note S1: Sector-Coupled Euro-Calliope model

The model we use in this study is an extension of Euro-Calliope v1.0[1], to include all energy-consuming sectors in Europe. Euro-Calliope v1.0 includes only the power sector and only current electricity loads. This section will detail the process of extending Euro-Calliope to include each of the following sectors: household and commercial heat, passenger and freight transport, industry process heat and feedstocks, and energy consumption in all other sectors including agriculture. For each sector, demand and supply technology data has been acquired from various sources and combined in an automated workflow, openly available on GitHub. A data source overview is given in Table S1, for those data accessed for the purpose of adding new sectors. For a detailed understanding of data sources used in Euro-Calliope v1.0, refer to Tröndle et al.[2].

Table S1: Primary data sources used in Euro-Calliope model development. Resolution is given in the context of the Nomenclature of Territorial Units for Statistics (NUTS). Eurostat dataset codes are given in parentheses.

Source name	Data accessed	Resolution	Use of data	Sectors affected
**Eurostat**	Annual energy balances (*nrg_bal_c*)	NUTS0	Energy data in all subsectors	All
	Annual household energy end-uses (*nrg_d_hhq*)	NUTS0	Household end-use energy consumption	Household heat and electricity
	freight loading (*road_go_na_rl3g*)	NUTS3	Sub-regional disaggregation of industry demand	Industry
	employees by subsector (*sbs_r_nuts06_r2*)	NUTS2		
	dwelling number and types (*cens_11dwob_r3*)	NUTS3	Heat demand generation	Building space and water heat
	Gross value added by commercial subsector (*nama_10r_3gva*)	NUTS3	Sub-national disaggregation of commercial demand	Commercial building and transport
**Joint Research Centre (JRC)**	JRC IDEES[3]	NUTS0	Attribution of consumed resources per subsector to end-uses	All
	JRC open power plant database[4]	Site-specific	Location of existing conventional power supply technologies	Power
**Swiss federal office for energy (SFOE)**	Swiss equivalent of Eurostat data	NUTS0	Energy data in all subsectors, sub-regional demand disaggregation	All
**Danish energy agency**	Technology catalogue	N/A	Technology costs and operational characteristics	Heat, electricity, and renewable fuels

BUILDING HEAT SECTOR

We group heat demand in buildings into three end-uses: **space heat, hot water, and cooking**. These groups match the Eurostat household end-use categorisation, national data for which became available in 2020 (nrg_d_hhq). This data has been used to assign the consumption of fuels to different end-uses. We infer building heat demand in commercial and industry sectors from the JRC IDEES database[3]. We then transform the consumption of fuels to a demand for heat by assuming technology efficiencies of heating technologies including boilers and direct electric heaters (see Table S2). These efficiencies are consistent with those used in the Sector-Coupled Euro-Calliope for the respective heat supply technologies. Heat pumps are a special case, since heat demand can be calculated using the consumption of ambient heat: demand = ambient heat consumption + electricity consumption. We use these annual water and space heat demands to scale hourly demand profiles produced using the methods implemented for the When2Heat database[5], updated to account for (a) all Euro-Calliope countries and (b) the sub-national distribution of single- to multi-family homes across Europe, according to the Eurostat database of dwellings (cens_11dwob_r3). We generate cooking heat demand profiles using a bottom-up stochastic modelling. Our approach extends the open-source RAMP engine [6], developed and validated in previous work[7] with application to Italy, to stochastically model demand in all European countries.

To meet these demands, we define a range of new technologies in the model, key data for which can be found in Table S3. We source most data for these from the Danish Energy Agency Technology Catalogue[8]. Again, heat pumps are a special case, since their performance is weather-dependent. Our heat pump coefficients of performance (COPs) are based on a catalogue of 78 heat pumps provided by the manufacturer WAMAK. The WAMAK heat pump performance data represents state-of-the art technology and generally fits that given by previous studies (Figure S1)[5,9,10]. Following Ruhnau et al.[5], we assume COP to be 80% of published performance. The WAMAK catalogue data also includes actual technology heat delivery capacity, relative to nominal capacity (Figure S2). That is, the nominal capacity in which one invests is not the actual capacity that is realised, which instead depends on sink and source temperatures. We describe heat pumps using hourly COP and capacity variation; both timeseries rely on hourly temperature data from the MERRA-2 reanalysis[11] [a].

Table S2: Heat technology efficiencies used to translate consumed energy resources into demand for end-use heat.

	Technology	Efficiency
**Space and water heating**	Gas (natural gas, biogas)	0.97 [12]
	Petroleum products	0.9 [12]
	Solid fossil fuels	0.8 (assumed the same as for solid biofuels)
	Solid biofuels	0.8 [12–14]
	Solar thermal	1.0 (as per Eurostat energy balance methodology)
	Direct electric	1.0
**Cooking**	Gas (natural gas, biogas)	0.28 [15]
	Petroleum products	0.28 (assume same as gas)
	Solid fossil fuels	0.15 [16] scaled using [15]
	Solid biofuels	0.1 [16] scaled using [15]
	Direct electric	0.5 [15]

[a] Air-source heat pumps use surface air temperature while ground-source heat pumps use sub-surface temperature (tsoil5) – 5°C, to account for heat transfer to the ground loop brine

Table S3: Key data for heat supply technologies in the Sector-Coupled Euro-Calliope. Data is almost exclusively 2050 estimates from the Danish Energy Agency technology catalogue[8]. Greater detail can be found in the model implementation.

Technology	Energy input	Efficiency	Capital cost (EUR2015/kW)
methane boiler	Methane	97%	172
biofuel boiler	Biofuel	80%	445
air source heat pump	Electricity	Time varying COP	662
ground source heat pump	Electricity	Time varying COP	1100
solar thermal panels	Solar irradiance	Time varying efficiency	515
direct electric heaters	Electricity	100%	695
Combined heat and power plants	Waste / biofuel / methane	Depending on heat to power ratio	520 - 2783
hot water storage	Heat	0.01-0.02%/hour	3 (large-scale) – 410 (small-scale) EUR2015/kWh

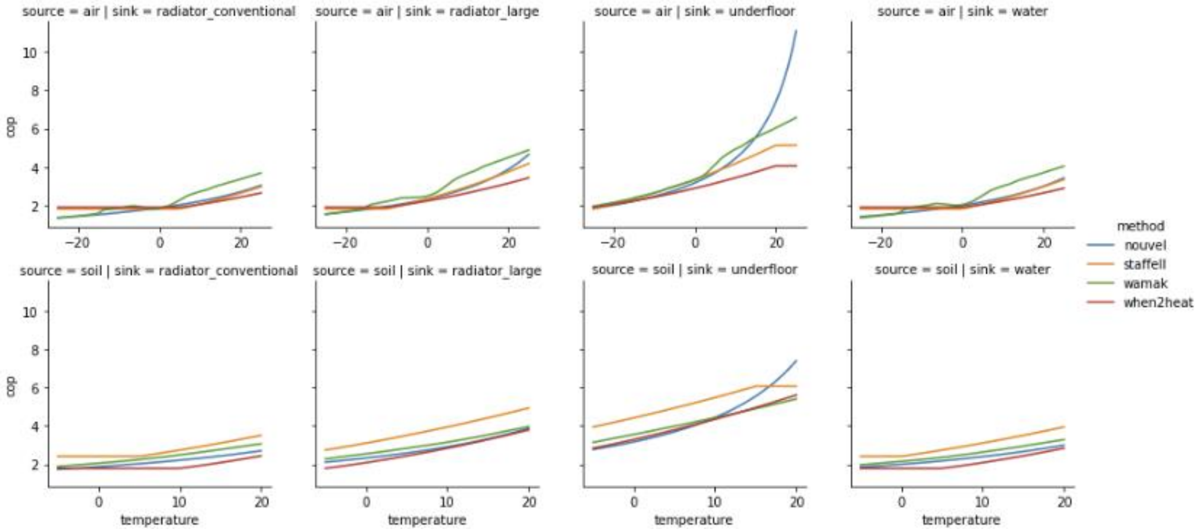

Figure S1: Comparison of heat pump coefficients of performance for different source and sink temperatures, as well as different technology types (air-source and ground-source heat pumps). Methods are named based on first authors of the respective studies[5,9,10], except WAMAK which is the manufacturer name from which performances were manually extracted from a catalogue of technologies.

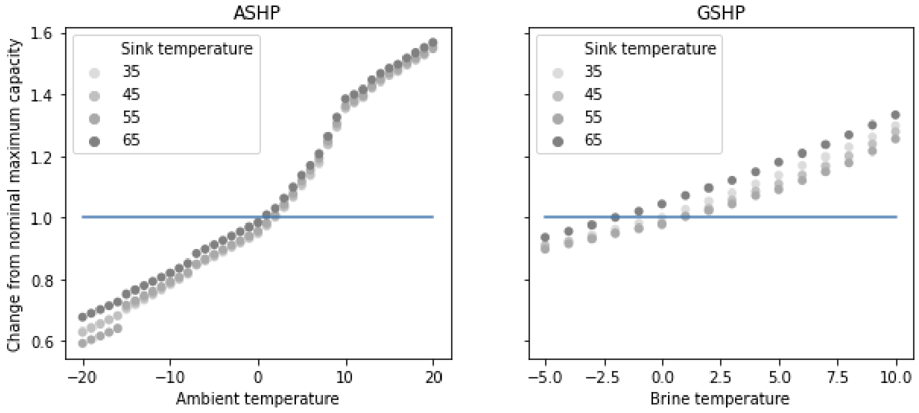

Figure S2: Change in heat pump delivery capacity as a function of source and sink temperature (ºC), based on the average performance of several heat pumps in the WAMAK catalogue of technologies. ASHP: air-source heat pump, GSHP: ground-source heat pump.

TRANSPORT SECTOR

The transport sector encompasses road, rail, air, and shipping. Electrification is only possible in some of these forms of transport, namely road and rail. In rail, we assume complete electrification is possible, such that all current fuel oil demand is replaced by direct electricity demand in 2050. The current consumption of fuel is taken from Eurostat, while the efficiency of different rail drive trains is taken from JRC IDEES. For air and shipping, we assume the opposite: there will be no electrification by 2050. Instead, the kerosene and diesel demand of these two forms of transport must be met by synthetic fuel generation. Accordingly, air and shipping (domestic and international) demands are taken directly from Eurostat. Unlike the other modes, we do not assume a 'winning' drive train for road transport. Instead, we calculate the distance travelled by all vehicles in each country and use this distance as the demand in the model. We use annual vehicle mileage from JRC IDEES, split into motorcycles, passenger cars, busses, light-duty commercial vehicles, and heavy-duty freight vehicles. Vehicle mileage is then transformed back to energy demand based on the efficiency of different drivetrains. The energy consumption per unit distance data given in Table S4 are based on the 25th percentile of all countries' vehicle energy consumption, as given by JRC IDEES for the year 2015. This represents a convergence on higher efficiency of vehicles in all countries in Europe, but not an improvement in countries with existing efficient vehicle fleets.

We assume only light-duty electric vehicle and passenger rail demands have hourly profiles impacting energy delivery; all other demands must be met on an annual basis, since they are synthetic fuels. We take rail electricity demand profiles from the DESTINEE demand model[17]. Electric vehicles are limited in the allowed energy delivery per hour based on the number of vehicles connected to the grid at any given time. We generate this plug-in profile using RAMP-Mobility[18], an extension of the aforementioned open-source RAMP engine[6]. The result is that in some hours, as few as 70% of electric vehicles are plugged in (Figure S3). We base the available charge capacity of plugged-in vehicles on the number of vehicles and an average battery size[19]. The result of this is that if the model chooses to electrify half a region's vehicle fleet of 100 cars, then there will be a maximum of 50 cars plugged in, each with a battery of 0.08MWh. Thus, 4MWh of energy can be delivered to vehicles in that hour. This method allows the model to decide when to charge cars (smart charging), but ensures that it is not unrealistic in the frequency of charging throughout the year (i.e. it cannot choose to charge all vehicles in one week of the year). However, initial tests showed that this was still not sufficient to ensure "realistic" EV charging, with regions having little to no EV charging in January weeks (Figure S4). Accordingly, a bound on EV supply was applied to ensure charging demands are met in every month, using monthly EV electricity demand from RAMP-mobility (Figure S3).

*Table S4: Average vehicle fleet energy consumption by drivetrain (oil or electricity driven) and battery capacity of electric vehicles. Vehicle classes and energy consumption values are based on JRC IDEES; energy consumption is the 25th percentile of energy consumption across all JRC IDEES countries in 2015. Battery capacity is an average of values given by The European Council for Automotive R&D (EUCAR)[19]. *these values are not given by EUCAR, so are assumed.*

	**Energy consumption (MWh/million km)**		**Battery capacity (MWh)**
**Vehicle class**	*Oil*	*Electricity*	
Heavy duty vehicle	5140	N/A	0.2
Light duty vehicle	855	480	0.1*
Motorcycle	419	200	0.01*
Bus	6057	3248	0.2*
Passenger car	675	324	0.08

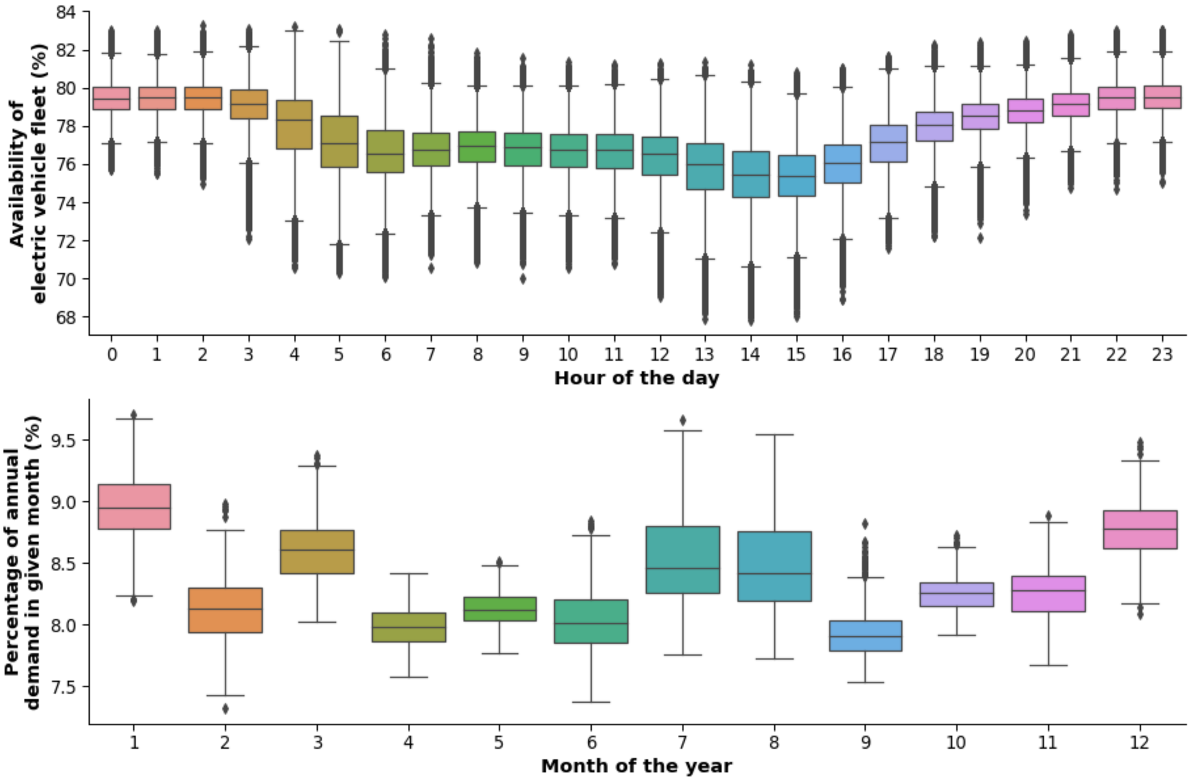

Figure S3: Overview of Euro-Calliope electric vehicle hourly plug-in schedule (top) and monthly demand (bottom), based on national stochastic profiles modelled in RAMP-mobility. Boxplots show the variation in availability per hour of the day (top) and month of the year (bottom), across years 2000-2018. Top: All hours are given in UTC+00:00, not local time.

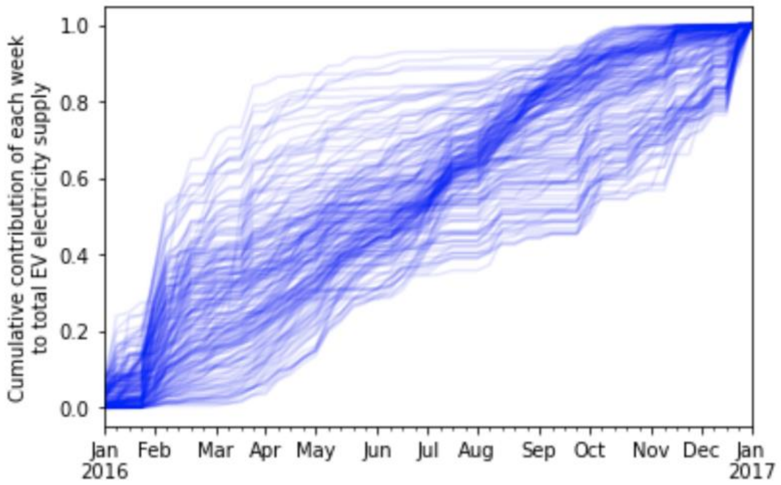

Figure S4: Normalised cumulative weekly EV charging across the 2016 weather year, based on results from optimising cost in the Euro-Calliope model with only a requirement to meet demand by the end of the year. Lines represent all 98 Euro-Calliope model regions and both electrified heavy-duty and light-duty vehicles. Each line has a low opacity, so high opacity areas indicate a greater degree of overlap.

INDUSTRY SECTOR

The industry sector has demand for different levels of heat (space heating, low temperature heat, and high temperature heat), for already electrified or electrifiable end-uses (namely, operation of machinery), and for consumption of energy resources as feedstock (such as oil for the production of base chemicals). There are no European statistics on the breakdown of fuel consumption that can be attributed to each end use, but some countries publish their own statistics. Countries with sufficiently disaggregated demand data are Germany, Austria, and the UK. Switzerland also has disaggregated data, for *either* the fuel consumption per industry subsector or the end use demand of industry in total, but not the fuel consumption per end use per industry subsector.

Both bottom-up and top-down approaches to understanding industrial energy demand have been undertaken to date[3,20–23]. Top-down modelling attempts have mapped the end use demand of industry subsectors from a subset of contexts to all countries in Europe; for instance, Naegler et al.[21] use the German industry data in their analysis. Mantzos et al.[3] also undertake a top-down analysis to create the JRC-IDEES database, although it is unclear whether any country-specific end-use data was used to inform their model. Bottom-up modelling has used the FORECAST-Industry demand modelling tool[20,22,23], which demands a level of data input that is beyond the scope of our study. The HeatRoadmap Europe project[20] is an extension of Rehfeldt et al.[22], with the focus on a different reference year (2015 and 2012, respectively). There is no ground truth for industry end use demand, and datasets rarely align. Therefore we utilise the JRC-IDEES database, given that its structure best matches the data on total sectoral energy consumption published by Eurostat, as well as the use of 13 subsectors. JRC-IDEES provides sufficient data to understand electrified and electrifiable processes, as well as high and low temperature processes. We assume that steam processes still require methane and that diesel-powered backup generators are still in use for when grid access is disrupted**,** while all other processes can be electrified, according to the efficiencies provided in JRC-IDEES. A remaining issue is the consumption of fossil fuels as feedstock to industrial processes. Such consumption contributes to a large proportion of emissions in the chemicals industry[24] as well as for iron production[25]. To mitigate these emissions, new methods or feedstocks are required in the chemicals and steel industries, as will be detailed in the remainder of this subsection.

Iron and Steel

The process for producing steel requires two key steps: 1. iron ore to iron, and 2. iron to steel. In its current form, the first step is almost entirely conducted using *Blast Furnaces* (BFs) to produce "pig" iron (high carbon content iron) which cannot be fully decarbonised without CO_2 capture, due to the reliance on unreplaceable coke as the iron ore reductant[26,27]. A small (~6%) quantity of iron is produced as *Direct Reduced Iron* (DRI)[28], which relies on hydrogen (via natural gas) instead of carbon to reduce the iron ore. Although a proven process, the cost of reducing agent prohibits its large scale deployment[27].
The second step is currently dominated by the use of a *Basic Oxygen Furnace* (BOF), which reduces the carbon content of pig iron, to produce steel. The BOF requires large amounts of heat, as well as a source of oxygen (usually air), and is hard-linked to the BF as one unit, the *Blast/Basic Oxygen Furnace* (BF-BOF). This route releases CO_2 in the combustion of fuels, the production of lime and coke, and in the removal of oxygen from iron ore and excess carbon from pig iron, to produce steel[25].

The steel sector is highly circular, already around 85% of produced steel is recycled and approximately 40% of steel produced in Europe is from scrap[27]. Scrap steel enters the BF-BOF route, with blast furnace heat used to melt the scrap for addition into the basic oxygen furnace alongside iron, at about 10-20% of input ferrous material[25]. The remaining (majority of) scrap is processed using *Electric Arc Furnaces* (EAFs), which is an entirely electrifiable route of melting and recasting steel that accounted for 29% of all crude steel production in 2018[28].

Without carbon capture, the current BF-BOF cannot be fully decarbonised; only parts of the process could be replaced with biomass-based alternatives[25–27,29,30]. However, the direct reduced iron route could be decarbonised by direct use of hydrogen, instead of extracting it from natural gas. This process, known as H-DRI, could produce iron with low to no emissions. Following this, the electric arc furnace could be used as the primary method to produce steel; scrap steel and iron would be combined with a splash of carbon from coal to produce crude steel[27]. A study as part of the Hybrit project found that the H-DRI-EAF route would emit approximately 97% less CO_2 than the BF-BOF route, for the same crude steel production[31]. A less well developed, but potentially more energy efficient route for iron production involves direct electrolysis of iron ore (electrowinning). This technology is still at the laboratory phase[32], so little is known about its energy consumption at an industrial scale[b]. Indeed, it is explicitly not considered in the scenarios presented by the Material Economics consortium[27].

In the Sector-Coupled Euro-Calliope, we consider H-DRI-EAF as the primary route for future, decarbonised steel production, with an expected increased use of recycled steel overall to 50% of total ferrous material input (see Figure S5). This is in line with the Material Economics "new processes" pathway. Unlike these pathways, but similar to Hybrit, we would not only consider biomass sources of hydrogen, but also (and primarily) production by electrolysis from excess renewable generation. The hydrogen requirement for H-DRI is approximately 51kg per tonne of steel output[31], not accounting for the use of scrap steel in the EAF. That is, if 50% of the ferrous input in the EAF is scrap steel, and 50% is iron from H-DRI, then 25kg of Hydrogen would be required. In addition, electricity is needed for both the H-DRI and EAF processes. The Hybrit pre-feasibility

---

[b] *Fischedick et al.[32] use 9.3GJ/t crude steel in their calculations.*

study gives 2,633 kWh electricity for Hydrogen production, 322kWh for H-DRI, and 494kWh for EAF (+380kWh biomass and 42kWh coal), all per tonne of crude steel. Vogl et al.[31] agree on the electrolyser electricity consumption, but give 753kWh$_e$/t for EAF, approximately 250kWh/t for heating of iron ore, and <50kWh/t for H-DRI. Both ultimately give approximately 3,450kWh$_e$/t for the whole process, if no scrap steel is used, which is similar to the 3,640kWh$_e$/t given by Fischedick et al.[32]. On top of this, iron ore pelletising/sintering and downstream steel casting/rolling require 833 and 28/805 kWh, respectively[33].

Assuming 50% scrap and the production of one tonne of liquid steel, consumption of energy becomes: 25kg H_2, 135kWh$_{th}$ for iron ore heating and use in the H-DRI process, 710kWh$_e$/t for EAF, and 111kWh$_e$+625kWh$_{th}$ for pelletising, sintering and continuous casting[c]. Additionally, the "sponge" iron (output from H-DRI) can be allowed to cool and stored, effectively providing a buffer between energy production for H-DRI and for EAF; however, 159kWh/t is then required to reheat the iron for use in EAF. We ignore the requirement for coal and lime in the steel-making process but include energy demand for product 'finishing', as given by JRC-IDEES[3], which comes in at around 60-70kWh$_{th}$/t.

Quantity of steel production

For each country, we require the quantity of produced steel to understand future energy demand. Very few countries publish this data as part of Eurostat's PRODCOM database, so we instead use data on annual production for a select number of countries[28] to verify that energy consumption for iron and steel correlates with annual production, then map that to all Euro-Calliope countries.

All European countries (not including Turkey, Ukraine, Russia, and other CIS members) produced 172.8Mt of crude steel in 2018, 100Mt of which was produced by the BF-BOF route and the remaining 72.8Mt by EAF. BF-BOF derived steel is well matched to blast furnace energy consumption given by Eurostat (99.3% Pearson correlation; Figure S6a). All steel industry energy consumption is marginally less well matched to all crude steel production (97% Pearson correlation; Figure S6b), but still offers a useful avenue to disaggregate European steel production. In 2050, it is predicted that steel production will have increased across Europe to 199Mt[d]. We disaggregate this value to countries using total iron and steel subsector energy consumption.

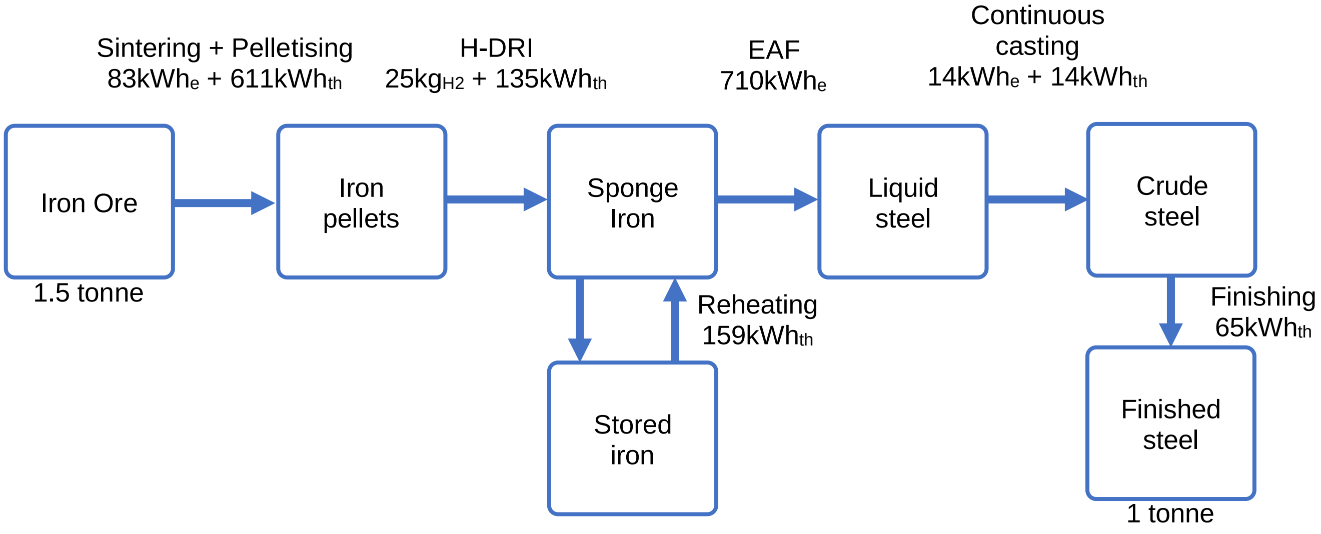

Figure S5: Iron -> Steel processes and the energy requirements to produce one tonne of cast steel.

[c] *We take the lower bound energy consumption for casting/rolling (i.e. for casting) since continuously cast steel makes up 97% of total crude steel production in the EU[28].*

[d] *193Mt from EU [27] + 6Mt from rest of Europe, using the same methodology of a 15% increase in steel production from 2016 to 2040.*

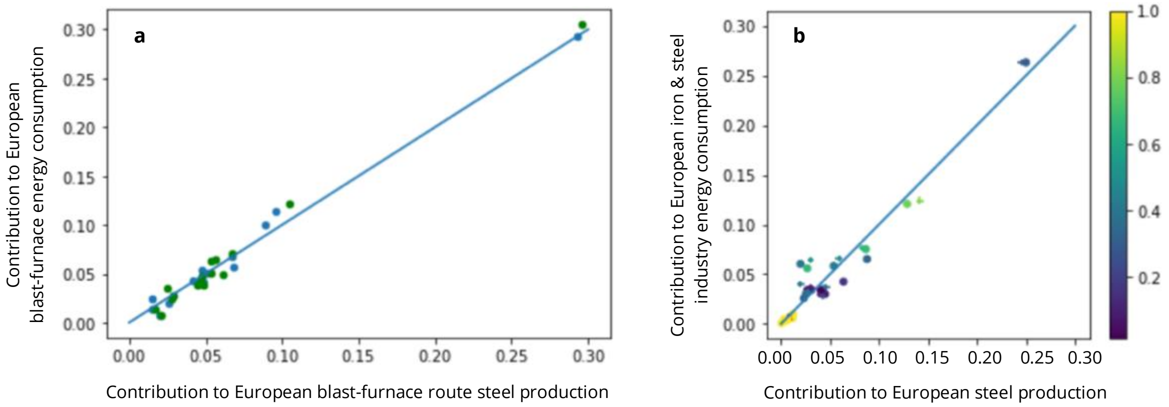

Figure S6: (a) Contribution of each European nation to total European BF-BOF steel production compared to national contribution to European blast furnace energy consumption. Blue = 2015, Green = 2018. (b) Contribution of each European nation to total European steel production compared to national contribution to European iron and steel industry energy consumption. Circles = 2015, crosses = 2018; colormap gives relative contribution of EAF to total steel production in each country. In both subplots, line shows correlation=1.

Chemicals

The chemical and petrochemical industry covers the production of end-use plastics, fertilizers, pharmaceuticals, and many other chemicals (glues, cleaning fluid, etc.). Of these, it is petrochemicals that require fossil feedstock; ammonia (for fertilisers), high value chemicals (HVCs, for plastic production), and methanol (for plastics and other chemicals) account for 90% of fossil feedstock in the chemical industry[34].

Plastics

There is a wide variety of plastics, almost all of which originate from the "cracking" of naphtha or liquified petroleum gas (LPG) into "high value chemicals" (HVCs) such as ethylene, propylene and BTX (benzene, toluene and mixed xylenes). The route to generating HVC without fossil fuel cracking is via methanol[35], which can be synthesised by hydrogenation of CO_2 or gasification/pyrolysis of biomass or waste plastics. Methanol synthesis is essentially the same as production of synthetic natural gas or synthetic liquid fuels, just with different compositions of the input gases. The energy requirements of these routes are given in Bazzanella and Ausfelder[35] and are (sometimes loosely) used by Material Economics[27] in its pathway generation. The "new processes" pathway assumes that chemical plastic recycling (40%) and biomass-to-plastics (33%) will dominate plastic generation in 2050, along with mechanical recycling (13%) and improved circular economies (14%). Figure S7 gives the process chains to realise final plastics production, with the inclusion of the option of hydrogen to plastics[35]. Energy requirements in some processes are ignored in Material Economics[27], and indeed there is little information available on some processes, including chemical recycling. Nevertheless, it is understood that external energy sources will be required to maintain e.g. the 900C required in a gasifier[36]. An additional route, mechanical recycling, could require 7MWh/t HVC[35], but this magnitude is too high relative to the available data on chemical recycling. Indeed, from various Swedish sources, Liljenström and Finnveden[37] found mechanical recycling to have an average energy consumption of 0.37MWh/t HVC.

According to the Material Economics "new processes" pathway, 28.8Mt of plastics in Europe will be generated by chemical recycling, 23.8Mt by new production (biomass or hydrogen), and 9.36Mt by mechanical recycling; an additional 10.1Mt would be circulated internally, so we do not consider them as energy consuming. This contrasts with the 64Mt of production in 2016 (60Mt by conventional means) given by Plastics Europe[38], and 47Mt of HVCs currently produced in Europe, according to the International Energy Agency[34]. The discrepancy between HVCs and plastics probably stems from imports of HVCs as well as production from other base chemicals.

Some countries have a greater discrepancy than others in their production of HVCs and subsequent production of end-use plastics. The Netherlands consumes 4.3% of Europe's converter plastics (penultimate step before reaching end-use plastics)[38] but produces 18% of EU-28's ethylene+propylene (see PRODCOM database), while Germany both produces 24.6% of those two chemicals and consumes 24.6% of Europe's converter plastics. Since we are concerned with replacing fossil feedstocks, we will focus on the generation of HVCs, and therefore use non-energy consumption of Naphtha from the Eurostat annual energy balances to infer national contributions to European production of HVCs. Figure S8 shows that this is a reasonable assumption, with Naphtha consumption more often matching PRODCOM data compared to plastics production data. This

comparison comes with the caveat that many countries have no PRODCOM data, and BTX HVCs are not included (again, for lack of PRODCOM data).

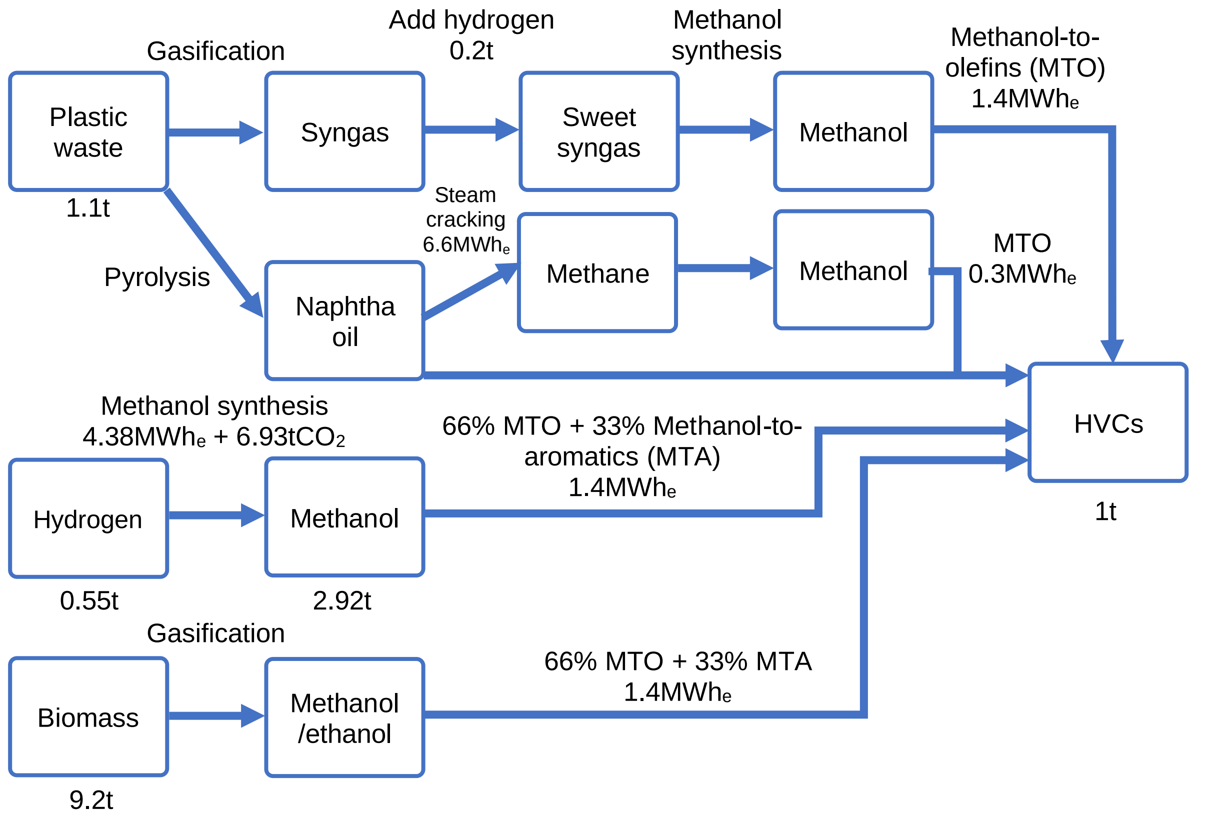

Figure S7: Possible routes to zero/low carbon plastic production, based on a combination of Material Economics[27] and Bazzanella & Ausfelder[35]. There are some data gaps with no reliable source, including energy demand for plastic gasification and pyrolysis. Biomass gasification energy use is incorporated into its overall efficieny (hence the high input requirements).

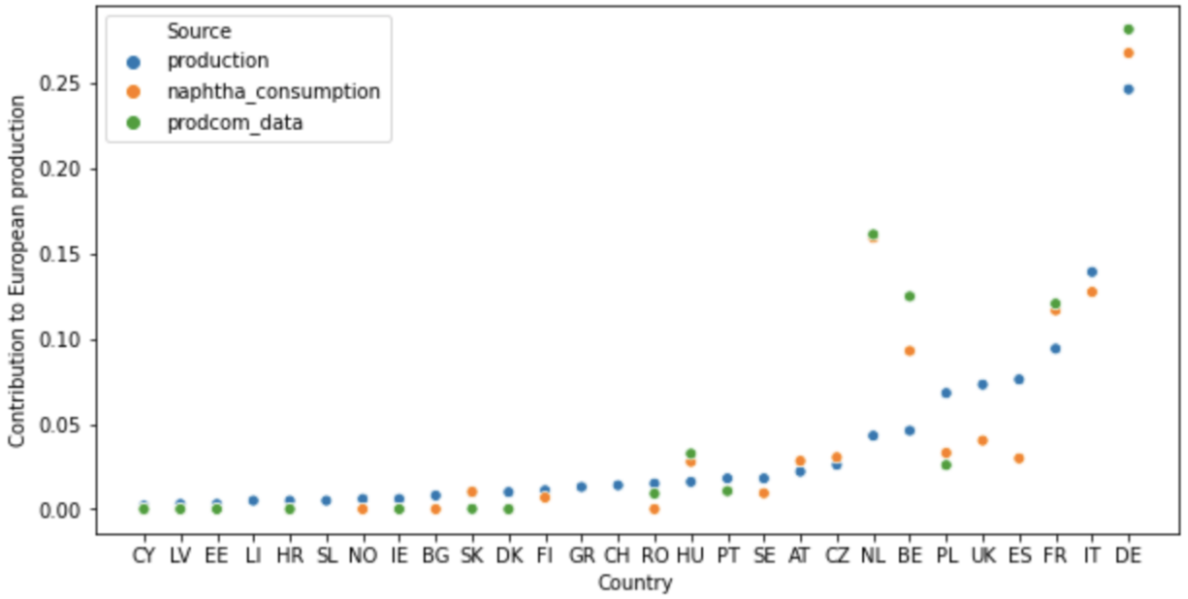

Figure S8: Comparison of data sources to infer the contribution of countries to European HVC production in 2018. 'production' is the contribution to production of end-use plastics according to Plastics Europe[38], Naphtha consumption is from Eurostat energy balances, and 'prodcom_data' relates to published data on produced volume of Ethylene and Propylene, where available.

Ammonia and Methanol

Produced from steam reformation of natural gas or gasification of coal, ammonia and methanol rely on a source of hydrogen for their production. Ammonia is produced by combination of hydrogen and nitrogen, while methanol is produced by the combination of hydrogen and CO_2. Of the replacement fossil feedstocks, this is the most straightforward: Ammonia requires $0.178t_{H2}/t$ + $1.73MWh_e/t$ for compression and N_2 production; Methanol requires (as seen in the previous subsection) $0.189t_{H2}/t$ + $1.5MWh_e/t$ + $1.373t_{CO2}/t$[35]. An additional component of this process chain is urea, which currently relies on the steam and CO_2 output of Ammonia production, adding 0.92MWh/t + $0.32_{tCO2}/t$ urea. Table S5 shows the annual production of Ammonia, which ranges from 15 to 28Mt in a year. Given the collection of values in the range 15-19 Mt, we take 17Mt as the annual production, as given by Bazzanella and Ausfelder[35]. Their number is based on a dataset from Fertilizers Europe which is no longer available. We also take the urea production from the same source: 6Mt, leaving 13.6Mt for direct ammonia and 3.4Mt for ammonia-> urea[e].

Table S5: Annual ammonia production in Europe (EU or all Euro-Calliope regions) according to various sources.

Source	Year	Quantity
International energy agency[34f]	N/A	27.5Mt ammonia
PRODCOM	2017	16 Mt ammonia (13.1 Mt N)
USGS[g]	2017	18 Mt ammonia (14.6 Mt N)
Boulamanti and Moya[39]	2013	19 Mt ammonia
Fertilizers Europe	2017	15 Mt ammonia (12.4 Mt N)
Bazzanella and Ausfelder[35f]	2016?	17 Mt ammonia

Bazzanella and Ausfelder[35] depend on PRODCOM for methanol production data, which was 1.6Mt in 2018. This compares to 4.5 Mt given by the International energy agency[34]. Global demand for Methanol was 75 Mt in 2015, 3% of which was produced in the EU[34], giving 2.6 Mt. Based on these fluctuating values, we take a value of 2 Mt.

Molar contributions

To match with JRC IDEES basic chemicals (given in weight of ethylene), we take their values of production and attribute them by molar ratio to plastics, ammonia, and methanol. Results of this are shown in Table S6. Applying this to the JRC IDEES dataset leads to an overprediction of chemical production (by about 10-15%); this is expected, since we do not cover all basic chemicals in this analysis.

Table S6: Comparison of basic petrochemicals, including their approximate annual production in Europe, molar mass, and molar contribution to total moles of basic chemicals produced.

Chemical	Annual production (Mt)	Molar mass	% molar share of chemicals
Ethylene	21.7	28.05	32.1
Propylene	17.0	42.08	16.8
BTX	15.7	93.00[h]	7.01
Ammonia	17.0	17.01	41.5
Methanol	2.00	32.04	2.59

In the Sector-Coupled Euro-Calliope, we consider chemical production as requiring a combination of three molecules: CO_2, H_2, and methanol. Together, these can be used to produce the basic chemicals for plastics, as well as ammonia and urea. We estimate the quantity of each basic chemical produced in Europe, and assume that each country produces the same relative share of those chemicals. With this assumption, we can disaggregate the JRC IDEES estimate of national production of basic chemicals (in kt ethylene) to the chemicals of interest, and from there calculate the demand for CO_2, H_2, and methanol based on data of each transformation technology[35]. This gives the annual demand for CO_2 (kt), H_2 (MWh LHV), and methanol (MWh LHV) for the chemical industry of each country.

[e] 0.57 tonnes ammonia is needed per tonne of urea[35].

[f] Base source is Fertilizers Europe, but data nitrate subsets is only available to paying members.

[g] Only covers 22 of the Euro-Calliope countries (probably the biggest producers). Alternatively, taking total global production of 144Mt multiplied by the 9 and 12% contribution of the EU, according to fertilizers Europe and the IEA, respectively, we get 12.9-17.3Mt.

[h] Average of the components of BTX, which range from 78 to 106 g/mol.

Other industry subsectors

Feedstocks are not such a concern in other industry subsectors, so we use JRC IDEES data directly. The only changes we envision in these subsectors is the electrification of machinery and medium temperature processes. The only subsector which produces notable emissions through processes that cannot be decarbonised is the cement industry. There is speculation about the possible avenues for processes and materials that could be used to mitigate cement industry emissions[24,27], but these are rarely energy-based solutions. Therefore, we ignore non-energy emissions from the cement industry.

OTHER SECTORS

The final sectors not covered by any of the previous subsections are agriculture & forestry, fishing, and "not elsewhere specified". These sectors account for approximately 2.5% of total European annual energy demand. According to Eurostat, "not elsewhere specified" demand is attributed to the military, among other things. These sectors are not handled completely by JRC IDEES, so we take a different approach. We assume all oil consumption to be for mobility and added to annual demand for heavy-duty vehicles (*agriculture & forestry* and non-kerosene use in *not elsewhere specified*), shipping (*fishing*), and aviation (kerosene in *not elsewhere specified*). We assume all other non-electricity consumption to be for heating applications, and therefore is added to annual commercial heat consumption.

SYNTHETIC FUEL PRODUCTION

All subsectors have the option, or indeed the requirement, to meet demand with net-zero emission hydrocarbons (henceforth 'synthetic fuels'). Kerosene, diesel (used also as a proxy for petrol), methanol, and methane are energy carriers in the Sector-Coupled Euro-Calliope. All of these fuels can be generated from electricity or biofuels. If generated from electricity, then hydrogen and CO_2 are first produced from electrolysis and direct air capture, respectively, before being combined in various processes to produce the hydrocarbons. We have collated these processes and associated technologies almost exclusively from the Danish energy agency technology database[12], with the exception of direct air capture of CO_2 (from Fasihi et al.[40]) and electrolysis data (following the same approach taken in Lombardi et al.[41]).

EURO-CALLIOPE REGIONALISATION

To represent the geographic disparity across all energy-consuming sectors, a sub-national spatial resolution is necessary. However, two issues arise when moving to a subnational level: (1) administrative regions are often not at the correct resolution or are at significantly different resolutions between two countries[i], and (2) the transmission system is not well understood by modellers below the national level. Between countries, Net Transfer Capacities (NTCs) are available from European Network of Transmission System Operators for Electricity (ENTSOE). At a sub-national level, this is rarely the case. To address both these issues we adopt a regionalisation first developed within the European Commission Seventh Framework Programme project e-HIGHWAY 2050. Within this project, a pan-European transmission system representation was developed based on grouping NUTS3 administrative regions to 106 model regions[42]. Grid transfer capacities between model regions were calculated in e-HIGHWAY 2050 based on a detailed, proprietary understanding of the transmission system. By assuming the same model regions, we are able to ensure a high resolution as well as a detailed understanding of transfer capacities of the transmission system in the Sector-Coupled Euro-Calliope. Figure S9 shows the final 98 model regions[j], including all possible inter-region high-voltage transmission connections. These connections include those already existing in e-HIGHWAY 2050 as well as planned connections in the medium and long term, predominantly high voltage DC (HVDC), according to ENTSOE 2018 network development plan (TYNDP). In the model, we use the capacities of these existing/planned transmission lines as a lower limit, then allow lines to increase at a cost. Upper limits on lines are set to allow initially low capacities to increase more than initially high capacities: up to 40x (<1GW), up to 10x (1-5GW), up to 5x (5-10GW), up to 3x (10-15GW), up to 2x (>15GW). These upper limits were chosen after testing different limits, to enable possible renewable generation hubs (e.g. northern Scandinavia) to increase transmission capacity sufficiently, whilst not allowing excessive capacity increases in already congested regions. The overall upper bound on system-wide capacity increase is 8.9x. We use known costs or expected costs of TYNDP planned connections to estimate transmission expansion costs in Euro-Calliope, grouping transmission lines into five types based on technology and geographic context (Figure S10). Each line type cost spans a relatively large range; we take the median cost for the baseline Euro-Calliope model in each instance.

---

[i] *For instance, Germany has 401 NUTS3 regions, while France has 101; France has almost twice the land area, and 80% the population of Germany, but has ¼ the number of NUTS3 regions.*

[j] *There are 98 model regions in Euro-Calliope compared to 106 in the original e-HIGHWAY 2050 model due to not including neighbouring countries (incl. Russia, Belarus, Ukraine, & North African countries).*

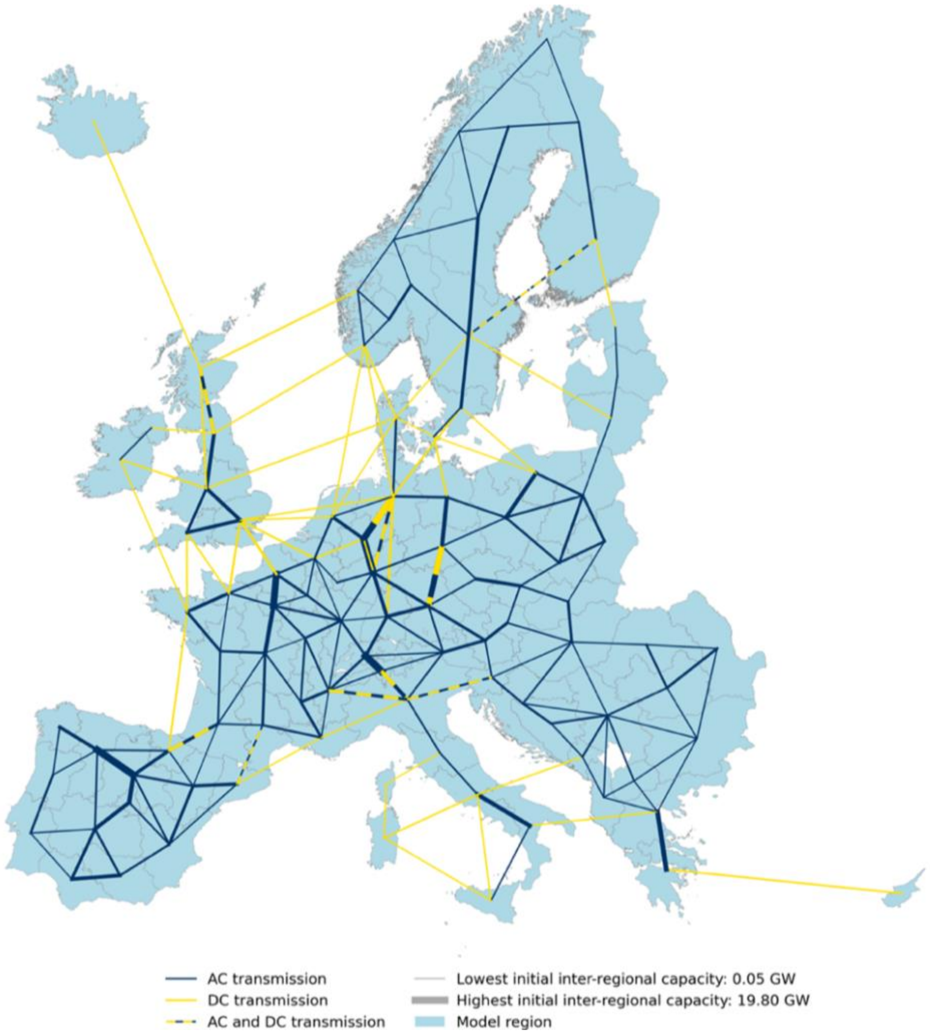

Figure S9: Sector-coupled Euro-Calliope 98 regions and inter-regional transmission lines. Lines are coloured based on the type of transmission available between regions. Thicker transmission lines represent larger modelled initial grid transfer capacities. All lines can expand beyond their initial capacities.

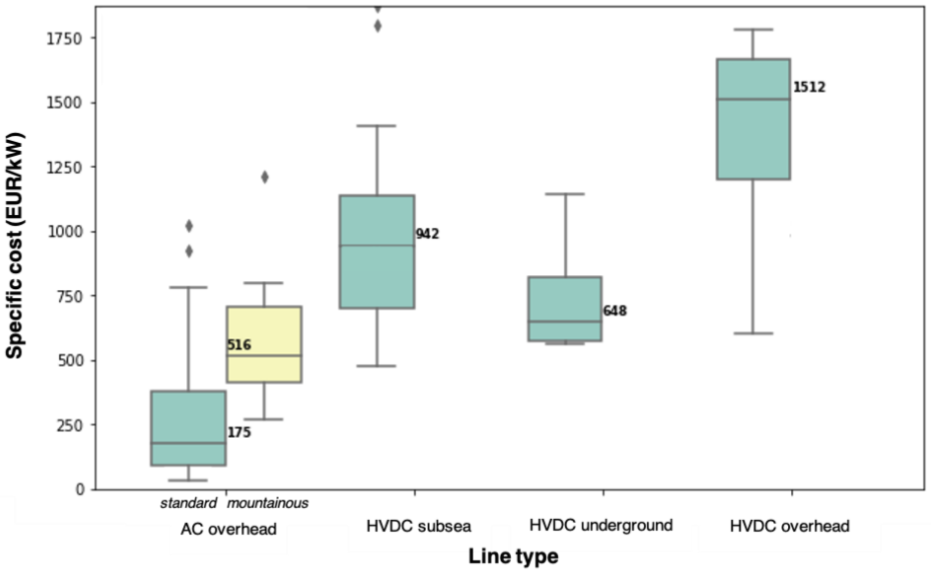

Figure S10: Distributions of transmission costs for five line types. AC = Alternating current, HVDC = high voltage direct current. AC lines are split into those which span mountainous regions and those that do not. Distributions are based on costs of implementing recent line extension projects as well as budgets for planned extensions; there are 92 data points in total.

To regionalise sub-sectors, we use different datasets for different end-uses. In each case, we compared several sub-national indicators to samples of published regional data, to test their viability. We regionalise **household and public and private passenger transport demand using populatio**[k]; **commercial building and light-duty vehicle demand using NUTS3 Gross Value Added** (GVA) from non-industrial subsectors (classifications *G-U*); and **industry demand, including from freight transport, is regionalised depending on subsector**. For industries with emitters registered in the EU-ETS, we use the location and size of emitters in 2014 for regionalisation (Figure S12). We found that these largest emitters capture most subsector emitters in each country, when compared to Eurostat annual emissions balances within each subsector (Figure S11). For all other subsectors, we found the number of employed individuals in each industry sub-sector (NUTS2) to be the best indicator, but it did not provide sufficient resolution for full disaggregation. We achieve NUTS3 regionalisation by combining number of employees with the quantity of loaded freight in each industry subsector. We regionalise **demand for aviation and shipping fuels based on average industry regionalisation**, on the assumption that these fuels would be synthetically generated in industrial regions, rather than exclusively at the point of consumption (e.g. major ports for shipping fuel).

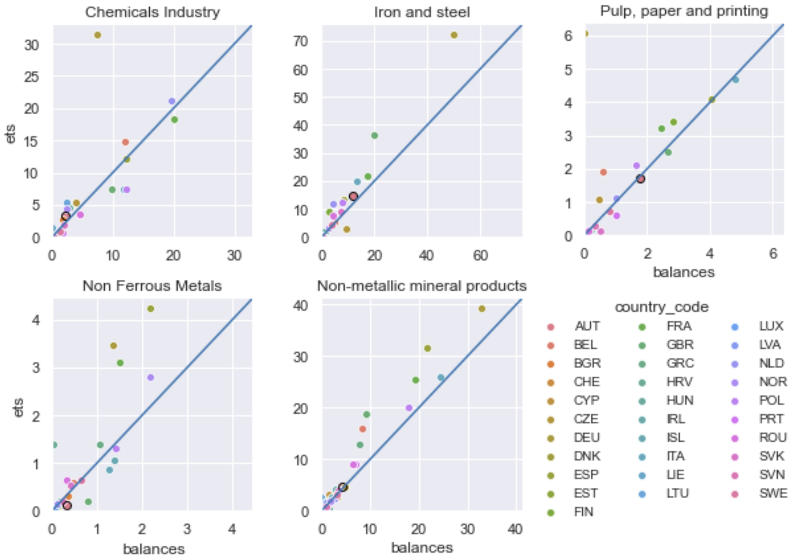

Figure S11: Published industry subsector CO_2 emissions ("balances") compared to the sum of reported emissions from industry sites within each subsector, via the EU-ETS ("ets"). Extreme outliers are caused by reporting inconsistencies; e.g. Chemicals industry emissions in Germany are not reported in Eurostat under the subsector emissions, but instead grouped into 'Other industry emissions'.

[k] *In the UK context, we found that subnational gas and electricity demand [43] was best correlated with population. Other indicators we tested include built environment land-use, GDP, and heating degree days.*

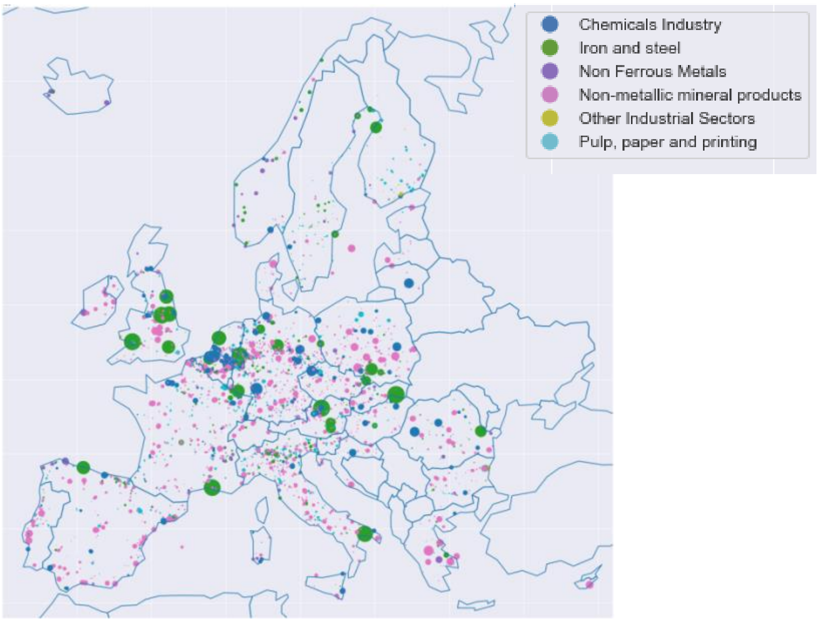

Figure S12: Spatial distribution of all sites which report to the EU-ETS / ERPTR and whose data has been collected in the Hotmaps database (3411 sites) as well as a further scraping of the EU-ETS database (722 additional sites). Marker colour depicts subsector classification, while the marker size relates to quantity of actual emissions reported for the year 2014.

POWER SECTOR UPDATES

Although our primary aim in updating Euro-Calliope was to add all non-electricity energy sectors to the existing model, we also made some updates to the v1.0 power system model. As well as the aforementioned updates to the transmission system representation, we have included 2050 nuclear power and combined cycle gas turbine (CCGT) technologies, current underground gas storage capacity from GIE, and updates to hydropower capacity. We base nuclear capacity in 2050 (Table S7) on published data of expected capacity in select countries, regionally distributed by current capacity from the JRC open power plants database[4]. CCGTs are expected to consume synthetic methane and they have no constraints on total capacity. We take the 2040 projection from the UK Department for Business, Energy & Industrial Strategy report on electricity generation costs to estimate the 2050 cost of CCGTs. Euro-Calliope v1.0 already relies on Hydropower capacity from the JRC Hydro-power database, which we update to version 7[44]. We also remove a scaling step, such that JRC data on pumped storage is used directly, rather than being scaled to fit capacities assumed by Geth et al.[45].

Table S7: Range of installed nuclear capacity in 2050 for subset of European countries in which some nuclear capacity is planned or under consideration. Countries given by their ISO3 codes.

		BGR	CZE	FIN	FRA	GBR	HUN	ROU	SVK
**Installed capacity (MW)**	*Min*	0	6230	0	22000	8900	2400	650	940
	Max	3200	7860	2750	58600	8900	2400	2650	3340

FILLING DATA GAPS

During data processing there are many cases where there are gaps for certain countries, years, end-uses, or energy carriers. Specific data filling can be found in the data processing workflow. The primary data filling methods we utilized are:

1. If not available in Eurostat or JRC IDEES, namely Switzerland, nationally published data has been used.
2. Where possible, gaps are filled using total sectoral energy demand. In years with data, an average contribution of each end use to total demand is calculated (e.g. X% of demand is for cooking); this average contribution is then applied to gaps (e.g. cooking demand in year Y = household demand in year Y * X%).
3. If end-use data is unavailable (e.g., for commercial and industrial heat demand), gaps are filled in at the energy consumption stage. The average relative contribution of each energy carrier to each end use is applied to all years without JRC-IDEES data but with Eurostat annual energy balance data.
4. If no data is available from the Eurostat annual energy balances, we take demand to be the average demand for that end-use for all years that we have data.
5. If no data is available at all for a country (e.g., we do not have cooking profiles for some eastern European countries), the average of data from the closest available neighbouring countries is used.

FINAL SECTOR-COUPLED ENERGY SYSTEM MODEL

In each of the aforementioned sectors, there exists some degree of electricity demand in today's energy system. The extent of this demand is quite limited in some sectors, e.g., 1.7 TWh in 2018 passenger road transport, but can be considerable in others, e.g., 670 TWh in 2018 building heat demand. To avoid double-counting demand, we remove existing heat and vehicle electricity end-use consumption from the electricity load curve.

Finally, although all abovementioned subsectors and energy carriers can be modelled in the Sector-Coupled Euro-Calliope, we have made some simplifications in the context of this study to ensure model tractability:

1. Road vehicles are grouped into *heavy* (heavy-duty vehicles and busses) and *light* (light-duty vehicles, motorcycles, and passenger cars), with electric vehicle plug-in profiles only applied to *light* vehicles.
2. Air-source and ground-source heat pumps are represented by a single technology, whose characteristics are a weighted average of the two main heat pump classes. The weighting is based on the ratio of air- to ground-source heat pump sales in 2016 and 2018, according to the European heat pump association.
3. The number of combined heat and power technologies has been reduced from six to three, by selecting only those technologies that are likely to be more prevalent in future according to expert opinion.
4. Industry feedstock demand for hydrogen and CO_2 can only be met by electrification in the model (electrolysis and direct air capture, respectively). We have therefore added this to industry electricity demand directly.

The final flow of energy carriers from supply to demand defined in Euro-Calliope is depicted in Figure S13.

Updates to the Calliope modelling framework

To represent all new sectors in Euro-Calliope, we have added mathematical constraints on top of the underlying Calliope modelling framework. New constraints include:

- Time varying technology capacity applied to heat pump heat capacity and electric vehicle charging capacity.
- Annual average capacity factor upper and lower bounds applied to nuclear technologies.
- Combined heat and power operating ranges, to capitalise on characteristics provided by the Danish Energy Agency technology database (C_b and C_v coefficients).
- Fixed demand share as a decision variable, applied to the share of heat and transport supply technologies. With this constraint we are able to model the operation of disaggregated technologies, e.g., household heat supply. For instance, if 60% of households invest in heat pumps then only approximately 60% of heat demand can be met by heat pumps in each hour.
- Demands can be time independent. This allows e.g., kerosene for aviation to be generated according to any profile, provided there is sufficient kerosene generated by the end of the model period.

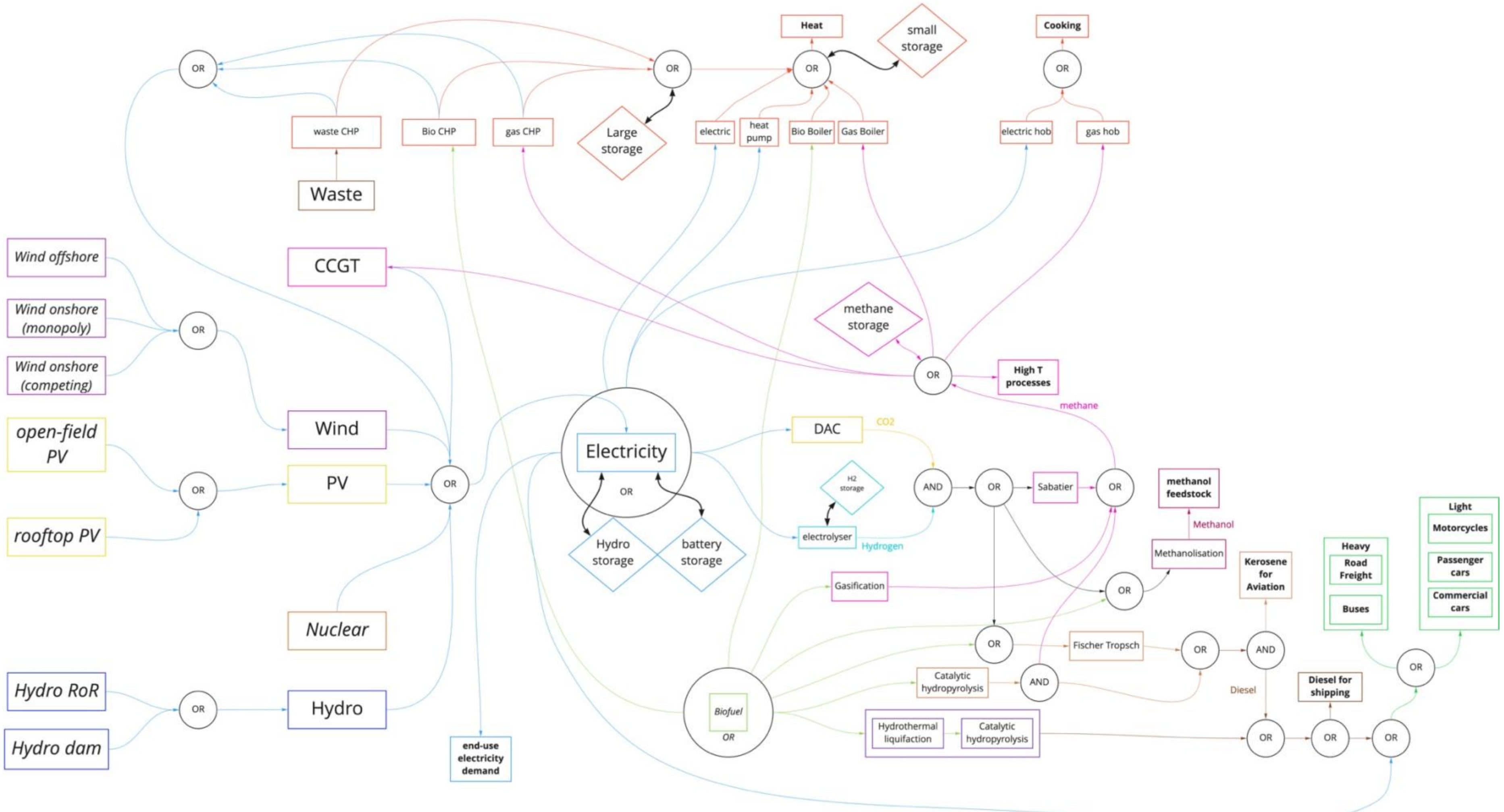

Figure S13: Flow chart of the final Euro-Calliope sector-coupled model, including supply technologies, energy carriers, and demand sources. AND and OR icons in the flow indicate when an in/output is a combination (AND) or choice (OR) of out/input. Where energy enters the systems from an external resource, the carrier is given in italics (e.g. Waste). Storage technologies are depicted as diamonds. Bold text refers to demands, where energy exits the system. Flow line colours are related to the energy carrier flowing along that line.

SUPPLEMENTAL ITEMS

NOTE S2: SENSITIVITY ANALYSES

The aim of the sensitivity analysis (SA) that we describe in this section is to assess how sensitive our conclusions are to input parameters that we deem most likely to change in reality. To do this we use the one-at-a-time (OAT) approach, which is commonly used in computationally intensive models that do not lend themselves to a global SA[46]. Unlike traditional energy modelling efforts which also undertake OAT SA (e.g. [47–49]), our conclusions are concerned with the diversity of options and the trade-offs between those options, and not with specific quantities of individual technologies or costs of a small set of solutions. Therefore, our SA focusses on assessing whether changes in input parameters produce significant deviations from our conclusions that the option space and trade-offs are general features of the European energy system.
Because of its spatial and temporal detail, the sector-coupled Euro-Calliope model is computationally intensive (12 to 20 hours needed to compute one SPORE, with limited scope for parallelisation). Therefore, we undertake an SA which balances the need to critically reflect on the modelling process with the computationally intense nature of our work. We limit ourselves to only three input parameters/parameter groups: weather year, annual demand projections, and cost relaxation. The setup and results of these analyses is described in the following sections.

WEATHER YEAR

The model is run for the baseline year of 2018, which we use to derive service demands and variable renewable technology resource availability. In our previous work, we have shown that the choice of weather year has a greater impact on the option space of SPORES than other key uncertain parameters (demand and technology costs)[41]. However, it is impractical to run SPORES for all weather years, so we focus on the structure of the cost-optimal solutions, to see whether there are any spatio-temporal dynamics of supply and demand in the years 2010 - 2017 that are so different to 2018 as to suggest the need for a drastically different energy system configuration. That is, we check to ensure that there is no overfitting in the context of our conclusions. We do not find evidence for this.

The result of this comparison is shown in Figure S14 and Figure S15. As can be seen, the cost optimal results are within the bounds of the SPORE results for the weather year 2018, whether for absolute capacities or our nine metrics of interest. There is often very little spread in capacity between the years, particularly for the highest capacity technologies (AC transmission, PV, wind, electrolysis). CCGT and battery storage capacity does vary more noticeably between weather years, suggesting that weather variability is more pronounced in 2015 and 2016, requiring additional flexibility. Across our metrics, differences are also generally very small, with biofuel utilisation varying most prominently. In addition, the system cost of the different systems varies by a maximum of 3.5% compared to the 2018 optimal system cost; i.e., significantly less than the cost relaxation. Since these variations between weather years are noticeably lower than the range of SPORES, with no weather year metric or capacity sitting outside the range of SPORES, we do not deem there to be significant weather year overfitting.

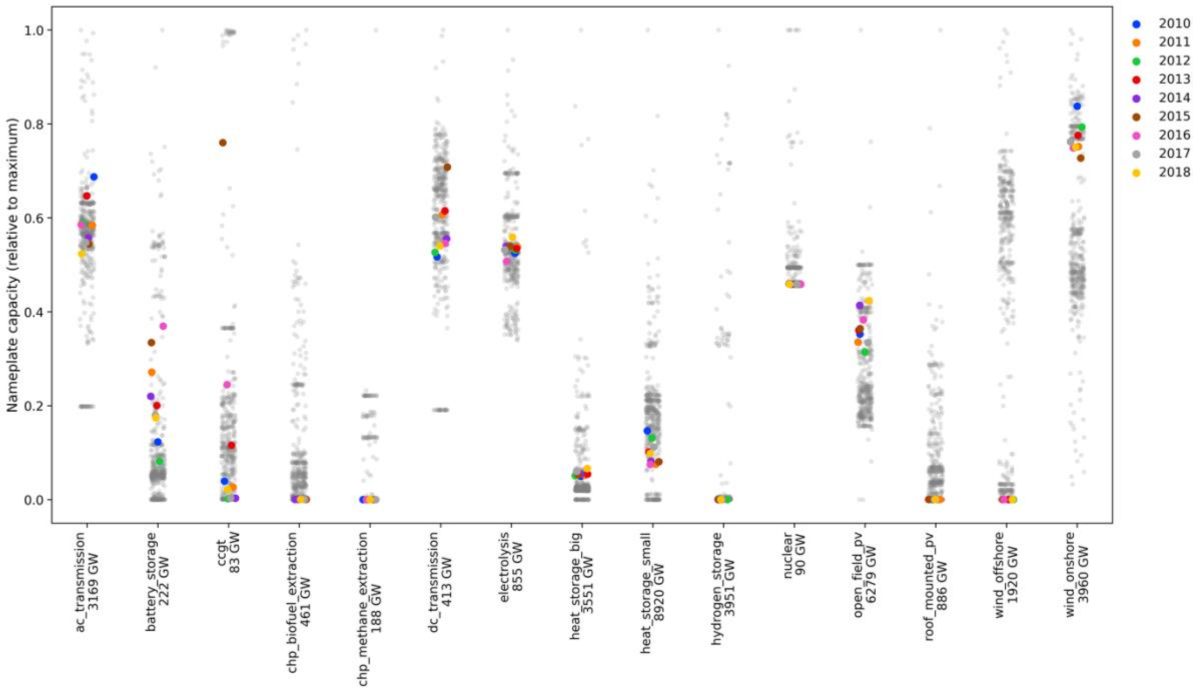

Figure S14: Technology nameplate capacities in all 10% relaxation SPORES (441 results; light grey markers) and in all cost-optimal results across the weather years 2010 – 2018 (coloured markers). SPORES are based on the 2018 weather year. Technologies include those that produce, transmit, or store energy. Storage capacities refer to the charge/discharge capacity. Results are shown scaled to the maximum capacity for each technology, across all results. The value of this maximum capacity is given next to the technology name on the x axis.

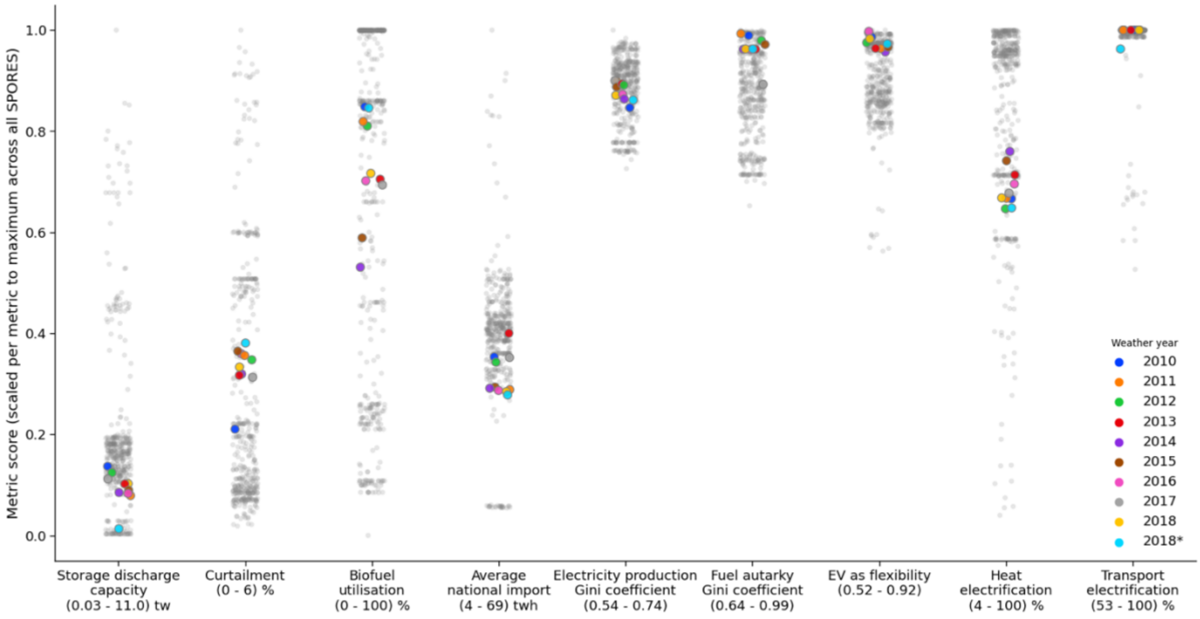

Figure S15: Scaled metric scores in all 10% relaxation SPORES (441 results; light grey markers) and in all cost-optimal results across the weather years 2010 – 2018 (coloured markers) and for the weather year 2018 with projected demands (2018). SPORES are based on the 2018 weather year. Absolute metric ranges are given in brackets underneath each metric name on the x-axis.*

DEMAND PROJECTIONS

In the base model, we use 2018 service demands derived from the pre-processing steps described in Note S1. We conduct this sensitivity analysis to check whether key features of the SPORES option space is affected significantly by our choice to use 2018 service demands directly.

We use simulated increases in national demands for end-use services from the models DESTINEE[17] and HEB[50], according to scenarios aimed at pan-European carbon-neutrality by 2050. We do not use the absolute 2050 demands directly, but scale Euro-Calliope service demands according to the simulated increases from 2015 to 2050. Scaled demands are described further in Table S8. We do not have data on demand scales for all 35 modelled countries; where no data is available, average scales from neighbouring countries have been used. Increases in demand are seen in industry and transport, due to the simulated effects of increases in population, average income, and value added. Building-level demands also include these effects, but they are outweighed by simulated reductions of demand intensity, due to appliance efficiency improvements and deep building retrofit. Final model demands after applying scaling factors are available in the data files published with this study: DOI:10.5281/zenodo.6546817.

We optimised the scaled demand model for the 2018 weather year, maintaining the shape of timeseries profiles from this weather year and only scaling the annual magnitudes of demand. We also produced 73 SPORES, focussing on producing system configurations with greatest technological differences, rather than spatial ones. As expected, the cost-optimal energy system differs when using scaled demands; the objective function value increases by 20%. Yet, the metric scores of this cost-optimal solution is very similar to that given by other cost optimal solutions (Figure S15 – 2018*). We also find that the option space exposed by our SPORES remains roughly the same, whether using 2018 end-use demands directly or modelling with projected demand increases. For our nine metrics defined in Table 1, Figure S16 shows the similarity in the extent of metric values between the two demand scenarios. Although SPORES are concentrated in different places between the two scenarios, the range they cover is similar. This means that irrespective of the demand scenario used, system designs exist that enable similar levels of maximisation/minimisation of particular metrics.

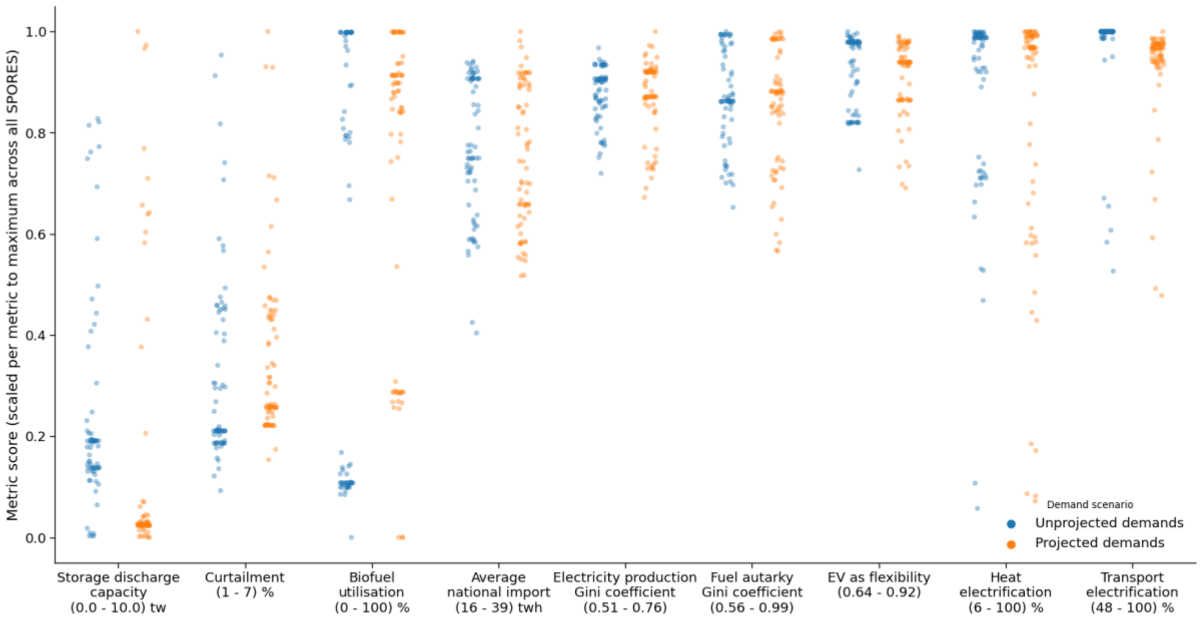

Figure S16: Impact of projected demands, summarized in Table S8, on the range of SPORES across the nine high-level metrics used to understand the option space in this study. Absolute metric ranges are given in brackets underneath each metric name on the x-axis.

Table S8: Summary of data sources to scale demands and the impact on modelled demands of including these scales.

Sector	Simulated data	Demand scenario source	Increase in total demand, relative to baseline 2018 model (%)
Industry	Sub-sector value added	DESTINEE	1.39
Road transport	Number of vehicles and total vehicle kms	DESTINEE	1.27
Aviation & shipping	Total vehicle kms	DESTINEE	1.62
Rail	Total vehicle kms	DESTINEE	1.59
Building appliances & cooling	Electricity consumption	DESTINEE	0.77
Building heat	Energy consumption	HEB	0.27

Cost relaxation

For the baseline year 2018, we also test a subset of SPORE runs with 5% and 15% relaxations. We generated 120 SPORES in total per sensitivity run. We compare the results for the equivalent runs in the baseline (10% relaxation) run. These SPORES focus on excluding specific technology groups whilst exploring spatial diversity of primary electricity supply. Here, we compare the range of high-level metrics across SPORES for the different relaxations, using the same metrics as shown in Figure 3 of the main text.

The result of this comparison is shown in Figure S17. As expected, the range of metric values increases with increasing cost relaxation. In some cases, a relaxation of 5% does not sit within the same range as achieved with a relaxation of 15%. For instance, the lowest average national import metric score is 0.48 for a relaxation of 5% and 0.53 for a relaxation of 15%. A relaxation of 15% could unlock metric scores which differ from our baseline 10% relaxation when they are already showing a significant difference in this subset of results; that is, transport electrification, biofuel utilisation, and EV as a flexibility source (through smart charging). If these metrics are of particular interest to minimise, a full set of SPORES at a 15% cost relaxation would need to be generated.

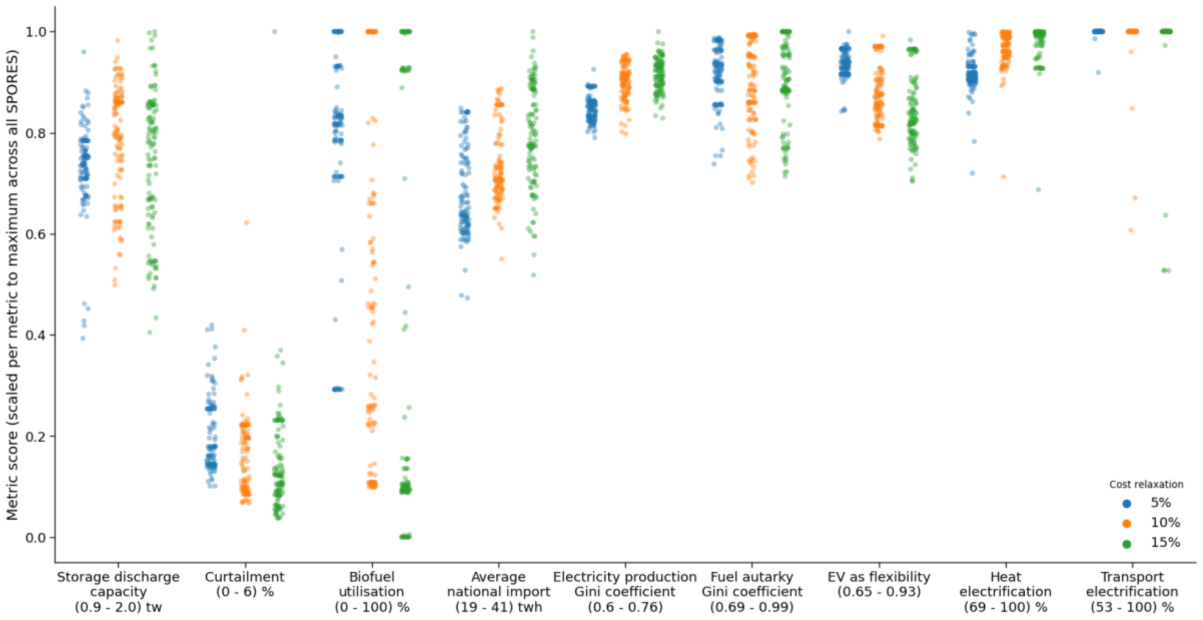

Figure S17: Comparison of high level metric scores, scaled to the maximum value across all SPORES. 120 SPORES are shown, including all those for which there is a result in all cost relaxations. A description of the metrics can be found in the main text (Table 1). Absolute metric ranges are given in brackets underneath each metric name on the x-axis.

NOTE S3: SEASONAL BALANCING MECHANISMS

By comparing monthly energy production across all 441 baseline SPORES, we aim to identify the key sub-seasonal to seasonal balancing mechanisms across SPORES. Figure S18 shows that PV and wind monthly variations in production are most strongly (anti)correlated with monthly variations of demands. Indeed, strong dependence on wind production goes a great length to balance monthly demand variations across seasons. Dispatchable energy supply (biofuels, waste, and CCGT) can either help balance seasonal demand variations (positive correlation) or balance other flows in the system (negative correlation). For biofuel and waste supply, this choice on how much to correlate/anticorrelate with demand exists irrespective of the dependence of the SPORE on wind or solar production. Conversely, CCGT operation is used more often to balance seasonally in SPORES with high PV dependence, and used to balance on other timescales in SPORES with high wind dependence. SPORES with high dependence on PV production also rely on hydrogen (and, subsequently, synthetic fuel) production and storage. This leads to the strongest anticorrelation of hydrogen production with end-use demands, as fuel is produced predominantly in the summer months. Hydropower dependence can change seasonally, by controlling the output of reservoirs and use of pumped storage. Again, the strongest seasonal (anti)correlations are at the extremes of wind/PV production: high PV production means high dependence on reservoirs to follow seasonal demands, but high dependence on pumped storage to balance PV supply; high wind production means low dependence on reservoirs to follow seasonal demands, but (slightly) positive correlation between demand and pumped hydro output. Methane cavern storage acts inversely to pumped hydro storage.

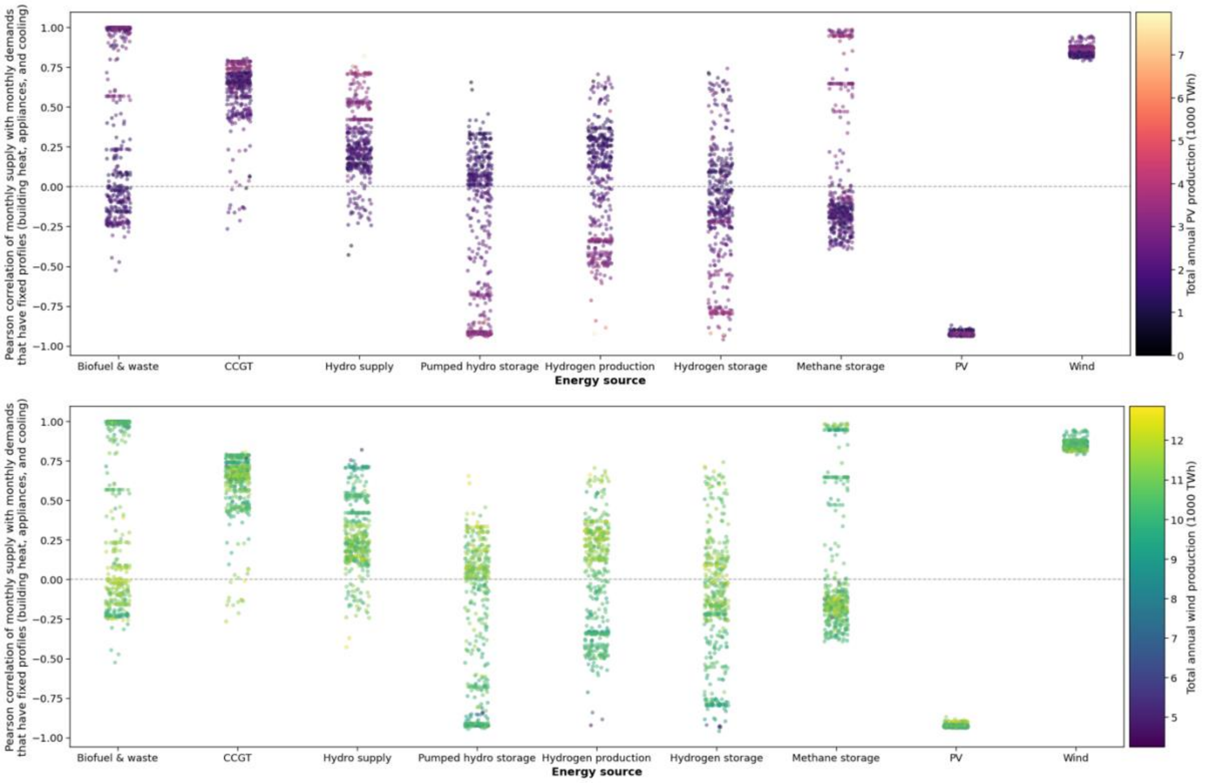

Figure S18: Pearson correlation of monthly total European production with demand across all 441 baseline SPORES, with each SPORE highlighted according to total annual European PV (top) and wind (bottom) electricity generation in that SPORE. A positive correlation indicates that a large fraction of the monthly variation in demand is matched by equal levels of monthly variation of supply. This means that matching on a monthly level without flexibility mechanisms is relatively easy. We have only selected for comparison those production technologies that can enable seasonal balancing: 1. Biofuel and municipal waste supplies, for use in direct combustion to meet end-use demands or to produce intermediate fuels; 2.combined cycle gas turbine (CCGT) electricity output; 3. reservoir and run-of-river hydropower electricity supply (Hydro supply); 4. Pumped hydro storage electricity output; 5. electricity consumed to produce hydrogen by electrolysis (Hydrogen production); 6. Hydrogen storage tank output; 7. Methane cavern storage output; 8. rooftop and open-field PV electricity production; 9. onshore and offshore wind electricity production. Only fixed timeseries demands are used in the correlation, which includes the base electricity demand (building appliances & cooling, rail, and industry processes) and building heat demands. Fuel and transport distance service demands are balanced on an annual basis, so are not included.

NOTE S4: LINKED SPORES METRIC VALUES

The following figures (S19 to S26) are the same as Figure 3a, but each highlights a different set of linked SPORES values. In the main text, linked SPORES are highlighted according to those which fall within the lowest 15 percentage points (+15pp) of the biofuel utilisation metric. In these additional figures, the linked +15pp SPORES of the remaining eight metrics are shown.

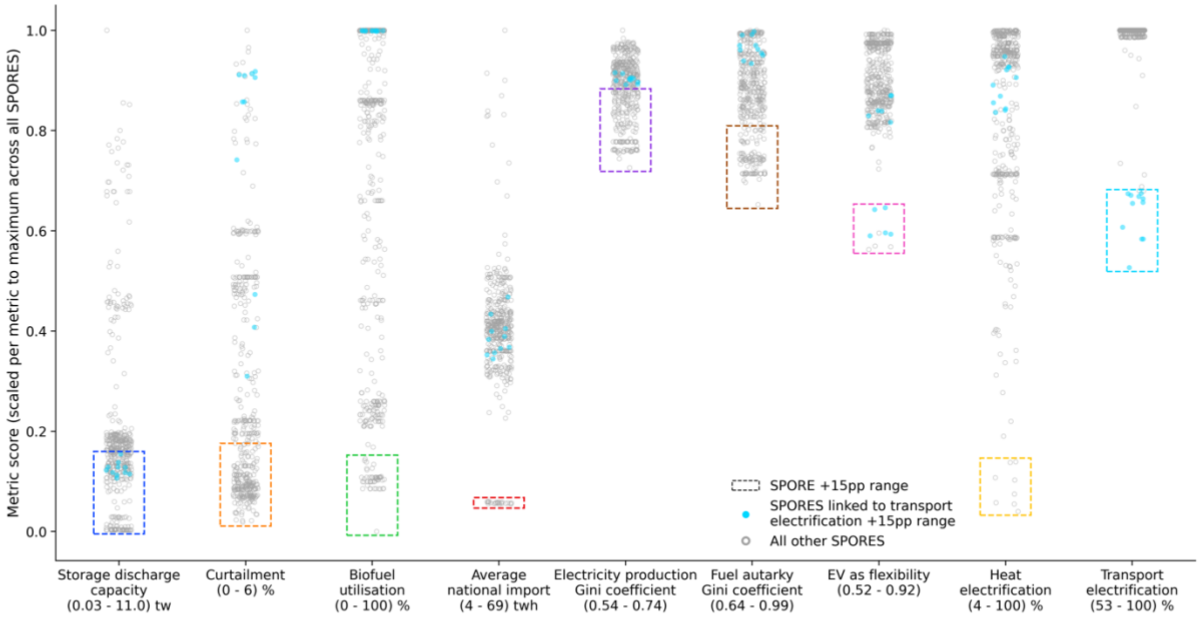

Figure S19: As with Figure 3a in the main text, but with SPORES linked to the "transport electrification" metric +15pp range highlighted.

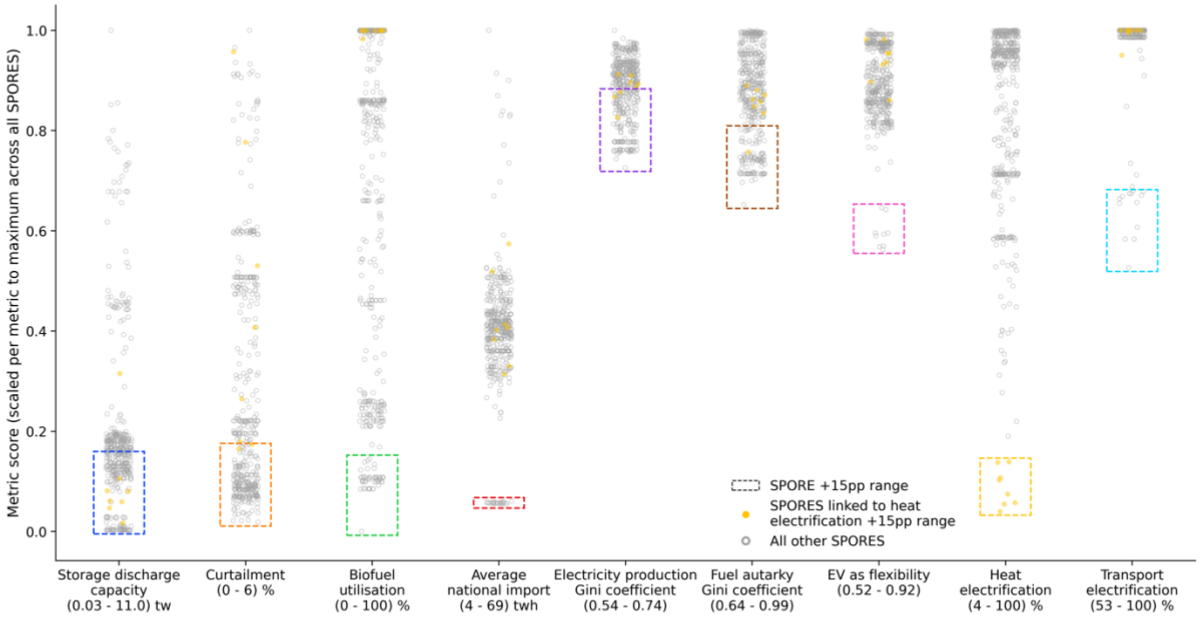

Figure S20: As with Figure 3a in the main text, but with SPORES linked to the "heat electrification" metric +15pp range highlighted.

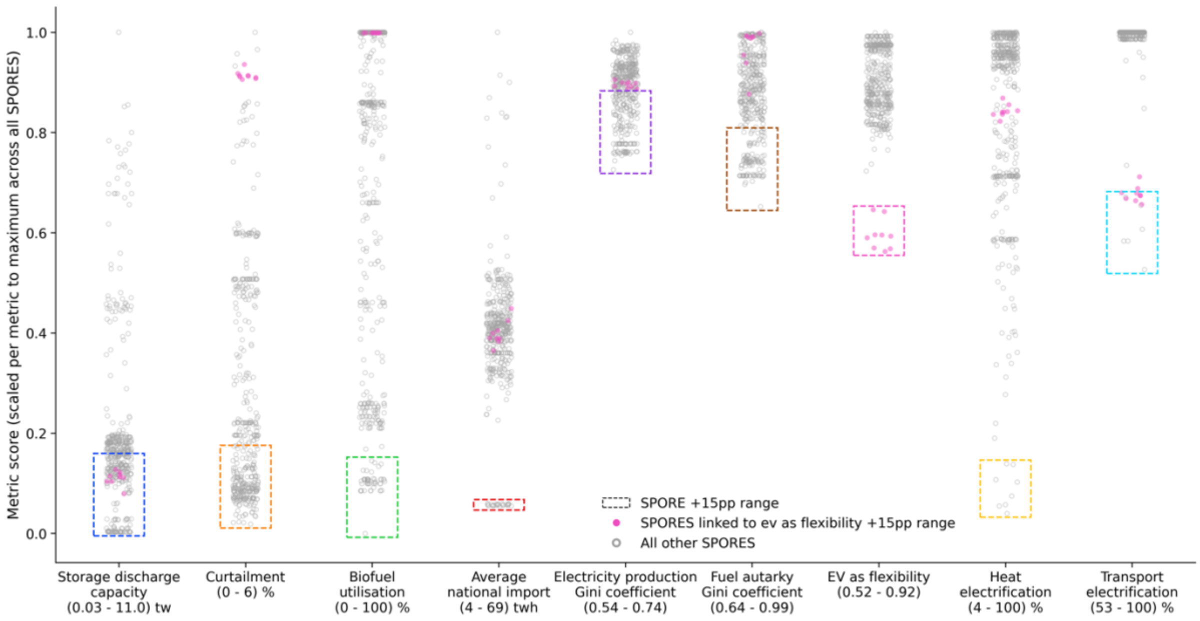

Figure S21: As with Figure 3a in the main text, but with SPORES linked to the "EV as flexibility" metric +15pp range highlighted.

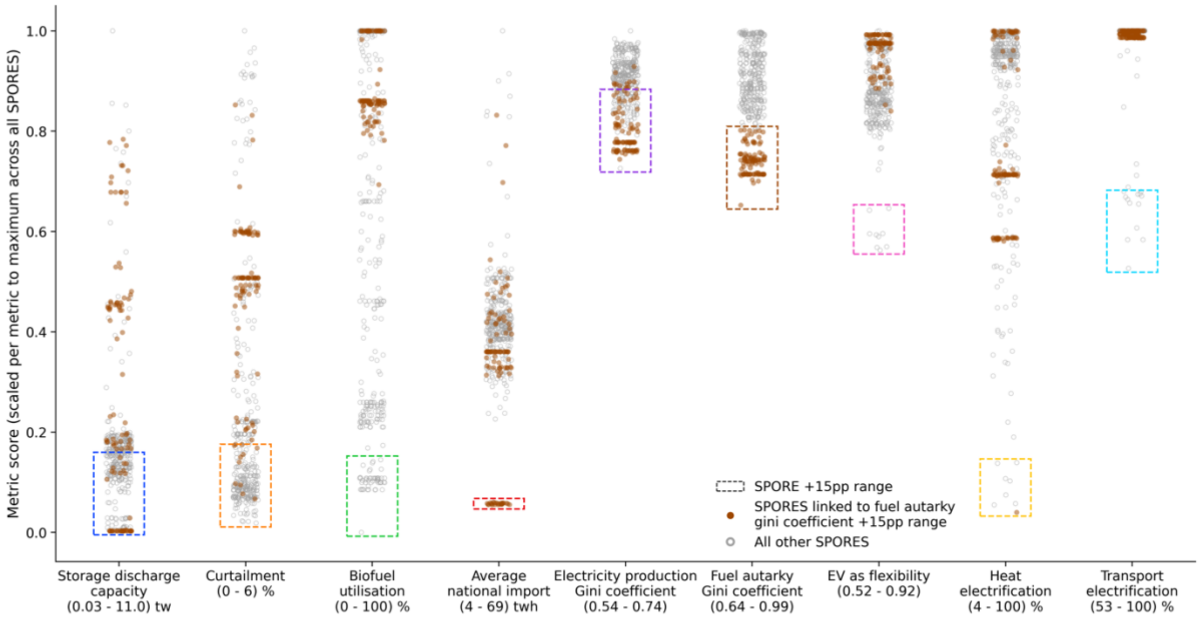

Figure S22: As with Figure 3a in the main text, but with SPORES linked to the "Fuel autarky gini coefficient" metric +15pp range highlighted.

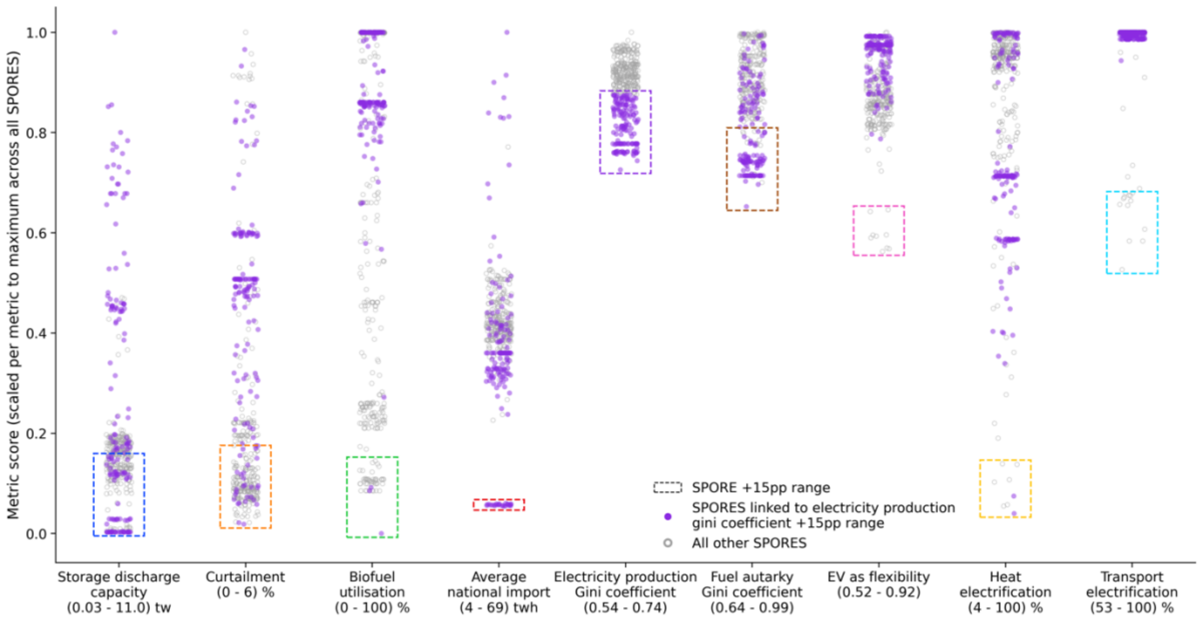

Figure S23: As with Figure 3a in the main text, but with SPORES linked to the "Electricity production gini coefficient" metric +15pp range highlighted.

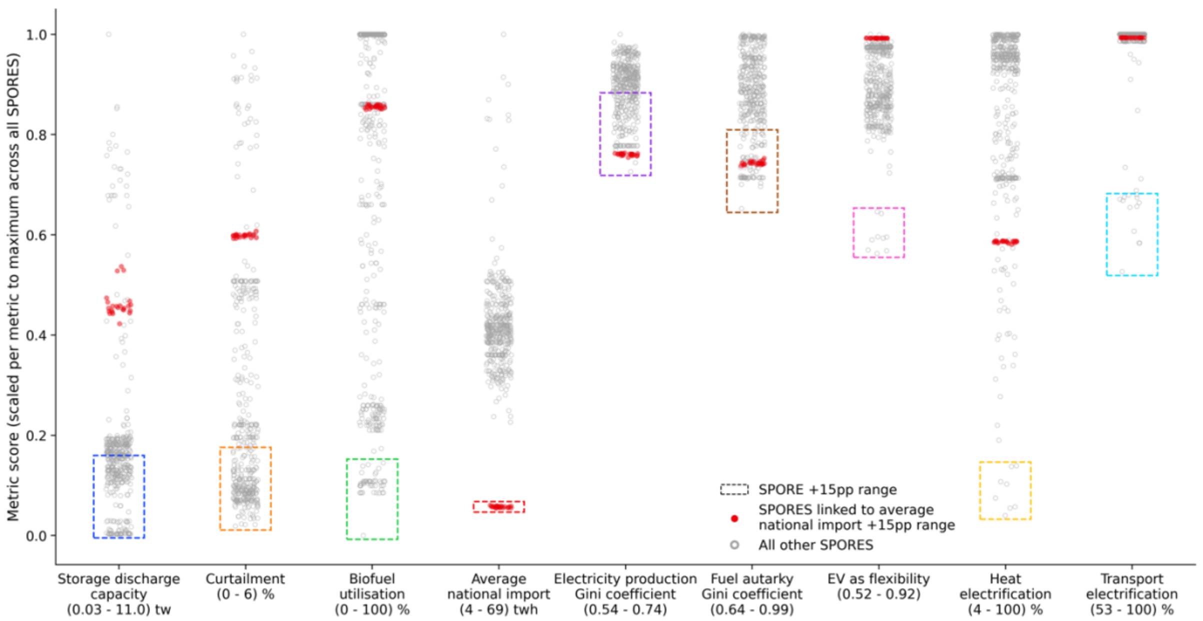

Figure S24: As with Figure 3a in the main text, but with SPORES linked to the "Average national import" metric +15pp range highlighted.

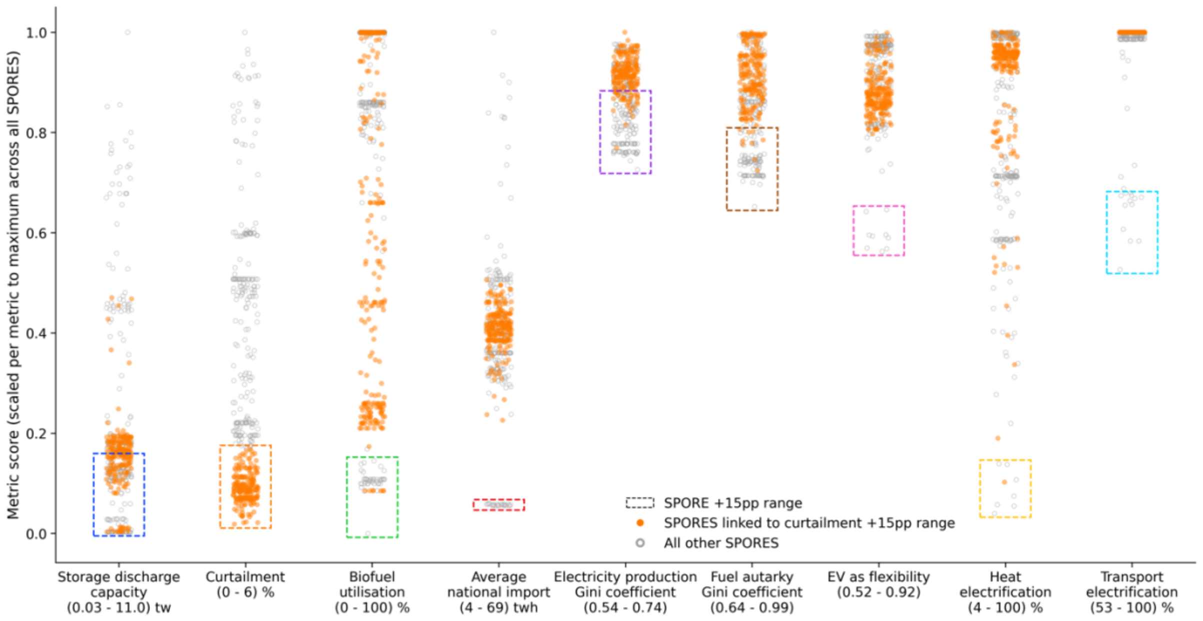

Figure S25: As with Figure 3a in the main text, but with SPORES linked to the "Curtailment" metric +15pp range highlighted.

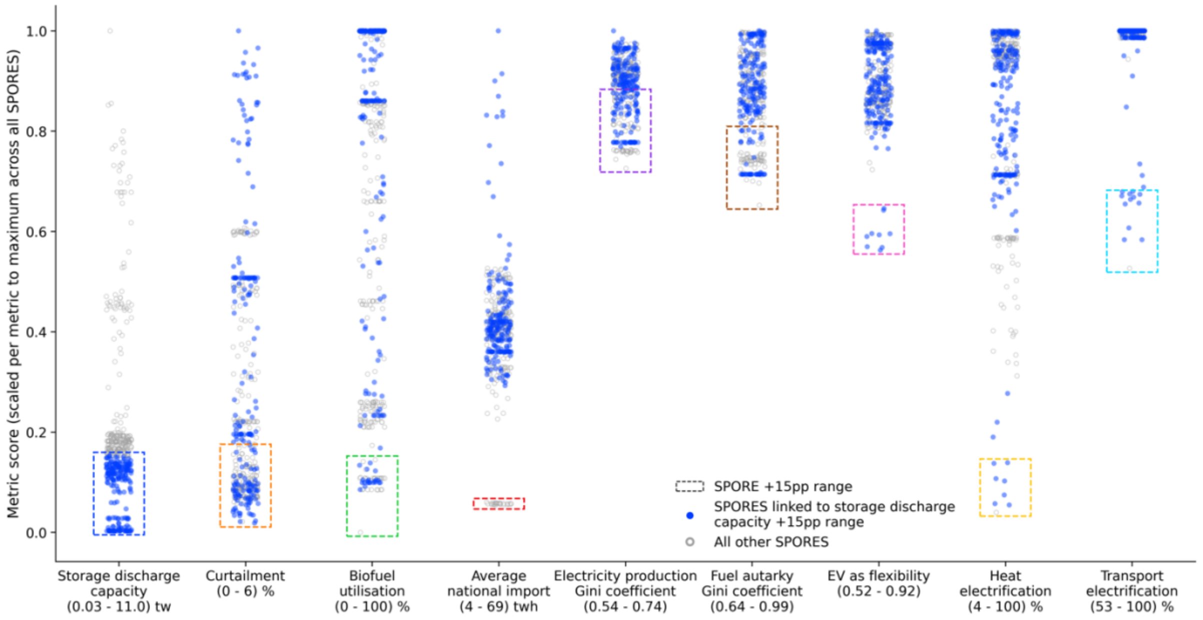

Figure S26: As with Figure 3a in the main text, but with SPORES linked to the "Storage discharge capacity" metric +15pp range highlighted.

Supplemental References

1. Tröndle, T. (2020). Euro-Calliope: pre-built models. https://zenodo.org/record/3949553.

2. Tröndle, T., Lilliestam, J., Marelli, S., and Pfenninger, S. (2020). Trade-Offs between Geographic Scale, Cost, and Infrastructure Requirements for Fully Renewable Electricity in Europe. Joule *4*, 1929–1948.

3. Mantzos, L., Wiesenthal, T., Matei, N.A., Tchung-Ming, S., Rozsai, M., Russ, P., and Ramirez, A.S. (2017). JRC-IDEES: Integrated Database of the European Energy Sector: Methodological Note (Joint Research Centre (Seville site)).

4. Kanellopoulos K., De Felice M., Hidalgo Gonzalez I., and Bocin A. (2019). JRC Open Power Plants Database (JRC-PPDB-OPEN). https://zenodo.org/record/3574566.

5. Ruhnau, O., Hirth, L., and Praktiknjo, A. (2019). Time series of heat demand and heat pump efficiency for energy system modeling. Sci Data *6*, 1–10.

6. Lombardi, F., Balderrama, S., Quoilin, S., and Colombo, E. (2019). Generating high-resolution multi-energy load profiles for remote areas with an open-source stochastic model. Energy *177*, 433–444.

7. Lombardi, F., Rocco, M.V., and Colombo, E. (2019). A multi-layer energy modelling methodology to assess the impact of heat-electricity integration strategies: The case of the residential cooking sector in Italy. Energy *170*, 1249–1260.

8. The Danish Energy Agency and Energinet (2016). Technology Data: Heating installations (The Danish Energy Agency).

9. Nouvel, R., Cotrado Sehgelmeble, M., and Pietruschka, D. (2015). European Mapping of Seasonal Performances of Air-source and Geothermal Heat Pumps for Residential Applications. In.

10. Staffell, I., Brett, D., Brandon, N., and Hawkes, A. (2012). A review of domestic heat pumps. Energy Environ. Sci. *5*, 9291–9306.

11. Gelaro, R., McCarty, W., Suárez, M.J., Todling, R., Molod, A., Takacs, L., Randles, C.A., Darmenov, A., Bosilovich, M.G., Reichle, R., et al. (2017). The Modern-Era Retrospective Analysis for Research and Applications, Version 2 (MERRA-2). Journal of Climate *30*, 5419–5454.

12. The Danish Energy Agency (2019). Technology Data - Renewable Fuels.

13. Chandrasekaran, S.R., Hopke, P.K., Newtown, M., and Hurlbut, A. (2013). Residential-Scale Biomass Boiler Emissions and Efficiency Characterization for Several Fuels. Energy Fuels *27*, 4840–4849.

14. Mermoud, F., Haroutunian, A., Faessler, J., and Lachal, B.M. (2015). Impact of load variations on wood boiler efficiency and emissions: in-situ monitoring of two boilers (2 MW and 0.65 MW) supplying a district heating system. Archives des Sciences *68*, 27–38.

15. Karunanithy, C., and Shafer, K. (2016). Heat transfer characteristics and cooking efficiency of different sauce pans on various cooktops. Applied Thermal Engineering *93*, 1202–1215.

16. Ramanathan, R., and Ganesh, L.S. (1994). A multi-objective analysis of cooking-energy alternatives. Energy *19*, 469–478.

17. Boßmann, T., and Staffell, I. (2015). The shape of future electricity demand: Exploring load curves in 2050s Germany and Britain. Energy *90*, 1317–1333.

18. Mangipinto, A., Lombardi, F., Sanvito, F.D., Pavičević, M., Quoilin, S., and Colombo, E. (2022). Impact of mass-scale deployment of electric vehicles and benefits of smart charging across all European countries. Applied Energy *312*, 118676.

19. The European Council for Automotive R&D (2019). Battery requirements for future automotive applications (EUCAR).

20. Fleiter, T., Elsland, R., Herbst, A., Manz, P., Popovski, E., Rehfeldt, M., Reiter, U., Catenazzi, G., Jakob, M., Harmsen, R., et al. (2017). Heat Roadmap Europe: Baseline scenario of the heating and cooling demand in buildings and industry in the 14 MSs until 2050.

21. Naegler, T., Simon, S., Klein, M., and Gils, H.C. (2015). Quantification of the European industrial heat demand by branch and temperature level. International Journal of Energy Research *39*, 2019–2030.

22. Rehfeldt, M., Fleiter, T., and Toro, F. (2018). A bottom-up estimation of the heating and cooling demand in European industry. Energy Efficiency *11*, 1057–1082.

23. Reiter, U., Catenazzi, G., Jakob, M., Naegeli, C., Fleiter, T., Steinbach, J., Ragwitz, M., Arens, M., Aydemir, A., Elsland, R., et al. (2016). Mapping and analyses of the current and future (2020-2030) heating/cooling fuel deployment (fossil/renewables) - Work package 1: Final energy consumption for the year 2012 (Fraunhofer Institute for Systems and Innovation Research (ISI)).

24. Madeddu, S., Ueckerdt, F., Pehl, M., Peterseim, J., Lord, M., Kumar, K.A., Krüger, C., and Luderer, G. (2020). The CO2 reduction potential for the European industry via direct electrification of heat supply (power-to-heat). Environ. Res. Lett.

25. Suopajärvi, H., Umeki, K., Mousa, E., Hedayati, A., Romar, H., Kemppainen, A., Wang, C., Phounglamcheik, A., Tuomikoski, S., Norberg, N., et al. (2018). Use of biomass in integrated steelmaking – Status quo, future needs and comparison to other low-CO2 steel production technologies. Applied Energy *213*, 384–407.

26. Mandova, H., Leduc, S., Wang, C., Wetterlund, E., Patrizio, P., Gale, W., and Kraxner, F. (2018). Possibilities for CO2 emission reduction using biomass in European integrated steel plants. Biomass and Bioenergy *115*, 231–243.

27. Material Economics (2019). Industrial transformation 2050-Pathways to net-zero emissions from EU heavy industry.

28. Word Steel Association (2019). World Steel in Figures 2019 (Brussels: Word Steel Association).

29. Mathieson, J., Rogers, H., Somerville, M., Ridgeway, P., and Jahanshahi, S. (2011). Use of biomass in the iron and steel industry - An Australian perspective. In.

30. Suopajärvi, H., Kemppainen, A., Haapakangas, J., and Fabritius, T. (2017). Extensive review of the opportunities to use biomass-based fuels in iron and steelmaking processes. Journal of Cleaner Production *148*, 709–734.

31. Vogl, V., Åhman, M., and Nilsson, L.J. (2018). Assessment of hydrogen direct reduction for fossil-free steelmaking. Journal of Cleaner Production *203*, 736–745.

32. Fischedick, M., Marzinkowski, J., Winzer, P., and Weigel, M. (2014). Techno-economic evaluation of innovative steel production technologies. Journal of Cleaner Production *84*, 563–580.

33. Worrell, E., Price, L., Neelis, M., Galitsky, C., and Zhou, N. (2007). World best practice energy intensity values for selected industrial sectors.

34. International Energy Agency (2018). The Future of Petrochemicals: Towards More Sustainable Plastics and Fertilisers (IEA Publications France).

35. Bazzanella, A., and Ausfelder, F. (2017). Low carbon energy and feedstock for the European chemical industry (DECHEMA, Gesellschaft für Chemische Technik und Biotechnologie eV).

36. Saebea, D., Ruengrit, P., Arpornwichanop, A., and Patcharavorachot, Y. (2020). Gasification of plastic waste for synthesis gas production. Energy Reports *6*, 202–207.

37. Liljenström, C., and Finnveden, G. (2015). Data for separate collection and recycling of dry recyclable materials (KTH Royal Institute of Technology).

38. AISBL, P. (2019). Plastics–the Facts 2018. Information accessed at http://www.-plasticseurope.org/application.

39. Boulamanti, A., and Moya, J.A. (2017). Production costs of the chemical industry in the EU and other countries: Ammonia, methanol and light olefins. Renewable and Sustainable Energy Reviews *68*, 1205–1212.

40. Fasihi, M., Efimova, O., and Breyer, C. (2019). Techno-economic assessment of CO2 direct air capture plants. Journal of Cleaner Production *224*, 957–980.

41. Lombardi, F., Pickering, B., Colombo, E., and Pfenninger, S. (2020). Policy Decision Support for Renewables Deployment through Spatially Explicit Practically Optimal Alternatives. Joule *0*.

42. Anderski, T., Surmann, Y., Stemmer, S., Grisey, N., Momot, E., Leger, A.-C., Betraoui, B., and van Roy, P. (2014). European cluster model of the Pan-European transmission grid (e-HIGHWAY 2050).

43. Department for Business, Energy & Industrial Strategy (2019). Sub-national Electricity and Gas Consumption: Regional and Local Authority, Great Britain, 2018.

44. De Felice, M., and Kavvadias, K. (2020). energy-modelling-toolkit/hydro-power-database: JRC Hydro-power database - release 07 (Zenodo).

45. Geth, F., Brijs, T., Kathan, J., Driesen, J., and Belmans, R. (2015). An overview of large-scale stationary electricity storage plants in Europe: Current status and new developments. Renewable and Sustainable Energy Reviews *52*, 1212–1227.

46. Pianosi, F., Beven, K., Freer, J., Hall, J.W., Rougier, J., Stephenson, D.B., and Wagener, T. (2016). Sensitivity analysis of environmental models: A systematic review with practical workflow. Environmental Modelling & Software *79*, 214–232.

47. Pietzcker, R.C., Osorio, S., and Rodrigues, R. (2021). Tightening EU ETS targets in line with the European Green Deal: Impacts on the decarbonization of the EU power sector. Applied Energy *293*, 116914.

48. Stöckl, F., Schill, W.-P., and Zerrahn, A. (2021). Optimal supply chains and power sector benefits of green hydrogen. Sci Rep *11*, 14191.

49. Zeyringer, M., Price, J., Fais, B., Li, P.-H., and Sharp, E. (2018). Designing low-carbon power systems for Great Britain in 2050 that are robust to the spatiotemporal and inter-annual variability of weather. Nat Energy *3*, 395–403.

50. Güneralp, B., Zhou, Y., Ürge-Vorsatz, D., Gupta, M., Yu, S., Patel, P.L., Fragkias, M., Li, X., and Seto, K.C. (2017). Global scenarios of urban density and its impacts on building energy use through 2050. Proc Natl Acad Sci U S A *114*, 8945–8950.
